# Know thy immune self and non‐self: Proteomics informs on the expanse of self and non‐self, and how and where they arise

**DOI:** 10.1002/pmic.202000143

**Published:** 2021-08-09

**Authors:** Sebastian Joyce, Nicola Ternette

**Affiliations:** ^1^ Department of Veterans Affairs Tennessee Valley Healthcare System and the Department of Pathology Microbiology and Immunology Vanderbilt University Medical Center Nashville Tennessee USA; ^2^ Centre for Cellular and Molecular Physiology Nuffield Department of Medicine University of Oxford Oxford UK

**Keywords:** antigen presentation, antigen processing, human leukocyte antigen, immunopeptidomics, major histocompatibility complex, mass spectrometry, T cell epitope

## Abstract

T cells play an important role in the adaptive immune response to a variety of infections and cancers. Initiation of a T cell mediated immune response requires antigen recognition in a process termed MHC (major histocompatibility complex) restri ction. A T cell antigen is a composite structure made up of a peptide fragment bound within the antigen‐binding groove of an MHC‐encoded class I or class II molecule. Insight into the precise composition and biology of self and non‐self immunopeptidomes is essential to harness T cell mediated immunity to prevent, treat, or cure infectious diseases and cancers. T cell antigen discovery is an arduous task! The pioneering work in the early 1990s has made large‐scale T cell antigen discovery possible. Thus, advancements in mass spectrometry coupled with proteomics and genomics technologies make possible T cell antigen discovery with ease, accuracy, and sensitivity. Yet we have only begun to understand the breadth and the depth of self and non‐self immunopeptidomes because the molecular biology of the cell continues to surprise us with new secrets directly related to the source, and the processing and presentation of MHC ligands. Focused on MHC class I molecules, this review, therefore, provides a brief historic account of T cell antigen discovery and, against a backdrop of key advances in molecular cell biologic processes, elaborates on how proteogenomics approaches have revolutionised the field.

**Abbreviations**
MHC: Major histocompatibility complex
HLA: Human leukocyte antigen
H‐2: Histocompatibility 2
DC: Dendritic cell
β2m: β2‐microglobulin
ER: Endoplasmic reticulum
TAP: Transporter associated with antigen processing
TAPBPR: TAP‐binding protein relate
ERAP: Human ER‐associated aminopeptidase associated with antigen processing
ERAAP: Mouse ER‐associated aminopeptidase associated with antigen processing
VSV: Vesicular stomatitis virus
VACV: Vaccinia virus
IAV: Influenza A virus
IFN: Interferon
LMP: Low Molecular mass Polypeptide
MECL: Multicatalytic Endopeptidase Complex‐Like

## 1 INTRODUCTION

The immune system consists of two arms—the innate and the adaptive immune systems—that work in concert to sense alteration(s) in the internal milieu, to process the perceived information, and to actuate a response tailored to the altered state(s). Most times, the response of the innate immune system is sufficient to return the host's internal milieu back to normalcy; when not, the adaptive immune system is engaged. One end‐product of the innate immune response is the display of fragments—fragments derived from agents that incite alterations in the host's internal milieu—on the surface of certain innate immune cells called dendritic cells (DCs). Such fragmentary end‐products are recognised by T lymphocytes to initiate an adaptive immune response.

T cells play an important role in the adaptive immune response to a variety of infections and cancers. Initiation of a T cell mediated immune response requires antigen recognition in a process termed MHC (major histocompatibility complex) restriction. A T cell antigen is a composite structure made up of a peptide fragment bound within the antigen‐binding groove of an *MHC* (*Major Histocompatibility Complex*)‐encoded class I (MHC‐I) or class II (MHC‐II) molecule. The chemical features of the peptide ligands presented by MHC molecules, their source(s), and their generation are hotly pursued. The discoveries so made have led to the elucidation of the basic molecular and cellular principles of antigen processing and presentation, and to rational vaccine design and therapies against infectious diseases and cancers. How advances in mass spectrometry, and proteomics technologies and platforms coupled with genomics approaches have led to basic understanding of antigens presented by T cells are narrated below. The focus is on MHC‐I immunopeptidomes—the collection of peptides presented by a given MHC‐I displayed on the surface of a cell—because of their pivotal role in CD8^+^ T cell mediated immune surveillance against intracellular pathogens and cancers. This focus notwithstanding, we do acknowledge that MHC‐II immunopeptidomes and their roles in CD4^+^ T cell mediated immune surveillance against intracellular pathogens and cancers are important but are not discussed herein.

## 2 ESSENTIAL HISTORY OF THE FIELD

The 1980s and 1990s were exciting times for students of antigen processing and presentation, and T cell biology. By this time immunologists and geneticists had established that the antigen(s) coded by the *MHC* controlled allogeneic skin and tumour graft rejection both in mice and men [1, 2]. As well, the 70s witnessed the first descriptions of MHC restriction [3, 4]—a process that controlled host T and B cell responses to proteins, viruses, and bacteria. These two seemingly distinct immunologic recognition processes needed a biochemical definition. By the late 1970s and early 1980s Nathenson and colleagues had devised ways to cleave MHC‐I molecules from cell surfaces and adapted a radiochemical method which, coupled with Edman degradation, unveiled the first primary structure of an MHC molecule—H‐2K^b^ (H‐2, histocompatibility‐2, the MHC of the mouse). Immediately thereafter, primary structures of several other MHC molecules were determined [5, 6].

Having unraveled the primary structures of several mouse and human MHC‐I and MHC‐II molecules, the stage was set to elucidate the biochemical basis of MHC restriction. Prior to this, the works of Unanue and colleagues had revealed that the activities of T lymphocytes were intimately linked to their interactions with macrophages [7, 8, 9], and the independent works of Unanue and colleagues, and Grey and coworkers demonstrated that the macrophage‐T cell intimacy was to process antigens [7, 12]. So also, it was known that nucleo‐cytoplasmic proteins, notably the SV40 T antigen and influenza A nucleoprotein and derived peptides, or proteins deliberately delivered to the cytosol by fusion of non‐replicative influenza A virus or by osmotic shock (e.g., ovalbumin) were targets of MHC‐I restricted CD8^+^ T cells [13, 14, 15, 16, 17]. The in vitro binding studies that followed [18, 19, 20, 21] and the solution of the three‐dimensional structure of a human MHC‐I molecule—human leukocyte antigen (HLA) class I molecule—HLA‐A*02:01 [22, 23], revealed that the MHC was a receptor for processed peptides with a single binding site [24, 25, 26, 27]. The question now became, what sorts of peptides do MHC molecules bind and display to T cells in vivo? This was a burning question for MHC and T cell enthusiasts in the mid to late 1980s and early 1990s.

The radiochemical approach—invented to determine the amino acid sequences of peptides and proteins that were available in limited quantities [6]—returned yet another time to unveil the biology of MHC molecules. The first three‐dimensional structure of A*02:01 had revealed that the binding site was occupied by a conglomerate of ligands whose chemical identities eluded Bjorkman, Strominger, Wiley and colleagues [22]. They postulated, and the general notion that followed was, that not a few or several but numerous peptides were bound in that A*02:01 antigen‐binding groove indicating that the isolation of associated ligands in sufficient quantities to permit amino acid sequence determination by Edman method would be challenging. Hence, Nathenson and Van Bleek reasoned that if cells infected with a virus that shuts off host protein synthesis (*a la* vesicular stomatitis virus, VSV) were tagged with radiolabelled amino acids, the tag would get incorporated into newly synthesised viral proteins. The peptides processed from the radiolabelled viral proteins would then be available for binding to MHC class I molecules. Such peptides could then be isolated from the restricting MHC‐I molecule and subjected to Edman sequencing. Indeed, the skilled execution of this experiment revealed one of the first peptide antigen isolated from an MHC molecule: the VSV N protein‐derived RGYVYQGL—coining the term ‘naturally processed’ to indicate the cellular source of the antigen as opposed to synthetic [28]! Concurrently, Rammenssee and colleagues, deploying a completely different approach, had acid (trifluoroacetic acid) extracted, specific influenza virus‐derived peptides from whole infected cells without the need for MHC purification and determined the identities of the two distinct peptides that were presented by H‐2K^d^ and H‐2D^b^—the two mouse MHC‐I molecules [29, 30, 31, 32]. Similarly, several groups showed peptide binding and presentation by MCH‐II molecules [33, 34, 35, 36, 37]. All of these studies culminated in a molecular definition of MHC restriction. That is, MHC restriction entailed the display of proteolytically processed short peptide fragments of self and nonself proteins by the antigen‐presenting MHC molecules in a manner recognizable by T cells.

These initial reports were shortly followed by direct amino acid sequence determination of individual peptides eluted from the MHC with the aid of the mass spectrometer [36, 37, 38]. A critical early application of this technology led to the discovery of antigenic phosphopeptides, which now have found use in cancer immunotherapy [39, 40, 41, 42, 43, 44]. Advances in mass spectrometers and proteomics technologies and platforms have since paved the way to directly elucidate the amino acid sequences of antigenic peptides [45, 46, 47, 48, 49, 50, 51, 52]. Nonetheless, the nature of naturally processed peptide antigens derived from numerous re‐emerging and newly emerging pathogens—for example, Dengue, Marburg, Ebola, *Mycobacterium tuberculosis*, *Plasmodium vivax*, and *Plasmodium falciparum*—yet remains. This knowledge is a prerequisite to track T cell mediated protective immunity in experimental models and in vaccine trials in humans.

## 3 ESSENTIAL STRUCTURAL BASIS OF ANTIGEN PRESENTATION

The nature of the peptides and antigens presented by MHC molecules depend on the physico‐chemical features of the antigen‐binding groove. Here the focus is on MHC‐I molecules and the peptides they present. A landmark advance in our understanding of the basis of MHC‐restricted antigen recognition by T cells was the solution of the three‐dimensional structure of A*02:01 by X‐ray crystallography [22, 23, 53]. MHC‐I is a heterodimer made of a heavy chain—coded for by the *MHC‐I* genes that is noncovalently associated with the light chain β2‐microglobulin (β2m). The heavy chain folds into three domains: the extracellular α1, α2 and α3 domains, which are membrane anchored by the transmembrane region that ends in a short cytoplasmic tail. The three‐dimensional structure of A*02:01 revealed that the α1 and α2 domains of the heavy chain folds into a super‐domain to form the antigen‐binding groove: two antiparallel α‐helices confine the lateral sides of the antigen‐binding groove with the two β‐sheets, each made up of four antiparallel β‐strands, supporting the bottom. The membrane proximal immunoglobulin‐like α3 domain and β2m support the α1 and α2 super‐domain [22, 23, 53].

The first MHC‐I structure revealed, in addition to the heavy‐ and light‐chain electron density, extra electron density within the antigen‐binding groove that could not be assigned a structure. As it was known that MHC molecules presented antigens in the form of peptides, it was speculated that the third component (the extra electron density) consisted of a conglomerate of peptides derived from proteins of the host cell that expressed A*02:01 used in the structural studies [22]. Another key insight gleaned from this first structure was that the antigen‐binding grove contained six pockets—pockets A through F. Of these pockets, pocket A and, to some extent, pocket F were made of conserved amino acid residues. Side chains of these conserved residues made conserved main chain interactions with the amino‐terminal amine, and carboxy‐terminal carbonyl oxygen and hydroxyl groups. In contrast to the conserved pockets A and F, pockets B to E were made of highly variable amino acid residues that were encoded by polymorphisms that distinguished each *MHC‐I* allele from the other even across vertebrate species. Thus, the good majority of the amino acid alterations that distinguished the MHC‐I heavy chains mapped to the antigen‐binding groove [53]. The resulting physico‐chemical architecture of the antigen‐binding grove, therefore, dictated the nature of the peptides presented by a given MHC‐I molecule.

### 3.1 HLA‐I alleles and peptide binding motifs

To understand what feature/s in an antigen dictated its presentation by an MHC‐I molecule and not the others, Rammensse and coworkers devised a simple but clever experiment. They immunoprecipitated different mouse MHC‐I molecules with specific monoclonal antibodies and acid eluted associated ligands. After separating the low molecular mass ligands associated with the MHC‐I—presumably those that led to the extra density in the structure described above, the pooled ligands—were subjected to Edman degradation. This experiment revealed that the ligands bound to the MHC‐I were indeed peptides, and that they were short, made up of 8—9 amino acid residues in length. The most astounding revelation was, depending on the presenting MHC‐I, the peptides contained two to three conserved residues at defined positions—that is, peptides bound to H‐2K^b^ contained a structurally invariant phenylalanine or tyrosine at position 5 and a hydrophobic, aliphatic residue—such as leucine, isoleucine, or valine—at the carboxy‐terminus, and, similarly, those bound to H‐2D^b^ contained an invariant asparagine at position 5 and a hydrophobic, aliphatic residue—such as methionine or isoleucine—at the carboxy‐terminus. And that the remaining positions within the peptide accommodated one of the 20 naturally occurring amino acid residues. Hence, peptides bound to an MHC‐I contained a binding motif made of an internal and a terminal anchor residues [32]. In conclusion, a given MHC‐I molecule can bind theoretically over a tenth‐of‐a‐billion (∼20^6^ 8‐mers) to a billion (∼20^7^ 9‐mers) peptides that are structurally related at the anchors. So, then, if a cell displays ∼50–100 thousand MHC‐I at the surface, is there a need to present millions‐and billions (as Carl Sagan would say about the stars in the sky!) of peptides? New molecular cell biology seems to hold some of the secrets to this question, perhaps to represent the internal milieu at the cell surface for an appraisal by T cell and to keep immune reactions against self in check.

### 3.2 Excursion: Evolution of HLA‐I peptidome diversity

The enormous capacity of MHC alleles to accommodate such high numbers of peptide ligands is motivated by the ability to cover the proteome diversity of pathogens. It is generally thought that the high polymorphism in the HLA locus is selected and maintained through a ’molecular arms race‘ [54, 55, 56]. In fact, characterization of immunopeptidomes of 18 individuals revealed that peptides bound to 27 highly prevalent HLA‐I molecules were derived from 10% of the expressed genome. This ‘hotspot’ of self‐presentation was driven by the HLA‐I genotype of the individual, and increased promiscuity conveyed an improved coverage of self‐protein presentation [57, 58, 59].

Further evidence for overall benefit of MHC diversity and antigenic coverage are found in the analyses of determinants of positive immunotherapeutic cancer treatment outcomes: An increased MHC‐associated peptide diversity, and accompanied increased probability of presentation of neoantigens are a strong determinant of the outcome of immune checkpoint blockade in cancer [60, 61]. Loss of heterozygosity in the HLA locus, leading to a restricted MHC allele diversity in the tumour, are a prevalent tumour escape mechanism and is associated with poor outcomes in checkpoint blockade therapy [62, 63].

Whilst the expression levels of MHC‐I are controlled by transcription, translational, and posttranslational mechanisms [64, 65], expression levels of certain HLA alleles may be inversely correlated with their ability to present a larger variety of peptide sequences, leading to higher expression of alleles that are more ’fastidious‘ [66]. Despite this result being counter intuitive, it emphasizes the importance of evaluating quantitative aspects of antigen presentation and recognition. Insights so gained may unveil cause(s) and selection (evolution) of HLA diversity.

### 3.3 HLA‐I supertypes and peptide binding supermotifs

Extensive HLA‐Ia gene polymorphism is a major impediment to rational design of T cell‐taergeted vaccines and are barriers to tissue transplantation [67, 68, 69, 70]. There are over 9,300 HLA‐I allotypes recorded, and there are numerous variants [71]. Consequently, the antigen‐binding groove of numerous allelic products will have a unique physico‐chemical architecture [53, 68] and, thereby, dictate the motif required for an epitope to bind it [72]. Because patterns of T cell epitope presentation and immune recognition in a given infection are different for individuals expressing different HLA molecules, development of universal T cell vaccine is a challenge.

Brilliantly, Sette and colleagues as well as Buus and co‐workers [68, 69, 70, 73, 74, 75] discovered that all the currently known HLA‐A and HLA‐B molecules can be grouped into functional ’supertypes‘ predicated on pockets B and F of members of each supertype having a shared physico‐chemical architecture [70]. Pockets B and F accommodate the dominant peptide anchors of HLA‐I restricted epitopes: that is, the middle anchor at position 2 and the C‐terminal anchor [72]. The discovery of HLA supertypes led to the description of common binding motifs within peptides that bind a supertype and are collectively called ’supermotifs‘ [68, 70]. Most importantly, peptide ligands predicted based on algorithms that have taken into account supermotifs have led to the discovery of numerous virus‐derived CD8^+^ T cell epitopes [76, 77, 78, 79, 80]. A recent in‐depth study of naturally processed immunopeptidomes of 95 distinct HLA‐A, HLA‐B and HLA‐C molecules by high‐resolution mass spectrometry has further refined supertypes based on HLA‐I binding submotifs. These 95 HLA‐I studied are expressed by 95% of the human population. In doing so, a significant number of HLA‐I did not fit into a supertype or have been removed from previous supertypes [49]. That notwithstanding, targeting commonly recognised epitopes by T cells of individuals of the same HLA‐I supertype holds promise as a vaccine design strategy.

## 4 A NOTE ON METHODS

Given that a single allelic MHC‐I can bind millions of peptides, can each one to the last one in the antigen binding groove be extracted and identified? The answer is no because of limitations of the best of detergents to extract proteins from the cell membranes, efficiencies of downstream MHC‐I purification and peptide elution methods, and the sensitivities and accuracies of detection, which currently uses state‐of‐the art mass spectrometers coupled with genomics and proteomics (henceforth proteogenomics) approaches. Do we really need to know the features of the last peptide in the groove? The answer is no, not unless the biology demands it! And in the case of T cells, it is sufficiently sensitive that it can go about its activities by seeing a single pMHC complex, or—if this is a bit too exaggerated—10—100 pMHC molecules per cell suffices [81, 82, 83, 84]!

Three peptide isolation methods are used to define immunopeptidomes: (a) peptides isolated directly from immunoprecipitated or affinity purified pMHC complexes [28, 32, 38, 85]; (b) low molecular weight peptide fraction from total acid extracts of cells [29, 30, 31, 86, 87]; (c) mild acid extracts of peptides from cell surfaces [88, 89, 90]. Each of these peptide extraction methods has its own advantages and disadvantages, but, when used in combination, the approaches are complementary and yield significant information about the immune self and non‐self [87].

Traditionally, the extracted peptides were fractionated by reversed‐phase chromatography and amino acid sequence determined by Edman degradation [28, 32, 34, 35, 85, 91, 92, 93]. The Edman approach required ∼5–10 nanomole (∼0.5 mg) amounts of purified pMHC‐I complexes for reliable sequence determination. Hence, several groups developed ways to generate soluble pMHC‐I complexes which are then affinity purified from cell culture supernatants [92, 94]. Some groups continue to use this approach for T cell epitope discovery [45, 47, 95, 96]. Whilst soluble pMHC‐I complexes provide large, steady supply of pMHC‐I, its utility is limited when, for example, the immunopeptidomes of primary cells, both normal and cancer, are sought. Furthermore, the truncation of MHC‐I without or with carboxy‐terminal sequence modification has raised questions related to the validity of the immunopeptidomes that assemble soluble MHC‐I in cells. Three lines of evidence suggest that the quality of the immunopeptidomes is not compromised: (1) At body temperature, the temperature at which cells secreting pMHC‐I are cultivated, most MHC‐I will have avidly bound cargo, because loosely bound cargo dissociate easily and will not bind conformation‐dependent monoclonal antibody used for affinity purification [97, 98]. (2) Cell‐free assembly experiments have shown soluble HLA‐B8‐β2m heterodimers load peptides in the presence of purified tapasin‐ERp57 conjugates [99]. (3) Epitopes isolated from soluble pMHC complexes are recognised by human CD8^+^ T cells or by HLA‐I transgenic mouse CD8^+^ T cells [45, 100, 101]. A good number of such epitopes are also protective in lethal challenge experiments in mice [45].

Recent advances in mass spectrometry and bioinformatics do not require tedious manipulation of cells for immunopeptidomics studies, and allow the direct interrogation of primary tissue/s obtained from patients. Importantly, such approaches allow direct comparison of immune landscapes in healthy versus diseased tissues, that is, in infectious diseases, autoimmune diseases, and cancer. Such analyses have rapidly expanded the field of cancer and led the discovery of numerous cancer‐specific HLA‐ligands, some of which are immunogenic (see Box 1 for a definition) and used as personalised cancer vaccine [102, 103, 104, 105, 106, 107, 108, 109].

**Box 1: Learning ImmunoSpeak with simple experiments**

***Antigen***, an agonistic substance recognised by lymphocyte receptors—for example, the T cell receptor in the context of this review—but also the B cell receptor, and antibody/immunoglobulin; as such not all antigens are immunogens.

***Determinants***, (archaic, ca. 1970s, 80s, 90s!) all peptides that bind to and are presented by MHC molecules; also called epitopes sensu lato in the current literature.

***Epitope***, sensu stricto, that aspect of an antigen that is recognised by a T or a B cell receptor.

***Immunogen***, an agonistic substance that elicits (induces/provokes) a T or a B cell response in a vertebrate host organism or in an in vitro culture model. As such, all immunogens are antigens, but not all antigens are immunogens.

***Protective antigens***, a pathogen‐derived immunogen which elicits a T or a B cell response in a vertebrate host and confers protection to the pathogen when challenged with a lethal dose of the pathogen from which the immunogen was derived but not to the pathogen that does not express the immunogen.

Consider the following experiments: In the first, a group of mice were inoculated by the intraperitoneal (i.p.) route with a virus, say, vaccinia virus (VACV; Case Western Reserve strain), the vaccine against smallpox. Seven days later, spleens were harvested and screened with a panel of >50 peptides eluted from MHC‐I molecules expressed by VACV‐infected HeLa cells. VACV‐reactive CD8+ T cells recognised a small subset of the peptides in the panel as evidenced by IFN‐ γ secretion in an ELISpot assay or by tracking the response with pMHC tetramers (see ref. [45]). Peptides recognised in such an experiment are called antigens. Such peptide *antigens* are also T cell *epitopes*. The remainder of the peptides in the panel not recognised by the T cell receptor are called *determinants* and not epitopes as is in the current literature.

In the second experiment, two VACV‐derived proteins, *x* and *y* —which contain two antigenic peptides *x’* and *y’* discovered in the experiment above—were used as immunogens in prime‐boost immunization of mice by i.p. route. After 14–72 days post boost, CD8+ T cells so elicited recognised the peptide *x’* derived from the immunizing antigen *x* but not the other peptide *y’*, and vice versa (see refs. [45, 347]). Hence, these two antigens are immunogens; in this example, the two immunogens are antigens as well.

In the third experiment, mice were prime‐boost vaccinated 2 weeks apart with proteins *x* and *y*. After 14–72 days post boost, mice were challenged with a lethal dose of VACV via the intranasal route. Whilst both groups of mice elicited an immunogen‐specific CD8+ T cell response, only mice prime‐boost immunised with protein *x* survived the challenge, but the group that received protein *y* as the immunogen did not (see refs. [45, 348]). Hence, *x* is a protective antigen, but *y* is not, even though both *x* *and y immunogens are derived from VACV*.

Because native pMHC‐I are cell membrane bound, they are extracted with the use of detergents. A careful recent study demonstrated that the choice of detergents used in membrane extraction impacted the quality and quantity of peptides identified. The zwitter ionic CHAPS ([3‐([3‐cholamidopropyl] dimethylammonio)‐1‐propanesulfonate]) fared the best in comparison to the ionic sodium deoxycholate, non‐ionic IGEPAL CA‐630 and Triton‐X100 [110, 111]. So also, different peptide enrichment methods after dissociation from MHC‐I impacted the quality and quantity of peptides identified. In this case, reversed‐phase chromatography using a C18 silica matrix fared best when compared to the traditional ultrafiltration across a cellulose membrane. These biases were HLA‐I allele dependent as well [90, 110, 111, 112]. These critical methodologic observations suggest that a comprehensive and complete characterization of immunopeptidomes will require experiments that use multiple extraction and enrichment methods to cater to the physicochemical demands of each peptide or a collection of peptides in the test immunopeptidome, and reminds us of the fact that immunopeptidomics studies to date identify a proportion of the immune landscapes of cells, and that the full complexity of the presented peptidome has yet to be understood. Further, these observations should cause pause when evaluating data, especially contradictory ones! However, the analysis of MHC peptidomes in the context of a fully complex, membrane‐bound MHC haplotype with up to six classical alleles expressed in humans, introduce not only challenges regarding the peptide sequence variety, but introduces the challenge of assigning the identified peptide ligands to the originating MHC molecule. The solution of this challenge has since been approached rapidly by the field, and Gibbs clustering tools as well as binding predictions can assist these stratifications. These approaches will benefit from the recent mass spectrometric profiling of HLA‐I associated peptidomes in mono‐allelic cells because the databases so created enables accurate peptide assignments and epitope prediction [45, 49, 113].

## 5 BRIEF TOPOLOGICAL BIOCHEMISTRY OF ANTIGEN PROCESSING AND PRESENTATION

### 5.1 The basics

The process by which MHC‐I molecules assemble, traffic, and display peptides is an excellent example of how a macromolecule utilizes the cell's topological biochemistry for antigen processing and presentation. Being a type I integral membrane glycoprotein, MHC‐I molecules assemble in the endoplasmic reticulum (ER) [114, 115]. Whilst the heavy and light chains are co‐translationally inserted into the ER owing to their N‐terminal signal sequences, the peptide component of the MHC‐I molecule is actively transported into this vesicular compartment by accessory protein channels [116, 117, 118, 119, 120]. Peptides that assemble with MHC‐I molecules are predominantly of cytosolic origin, but ER, nuclear, mitochondrial and phagosomal/lysosomal proteins also contribute to the peptide pool. Regardless of their origin, MHCI‐binding peptides meet in the cytosol prior to entry into the ER. The assembly of the MHC‐I molecule is a complex highly concerted and controlled process that ensures cell surface display of only those molecules that are assembled with high affinity peptides (reviewed in ref. [121]). Display of peptide‐associated MHC‐I molecule at the cell surface is essential as this pathway of antigen presentation evolved to apprise CD8^+^ T cells of cytosolic events so as to provide a mechanism to safeguard cells from intracellular invasion by viruses and bacteria, and from tumorigenic mutations.

### 5.2 The assembly line

**FIGURE 1:**
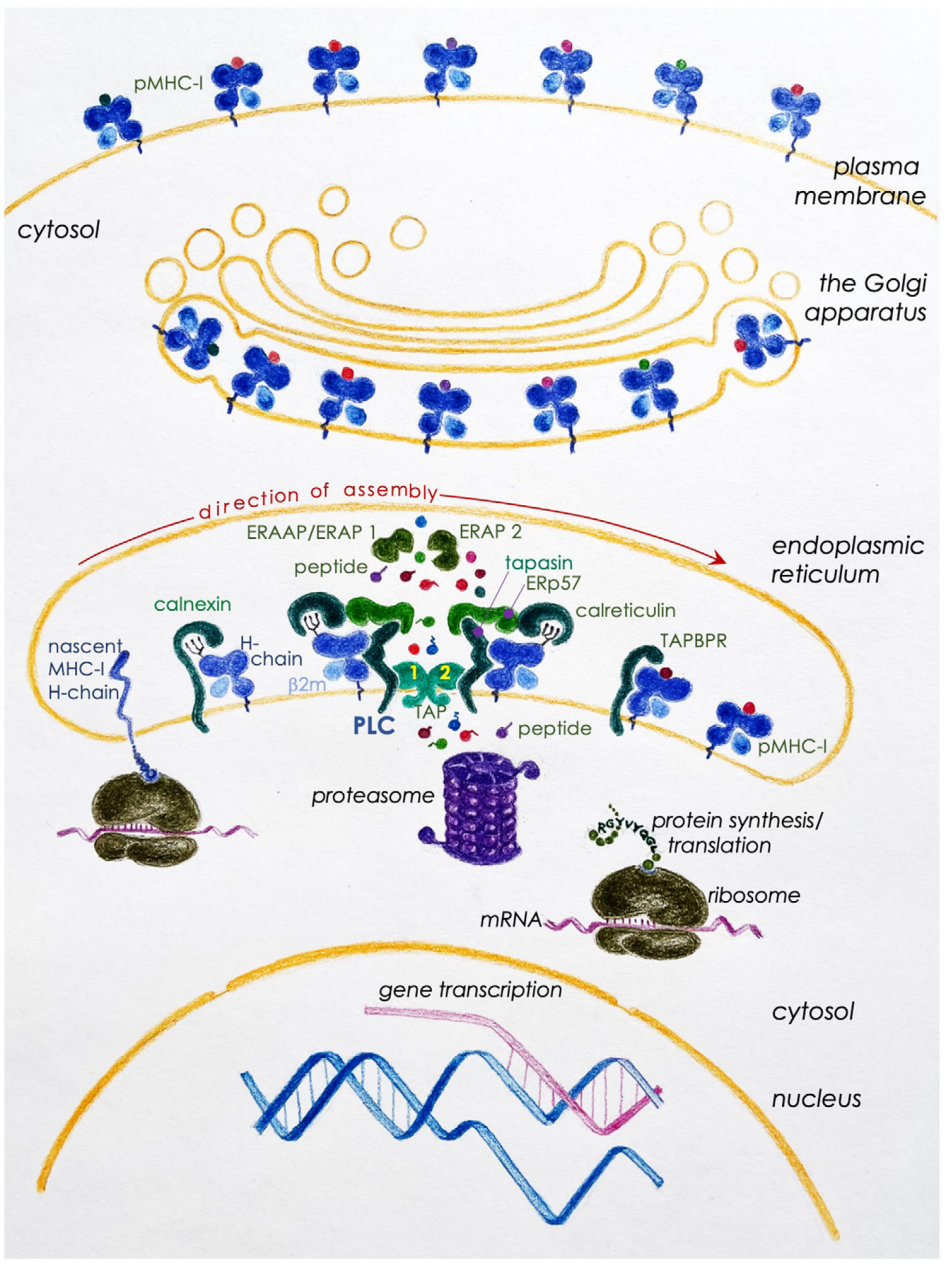
A schematic rendition of MHC‐I biosynthesis and assembly with peptide cargoes. The assembly of MHC‐I molecules begins with the co‐translational insertion of the heavy chain into the lumen of the endoplasmic reticulum (ER). Herein the nascent heavy chain binds to the ER chaperone calnexin to facilitate initial folding and assembly with β2‐microglobulin (β2m). This unstable heterodimer is stabilized by binding to a related ER chaperone calreticulin. This interaction makes the complex receptive to the peptide loading complex (PLC). This association with the PLC stabilizes the empty heterodimer such that the antigen‐binding groove adopts and maintains a conformation receptive to peptide loading. The PLC—consisting of the heavy chain‐β2m heterodimer, calreticulin, tapasin, and the ER‐resident thiol‐oxidoreductase/disulphide isomerase ERp57—facilitates peptide binding to the heterodimer. Initial peptide‐bound MHC‐I undergoes architectural editing via tapasin in the PLC to ensure high‐affinity peptide (p)/MHC‐I complex formation prior to exiting the ER. TAP‐binding protein related (TAPBPR), independent of the PLC, edits for high‐affinity peptide binding to MHC‐I in a poorly understood mechanism. Peptides generated in the cytosol—the sources of which and their production are explained in the text—are made available for pMHC‐I assembly in the ER lumen by transporter associated with antigen processing (TAP)‐1 and TAP‐2. Many of the peptides that are delivered into the ER are longer than the preferred 8–10 residues; these undergo further trimming by ER aminopeptidases, human ERAP1 (mouse ERAAP) and/or human ERAP2. Finally, high‐affinity pMHC‐I complexes are released from the PLC, which then falls apart into constituent parts, available for the next round of pMHC‐I assembly. Perhaps to make the process efficient, in addition to peptide translocation from the cytosol to the ER lumen, TAP‐1 and TAP‐2 heterodimer forms a scaffold that tethers two PLCs into a complex. pMHC‐I released from the PLC quickly egresses from the ER, and negotiates the Golgi apparatus en route to the cell surface for an appraisal by CD8^+^ T cells

The assembly of the MHC‐I molecule is schematised in figure 1. Assembly begins with the co‐translational insertion of the MHC‐I heavy‐chain into the ER. This heavy‐chain co‐translationally complexes with calnexin [122, 123, 124, 125]—an ER‐resident, lectin chaperone that assists the folding and assembly of newly synthesised proteins [126, 127, 128]—via a reducing monoglucosylated glycan. The heavy‐chain‐calnexin complex associates with β2m to form a transient, unstable heterodimer. This heterodimer formation is probably assisted by calnexin [125]. As heavy‐chain+β2m heterodimers form, calnexin dissociates to permit calreticulin—another ER‐resident, lectin chaperone [128]—to bind to this binary complex, again via the terminal glucose in the glycan. Calreticulin in this ternary structure recruits the thiol‐oxidoreductase/disulphide isomerase ERp57. This heavy‐chain+β2m+calreticulin+ ERp57 quaternary complex recruits tapasin and TAP (transporter associated with antigen processing, a heterodimer of TAP1 and TAP2) to form the peptide loading complex (PLC). Each PLC consists of a single heavy‐chain+β2m heterodimer, and each TAP tethers two PLCs as one tapasin molecule binds to each of the two TAPs in the functional heterodimer. PLCs await translocation of peptides from the cytosol into the ER lumen by TAP to facilitate the formation of a functional peptide (p)MHC‐I molecule. Upon binding the appropriate peptide, the fully assembled pMHC‐I dissociates from the PLC, egresses the ER, negotiates the Golgi apparatus, en route to the cell surface [121, 129, 130, 131].

### 5.3 Trimming to fit the groove

TAP heterodimers transport peptides from the cytosol to the lumen of the ER to overcome the topologic barrier between the compartments where cells generate peptides and the site where cells assemble MHC‐I molecules. TAP has a loose ligand specificity: it binds peptides that contain carboxy‐terminal hydrophobic or basic residues. Such carboxy‐termini are known to bind to MHC‐I molecules across all species. TAP transports peptides made up of 14–15 amino acid residues, therefore much longer than those that bind to MHC‐I [116, 118, 119, 120, 132, 133]. Long peptides are trimmed to size by ER‐associated aminopeptidase/s associated with antigen processing (human ERAP/mouse ERAAP). In a structural acrobatic, ERAP1 trims long peptides to the size that fit them into the antigen‐binding groove sometime destroying MHC‐I ligands [134, 135, 136, 137, 138, 139, 140, 141].

The importance of peptide trimming in the ER bore out in experiments in which peptides assembled with H‐2K^b^ and D^b^ molecules in ERAAP‐deficient and ‐sufficient cells were eluted and subjected to LC‐MS/MS analyses. While retaining a good fraction of peptides presented by MHC‐I of wild type ERAAP‐sufficient cells, ERAAP‐deficient cells, in addition, ferried ligands bound to mouse MHC‐I that had significantly altered its composition and length. Further, the latter peptide set was extended at the amino‐terminus and not at the other end. Consistent with these findings, wild type ERAAP‐sufficient mice elicited a strong CD8^+^ T cell response against ERAAP‐deficient spleen cells indicating that the self immunopeptidomes displayed by MHC‐I in ERAAP‐deficient cells were immunogenic [134]. Similar features were also reflected in mouse cytomegalovirus (CMV)‐derived peptides presented by cells devoid of functional ERAAP. What is more is that the self and CMV peptides presented by ERAAP‐deficient cells elicited a distinct CD8^+^ T cell response focused on the N‐terminal extension of the peptide [142].

Certain inflammatory diseases show linked association between ERAP1 and HLA‐I alleles (refs. [143, 144, 145] and reviewed in refs. [146, 147, 148]). The gene coding for ERAP1, more than ERAP2, is polymorphic (reviewed in refs. [146, 147, 148]). Amino acid altering differences map either directly to ERAP1's enzymatic site, substate‐binding site, or sites that can impact these activities (refs. [144, 149, 150, 151, 152, 153] and reviewed in refs. [146, 147, 148]). Hence, such polymorphisms could alter immunopeptidomes and form the basis of disease. Consistent with this notion, ankylosing spondylitis‐disposing HLA‐B*27 and the Behçet's disease‐associated HLA‐B*51:01 immunopeptidomes are significantly altered in the absence of functional ERAP1 [154, 155, 156]. Moreover, ERAAP deficiency in tumour cells appeared immunogenic, and abrogated a tumour in a mouse colorectal cancer model [157]. Together, these findings describe the profound effects ERAP/ERAAP has on the immunopeptidomes of healthy and diseased cells and reveal new targets to treat human diseases.

Human and mouse MHC consists of several clusters of multi‐gene families that encode proteins that control both the innate and adaptive immune responses. The MHC‐I molecules described thus far are products of MHC‐Ia cluster, which consists of genes that are highly polymorphic. In contract to these, the MHC‐Ib cluster consists of numerous genes that are highly conserved even across species. Genes in this cluster were once considered evolutionary vestiges but are now known to encode molecules that control both T cell and natural killer cell functions—for example, the human HLA‐E and the orthologous mouse H‐2Qa1, which are ligands of activating CD94/NKG2 heterodimeric receptors [158, 159, 160]. To begin to understand the immunopeptidomes of MHC‐Ib and their biology, peptides were eluted from the surface of ERAAP‐sufficient and ‐deficient cells, and their features determined in high‐throughput mass spectrometry experiments. Peptidomes associated with MHC‐Ia molecules have features described above. Curiously, the number and immunogenicity of peptidomes presented by MHC‐Ib molecules were substantially increased in ERAAP‐deficient cells [161, 162]. Hence, ERAAP trims a substantial repertoire of peptides to fit into MHC‐Ib grooves. These findings convincingly implicate the ER as a major site for MHC‐I associated immunopeptidome generation, shifting from the conventional notion that most MHC‐I associated peptides are generated in the cytoplasm by the action of the proteasomes—more on this matter is below.

Several studies have found that components of the MHC‐I restricted antigen processing pathway also impact MHC‐II antigen presentation. One such study reported that ERAAP‐deficiency altered the immunogenicity of certain cytosolic peptides presented by H‐2A^b^ molecules ([163] and references therein). Mass spectrometry analyses of peptidomes found that H‐2A^b^ molecules presented a pool of peptides derived from the cytosol of ERAAP‐deficient cells [164]. Hence, ERAAP has effects on MHC‐II associated peptidomes as well; how this occurs remains and awaits investigation.

### 5.4 Editing for best fit

Tapasin and its homologue TAP‐binding protein related (TAPBPR) function to facilitate peptide binding to assembling MHC‐I molecules and also as editors, the former in the PLC and the latter independent of the PLC. Current evidence suggests that tapasin and TAPBPR quality control the C‐terminal end of the peptide. This editing function ensures that peptides of sufficient affinity are loaded into the antigen‐binding groove to assure stable display of pMHC‐I at the cell surface [165, 166, 167, 168, 169, 170]. This editing function of both tapasin and TAPBPR loads soluble MHC‐I molecules with high affinity peptides, a process capitalised to generate high affinity pHLA‐I tetramers by in vitro catalysis using TAPBPR [99, 171]. Despite these very close functional similarities, TAPBPR does not compensate for the function of tapasin in tapasin‐deficient cells perhaps because the former functions independent of the PLC and the latter within it. Curiously, neither are obligatory chaperones in the assembly of MHC‐I molecules [172, 173, 174]. Hence, HLA‐I (human leukocyte antigen class I)—encoded by the highly polymorphic human *MHC‐I* genes—allelic variants have varying dependencies on tapasin for proper assembly with peptides [172, 173, 174]. Despite varying dependencies, HLA‐I molecules assemble with high affinity peptides, even when their dependency on tapasin is low, into stable, functional molecules and maintain control of CD8^+^ T cell responses [174]. Consistent with that finding, a very large‐scale study of over 90 HLA‐I molecules showed no differences in the composition of immunopeptidomes displayed by human MHC‐I molecules which assemble dependent versus independent of tapasin [49]. Evolution's purpose for the homologues to behave differently, and for varying tapasin dependency in HLA‐I assembly remains elusive! Studies of immunopeptidomes in tapasin and TAPBPR doubly deficient cells and mice could illuminate both the biology and evolution of varying tapasin dependency.

## 6 NEW MOLECULAR CELL BIOLOGY AND THE SOURCES OF PEPTIDES

### 6.1 Cellular roteostasis: Roles for proteasomes, immunoproteasomes, & thymoproteosomes

It is generally thought that the natural turnover of proteins in the cytoplasm contributes a sizable fraction of peptides to the immunopeptidome. This assumption, however, is at odds with four features of peptides presented by MHC‐I molecules: First, MHC‐I immunopeptidomes contain peptides derived from long‐lived proteins whose half‐life average ∼45 h [175, 176]. Second, presentation of virus‐derived peptides occur even before virus proteins are detectable and assembly begins, and excess proteins turn over: for example, VSV‐N (vesicular stomatitis virus nucleocapsid), VACV (vaccinia virus), and IAV (influenza A virus) [177, 178, 179, 180, 181]. Third, low copy number proteins—those that form a minor fraction of a given cell's proteome—are peptide sources and compete favourably against highly represented cellular proteins, which includes supra‐stoichiometrically generated proteins that either misfold or cannot find partners in multimeric proteins [182, 183, 184]. Fourth, although controversial, peptides are derived from genome hotspots [57, 58], which is not observed in tumour cell lines [49, 164] suggestive of transcriptional dysregulation in cancers. Here we begin with the contributions of the proteasomes in sculpting the immunopeptidome in relation to other cellular mechanisms.

#### 6.1.1 Proteolysis

Proteasomes are multicatalytic endoproteinase complexes composed of four rings in which each ring is made of seven related subunits. The two outer rings, composed of α subunits, sandwich the two inner catalytic rings of β subunits. This quartet of heptameric rings, forming the core 20S proteasome, assembles in such a way that they form an interior chamber. The N‐terminal residues of the α rings gate the catalytic rings, the opening of which is controlled by the regulatory cap made up of the 11S proteasome activators (PA) and/or the AAA+ ATPase‐containing 19S unit. The N‐terminus of β1, β2 and β5 subunits is exposed to the interior chamber and contains the proteolytic active sites (reviewed in [185, 186, 187]).

IFN‐γ enhances MHC‐restricted antigen presentation by inducing the expression of multiple structural and regulatory genes, including HLA‐I, β1i/LMP (Low Molecular mass Polypeptide)‐2, β2i/MECL‐1 (Multicatalytic Endopeptidase Complex‐Like‐1), β5i/LMP7, the regulatory cap PA28 and ERAP, amongst others, especially within immune cells in healthy individuals. The induced proteasomal components occupy the place of the homologous component within the constitutive, standard proteasome, creating the immunoproteasome. Immunoproteasome formation is a highly ordered process: β2i requires β1i for efficient incorporation into preproteasomes, and preproteasomes containing β1i and β2i require pre‐β5i for efficient maturation and, thereby, ensures the assembly of homogeneous immunoproteasomes for efficient generation of peptides presented by class I molecules [185, 188, 189, 190].

Virus infection and tumour microenvironments induce type I IFN, IFN‐γ, and tumour necrosis factor α (TNF‐α) production, which in turn promotes the induction of immunoproteasomes and components of the PLC. Consistent with this finding, mass spectrometry‐assisted proteomics experiments showed subtle to substantial shifts in the self immunopeptidomes after microbial infection, for example, HIV‐1 (human immunodeficiency virus), IAV, measles virus, VACV, and *Toxoplasma gondii* or within cancer cells [47, 96, 164, 191, 192, 193, 194, 195, 196]. Whilst the immunologic consequences of the immunopeptidome shift in response to virus infection remain to be determined, IFN‐γ did not influence the immunopeptidomes in another study [49]. In striking contrast, a high‐throughput immunopeptidome analysis of non‐transfected primary cells—thymocytes and professional antigen presenting DCs—of wild type and mouse β2i/MEKL‐ and β5i/LMP7‐deficient mice showed a significant contribution of immunoproteasome in sculpting the immunopeptidome. This study showed that the immunoproteasome has proclivities for cleavage site, not amino acid residue, and unstructured regions in the substrate proteins [197]. Similar changes to cancer immunopeptidomes as a consequence of IFN‐γ and TNF‐α action were observed in other studies and, as a consequence, impacted tumour immunity or cancer immunotherapy [194, 195, 196]. Furthermore, there are reports that show that the immunoproteasomes can make or break epitopes with significant immunologic consequences [194, 198, 199, 200, 201, 202]. So then, why the differences between these findings? One cause may be the use of different cell sources, tumour lines versus primary cells; two, use of IFN‐γ‐induced cell lines versus immunoproteasome‐deficient primary cells; three, study of immunopeptidomes associated with soluble versus membrane‐anchored MHC‐I molecules; and four, primary cells are but tumour cells transfected with monoallelic HLA‐I transgene are not under the control of IFN‐γ [47, 49, 96, 164, 191, 192, 193, 197, 203]. Alternatively, it is possible that the generation of the large majority of peptides in the immunopeptidome may not require the constitutive or IFN‐γ‐induced proteasomes. This notion is consistent with the finding that cells continue to present peptides even when proteasome functions are inhibited by lactacystin, epoxomicin, or bortezomib (velcade) [204, 205, 206, 207, 208, 209, 210]. A resolution to the different outcomes may be reached by a comparative immunopeptidome study of uninduced and type I IFN‐ or IFN‐γ‐induced primary DCs, which—with some limitations associated with in vitro cultivated bone marrow‐derived DCs—may closely reflect that which might occur in vivo.

#### 6.1.2 Peptide dicing and splicing adds to antigen diversity

An effort to identify the peptide epitope recognised by a HLA‐A3‐restricted renal cell carcinoma‐specific CD8^+^ T cell clone led to a serendipitous finding that the epitope was generated by the splicing of the protein antigen FGF‐5 (fibroblast growth factor‐5) [211]. Whilst the evidence pointed to the cytosol as the site of protein splicing, shortly thereafter, the proteasome was shown to splice peptide epitopes together after proteolytic cleavage within its catalytic chamber. This notion was firmed by incubation of purified 20S proteasomes with the precursor peptide RTKAWNRQLYPEW derived from gp100/MEL melanocyte antigen and identification of one of the spliced products RTK—QLYPEW by mass spectrometry and T cell assay [212]. Additional spliced virus‐ and tumor‐derived antigenic epitopes are known [213, 214]. Such diced and spliced epitopes derived also from a minor histocompatibility (H) antigen, that which mediate graft‐versus‐host response in HLA‐identical bone marrow transplant recipients. In the case of the minor H antigen SP110, cleavage between the threonine‐alanine peptidyl‐bond (underlined) within the **STPK**RRHKKK**SLPRG** **T**ASSR (bold indicate the two parts of the spliced epitope) fragment yielded the necessary energy for re‐ligation of two resulting fragments in reverse order to create the HLA‐A*03:01‐restricted, SP110‐derived minor H epitope SLPRGT—STPK [215]. Spliced peptides are not peculiar to virus‐, alloantigen‐ or cancer‐derived epitopes but are derived from bacterial proteins as well, for example, *Listeria monocytogenes—*a bacterium with an obligatory cytosolic lifestyle [216, 217]. Thus, a novel antigen processing mechanism involving cleavage and re‐ligation of peptide fragments within the proteasome was revealed. It is noteworthy that up until these discoveries, protein splicing was known only in plants (e.g., concanvalin A) and in unicellular organisms, including eukaryotes. Hence, peptide splicing in the proteasome for antigen presentation unveils a new molecular cell biology of metazoans.

As several such examples followed (reviewed in [218, 219]), three questions became apparent: one, does splicing occur only in *cis*—between peptides generated from the same protein as in the three examples above—or can it occur in *trans*—between peptides generated from two different proteins; two, what fraction of the peptides in a immunopeptidome owes to peptide splicing; and three, can cytokines influence peptide splicing in the proteasomes considering that IFN‐γ induces immunoproteasomes? Answers to these questions have come from deep sequencing of the immunopeptidomes eluted from HLA‐I molecules. Current evidence suggests that both *cis*‐ and *trans*‐splicing generate HLA‐I associated immunopeptidomes. And that, albeit controversial [49, 220, 221], peptide splicing may contribute between 1—30% of peptides within the immunopeptidome [49, 213, 220, 222, 223, 224, 225, 226].

The large range in the contribution of spliced peptides to the immunopeptidome reported from different works can be rationalised by understanding the methodology applied to their discovery [227]. LC‐MS peptide identifications are generally made by assigning the most probable amino‐acid sequence from a sequence database to a given spectrum, and the accuracy of this assignment is dependent on many factors including the spectral quality and the size and design of the sequence database. While the accuracy of such peptide‐spectrum to sequence assignments may be controlled through parallel interrogation of randomised sequence databases for estimation of the false discovery rate, the designation of a specific amino acid sequence to be a product of proteasomal splicing needs careful biological validation [213, 226, 228]. Since validation of spliced peptide assignments can and has to date only been performed for subsets of peptide annotations, the true extend of spliced peptide sequences in the immunopeptidome remains as yet undetermined [227].

β5i/LMP7‐containing immunoproteasome enhance the production of a novel gp100/MEL epitope by peptide splicing: RTKAWNRQLYPEW substrate reverse spliced to QLYPEW—RTKAWNR and diced to QLYPEW—RTK product epitope [229]. Similarly, immunoproteasome was shown to enhance the production of the SP110‐derived minor H epitopes as well [215, 218]. Curiously however, in large‐scale studies, IFN‐γ had little influence if any on the nature of the peptides in the immunopeptidomes investigated even though the cytokine induced components of the immunoproteasomes and accessory protein in the PLC [49].

Then there are thymoproteasomes, those made with the β5t subunit, which assembles in cortical thymic epithelial cells in association with β1i and β2i. β5t assumes the place of β5 and β5i in these cells [230]. β5t‐containing thymoproteasomes, are thought to promote positive selection of CD8^+^ T cells, but the underlying mechanism remains unknown [230, 231, 232, 233, 234]. The chymotrypsin‐like proteolytic activity of thymoproteasomes is low and, consequently, produce a distinct immunopeptidome [231, 232]. Or alternatively, as β5t, β5 and β5i are paralogues begotten from gene duplication (β5t and β5) and two rounds of whole genome duplications (β5 and β5i) [235, 236], and because β5i enhances splicing of certain peptide epitopes [215, 218, 229], an intriguing possibility is that the thymoproteasomes may have increased peptide splicing activity. These predictions, however, require further investigation.

## 7 DEFECTIVE RIBOSOMAL PRODUCTS

The immunodominant CD8^+^ T cell epitope from VSV‐N is generated within the first 45 min post infection of cells [177]. A similar observation was reported for the HIV‐1 Gag protein, which is an incredibly stable protein [237]. Hence, the presentation of these epitopes occurs much sooner than the turnover of the two source proteins begins. As well, over 30% of new synthesised proteins are turned over by the proteasomes [237]. This rapid protein turnover is consistent with the finding that the major substrates for TAP transport are generated from newly translated proteins [238]. These astute observations led Yewdell to postulate the DRiP hypothesis over 20 some years ago [239]. A kinetic study of antigen presentation by DC‐like DC2.4 cell line provided compelling evidence for the DRiP hypothesis: Focused on eight of the 49 epitopes recognised by VACV immune CD8^+^ T cells, these peptides were quantified over a 12.5‐h infection period in a highly sensitive MRM (multiple reaction monitoring) mass spectrometry experiment. These eight epitopes were chosen because, (a) their source ORFs were previously shown to be immediate early, early and late genes; and (b) study of multiple epitopes in a single experiment provides a better picture of peptide processing and presentation than a single epitope at a time. Alongside epitope quantification, the expression of source ORFs were quantified over time. Such an in‐depth analysis of multiple epitopes was possible because of the high sensitivity and accuracy of the mass spectrometer used in this study. The kinetic analysis of the two parameters demonstrated that epitope presentation begins even before source protein levels plateau in DC2.4 cells. Furthermore, epitope abundance was not correlated with source protein abundance neither was immunodominance [179].

Estimates are that DRiPs contribute to >30% of the peptides in the immunopeptidomes [239]. It is noted that the DRiP hypothesis does not in any way refute the contribution of peptides emerging from the natural turnover of stable cellular proteins—proteins that retire from their function/s—to the immunopeptidome [240]. In the light of evolution, it makes perfect sense to generate and present microbial antigens at early stages of infection as discovered in the kinetic study above so as to achieve effective immune surveillance and to stymie an impending disease.

So, what are the sources of DRiPs? This search for DRiPs turned up some exciting new molecular cell biology! DRiPs are largely formed from unstable polypeptides because they are (**a**) translation products of stalled ribosomes; (**b**) misfolded proteins; (**c**) those that did not find their partner/s in heteromeric complexes; or (**d**) products of mutant genes, many of which arise to set off tumorigenesis. Finally, SLIPs—short lived proteins—are another source of peptides for the immunopeptidome. SLIPs are a category of proteins that retired even before they were fully formed to execute their function/s.

### 7.1 Unconventional translation: Where shall I begin?

Few groups had reported MHC‐I restricted presentation of cryptic peptides—peptides that arise from polypeptides templated from the 5’ and 3’ untranslated region (UTRs,) and alternative reading frames (ARFs) that are generally thought not to be translated. Such cryptic peptides form targets for virus‐ and tumor‐specific T cell‐mediated immunosurveillance ([241, 242, 243] and reviewed in ref. [244]). It is estimated that the cancer immunopeptidomes are constituted by 2%–20% cryptic peptides [242, 245].

In studies designed to understand how cryptic epitopes arise, Shastri and colleagues discovered the surprising use of CUG in contrast to the conventional use of AUG as the initiator codon [246, 247, 248, 249, 250]. What is more is that translation initiation at CUG used the elongator leucinyl‐tRNA anti‐codon Watson‐Crick base paired with the leucine codon. That is, the methionyl‐initiator tRNA (tRNA_i_^Met^) is not used as the initiator tRNA in a wobbled base pairing with the CUG codon. Initiation at CUG required eIF2A (eukaryotic initiation factor‐2A) to form the ternary complex [249]. This form of unconventional translation is enhanced by proinflammatory signals including virus infection [180, 251] and appears to guide tumorigenesis, which upends conventional translation by the phosphorylation of eIF2α [252]. New evidence indicates that RPS28 (40S ribosomal protein S28) tunes peptide generation via unconventional translation [253]. These findings alert to immunoribosomes, those potentially dedicated to creating self and non‐self immunopeptidome. More on this matter is below.

Under homeostatic conditions, conventional translation requires the recruitment of the ternary complex—composed of eIF2 (made up of α, β, and γ heterotrimer)‐GTP and tRNA_i_^Met^—into a complex consisting of the small ribosome subunit and multiple eIFs (eIF1, ‐1A, ‐2, ‐3, and ‐5) to form the 43S preinitiation complex (PIC). This PIC becomes receptive to the translation‐primed mRNA, which is composed of 5’GpppA cap‐bound eIF4E, eIF4G, eIF4A, and eIF4B, and when bound together forms the 48S PIC (see [254]). Whilst protein synthesis starts predominantly at the initiator AUG codon flanked by the consensus Kozak sequence […(‐3)A/G‐NN‐AUG‐G(+4)…], some translation occurs at the upstream AUG codons within 5’‐UTRs (untranslated region containing an upstream open reading frame or uORF) as well. In general, uORF‐encoded polypeptides are short, composed of ∼10 amino acid residues. This form of unconventional translation is enhanced by cellular stress caused by tumorigenesis, infection or nutrient starvation via phosphorylation of eIF2α and formation of an alternative ternary complex composed of eIF2A‐GTP, which replaces eIF2‐GTP. The alternative ternary complex utilised non‐AUG codons, such as CUG>GUG>AUG>>UUG for translation [252]. Together then, unconventional translation reduces conventional protein synthesis but biases the process toward cancer specific gene expression. Coupled with alternative initiator codon usage, the immune system has found a way for immunosurveillance so that tumours and infected cells have nowhere to hide.

Hence, translation from 5’‐ and 3’‐UTRs (see refs. in the preceding paragraph), ARFs [243, 255, 256] including the negative strand (e.g., influenza virus) and translation initiation from a non‐AUG but specifically CUG codon are all known to contribute to immunopeptidomes (reviewed in refs. [239, 246, 257]).

An estimated 1% of the proteome mis incorporates methionine residues with the use of Met‐misacylated onto non‐methionyl‐tRNAs. Methionine misincorporation into the proteome not only protects proteins from oxidation, but also expands the functional, expressed genome. As viruses, dead and live, enhance Met‐misacylation via innate signalling mechanism and reactive oxygen production, such Met‐misacyalted proteomes can contribute peptides with non‐templated methionine/s to immunopeptidomes [258]. This notion awaits formal evidence.

### 7.2 W‐bumps stall ribosomes to frameshift translation

One mechanism of tumour immune evasion involves the induction of indolamine‐2,3‐dioxigenase‐1 (IDO‐1) by IFN‐γ (reviewed in ref. [259]). Nonetheless, inhibition of IDO‐1 in conjunction with PD‐1 blockade in clinical trials did not enhance the efficacy of checkpoint blockade alone (reviewed in ref. [260]). A mechanistic study of this unanticipated clinical outcome revealed yet another new molecular biology of the gene and a potential mechanism of immune surveillance against cancers: Under chronic conditions of IFN‐γ stimulated IDO‐1 activity, the tested melanoma cells deplete tryptophan via the kynurenine metabolic pathway. Consequently, ribosomes in these cancer cells accumulate after the tryptophan codon, causing what the authors call a ‘W‐bump’. W‐bumps result in translational frameshifts and the generation of altered polypeptides which contribute to HLA‐I associated tumour‐specific immunopeptidomes. What is more is that some of the altered peptides elicit CD8^+^ T cell response [261]. Hence, IFN‐γ‐induced IDO‐1 production may play a significant role in immune surveillance against tumours. Inhibition of IDO‐1 then prevents the generation of neoepitopes and, thereby, obviates antitumor immunity.

### 7.3 Nuclear translation: Translating introns and across intron‐exon boundaries

Translation of introns and intron‐exon junctions provide a source of DRiPs [244, 262, 263, 264, 265, 266]. Two studies provide compelling evidence that antigenic peptides are generated via pioneer translation in the nucleus. In the first of these, inhibitors of RNA polymerase II (pol II) that prevented nuclear export of transcribed mRNA blocked cytoplasmic translation of a recombinant IAV neuraminidase (rNA) gene. This recombinant protein generated an antigenic peptide engineered into rNA stalk region despite undetectable cytoplasmic translation [267]. The second study used a model in which mRNA is super rapidly exported from the nucleus in a HIV‐1 Rev‐dependent CRM1‐mediated pathway. Super rapid mRNA export decreased the presentation of an antigenic peptide whose gene was engineered into the intron of the β‐globin gene consistent with the nuclear translation of antigenic epitopes. Further, in situ localization mapped the pioneer translation product to peri‐nuclear area in association with RPS6 and RPL7 [264]. So then, are there immunoribosomes?

### 7.4 Immunoribosomes—Gained in translation

DRiP hypothesis had postulated the presence of immunoribosomes as a means to channelize protein synthesis to peptide generation and TAP transport [268] (reviewed in refs. [269, 270, 271]). Initial evidence for the engagement of a distinct ribosome subset in translating DRiPs came from studies that inserted a pretermination codon downstream of a segment that encodes an antigenic peptide from within the β‐globin gene. This premature stop codon initiates the RNA quality control mechanism termed non‐sense mediated decay (NMD; see ref. [254]). NMD prevented mature β‐globin production yet produced the antigenic peptide via pioneer round of translation. The generation of the antigenic peptide required eIF4G but not eIF4E [265]—the cap‐binding protein essential for mRNA to bind to other eIF4s and, thereby, to the 43S PIC to form the 48S PIC (see ref. [254]).

Direct evidence for a role for immunoribosomes in generating immunopeptidomes emerged from painstaking CRISPR/Cas9 screen of cells targeting each of the 80 ribosomal protein genes, one‐at‐a‐time [253]. The presentation of the ovalbumin peptide from influenza A virus gene expression detectable by SIINFEKL/H‐2K^b^ complex‐reactive monoclonal antibody binding was the readout. This screen discovered that 67 of the 80 RPs were essential for cell viability. Of the remaining 13 RPs (ribosomal proteins), one 60S protein RPL6 deficiency decreased SIINFEKL peptide generation, while RPL28 deficiency increased it. Deficiencies in the two RPLs had no effect on the transcripomes. Mechanistically, RPL6 controls ubiquitin‐dependent proteasomal destruction of DRiPs which explains the increase in SIINFEKL peptide generation in RPL6 deficient cells. RPL28 on the other hand controls ribosomal RNA methylation, and with translation factors channel translation products to the ER translocon and/or TAP. Hence, the two 60S large subunit proteins inversely control DRiPs [253].

Deficiency in a third RP, RPS28, increased HLA‐A2 levels at the cell surface; one plausible explanation for this increased expression could be increased peptide supply. Ribosomal profiling (Ribo‐Seq) experiment showed increased unconventional translation of uORFs from 5’ and 3’ UTRs from non‐AUG initiator codon. Of immunologic consequence, the increased peptide supply gained from RPS28 deficiency engineered into a melanoma cell line made the tumour line much more sensitive to NY‐ESO derived pHLA‐A2‐specific CD8^+^ T cell‐mediated killing [253]. These data together, for the first time, suggest the existence of immunoribosomes, which may play an important role in cancer immunosurveillance. Hence, the authors conclude that mutations in RP genes, which are common in cancers [272], may result in cancer immunoevasion.

In sum, unconventional translation, ‘W‐bumps’ and translational stall and frameshift, translation of intron and intron‐exon boundaries, and translation with immunoribosomes expand the sources of DRiPs and, consequently diversify the self and non‐self immunopeptidomes. Such DRiPs can be easily missed in experiments that use the current proteogenomics methods. To overcome this limitation, proteomics approaches need to incorporate Ribo‐Seq technologies [252, 273, 274] to characterize a homeostatic and cancer translatome so as to better define what immune self and non‐self mean to T cells. New studies are beginning to address this need in the cancer immunopeptidome space [242, 275, 276].

## 8 TAKING ANTIGEN PRESENTATION TO THE BAZAAR

This subtitle paraphrases the title of Professor Jan Klein's Plenary Talk at the 6th International Congress of Immunology held in Toronto, Canada, June 1986. Whilst, in 1986, it seemed to Professor Klein such optimism too early, therapies based on harnessing antigen presentation by means of vaccination, as well as T cell expansion and cell therapy are 20th century advances reduced to clinical practice in the advancing 21st century.

### 8.1 Microbial epitope discovery

The different approaches to discover T cell epitopes have been reviewed recently [277, 278] and, hence, not belaboured here. The most popular of these is algorithm‐based epitope prediction coupled with biochemical and immunologic validation. Over 40 such algorithms exist, which have been recently compared and reviewed by others [279]. Further, algorithms trained on naturally processed immunopeptidomes in addition to the traditional affinity‐based tools have better predictive power as has the recent study of 95 HLA‐I immunopeptidomes consisting of over 185 thousand peptides [49]. Two other methods gaining interest in T cell epitope discovery include, one, a massive, high‐density peptide array technology that allows identification of all possible peptides that have the potential to bind to and be presented by MHC molecules in the absence of a functional PLC [280, 281]. And two, phage display of pMHC complexes and epitope identification with yeast display of TCRs [282].

Algorithm based epitope discovery could lead to the discovery of mimotopes because it focuses mainly on MHC binding and antigen processing and presentation but does not account for the features for antigen‐receptor interactions [283]. Accounting for this interaction is critical as the TCR is very sensitive: that is, the receptor can recognize and respond to one‐to‐ten molecules of an antigen [84, 284]. As well, it can discriminate between two peptides differing by a methylene group or a methyl and a hydroxyl group in an accessory anchor—for example, H4 minor histocompatibility alloantigens [86, 285, 286]. This sensitivity coupled with a rather loose ’recognition logic‘ and micro‐to‐milli‐molar binding affinity with which the TCR interfaces its cognate antigen—the p/MHC—is thought to make the TCR highly cross reactive [286 ^‐^ 290]. A case in point is the recognition of ∼100 different peptides by an H4^b^‐reactive CD8^+^ T cell line [291]—yet the 100 mimotopes so identified did not contain the actual epitope [86, 285]. This was not a peculiarity of an alloreactive TCR because the simian virus 40‐derived epitope‐4 specific and herpes simplex virus 1 glycoprotein B‐reactive T cell clones also recognised over 50 mimotopes. A common feature within the three mimotope sets was the presence of a TCR‐specific recognition motif consisting of one or two conserved putative solvent exposed residues with a potential to interact with the TCR. At the other extreme, a single autoimmune TCR was recently shown to recognize over a million different peptides within a broad cross‐reactivity profile [292]. Such cross reactivity is not peculiar to MHCI‐restricted TCRs as several MHCII‐restricted TCRs were shown to cross react in a similar manner (see refs. [282, 293] and references therein). The cross‐reactive feature of the TCR further underscores the critical need for comprehensive immunologic validation of an identified epitope. Furthermore, inclusion of structural features of pMHC as well as TCR‐pMHC binding interactions (e.g., refs. [287, 288, 289, 294] and references therein) into newer iterations of algorithms can enhance their predictive power [282]. These learnt adaptations to epitope prediction algorithms has significantly enhanced T cell epitope discovery [283, 295, 296, 297, 298].

Epitope prediction is high‐throughput and effective for microbes with small proteomes such as those of viruses, the largest of which express ∼250—300 open reading frames (ORFs). Experiments using the power and rapidity of predictive algorithms coupled with T cell‐based validation have resulted in the discovery of numerous putative and actual immune epitopes that are deposited in the IEDB (immune epitope database) [299]. In contrast, discovery of T cell epitopes from larger microbes such as *M tuberculosis* and *Plasmodium* spp. by using prediction algorithms would be challenging because the expressed genome of these microbes can encode ∼4000—6000 proteins. In addition to the scale (about a million potential determinants) of epitope screening problem, these microbes might use their own proteasomes to destroy beneficial epitopes discovered by predictive methods even before they are available for presentation by MHC‐I. Consistent with this notion, only a few epitopes were presented by HLA‐A2.1 molecules expressed by *M. tuberculosis* strain H37Ra‐infected U937‐A2 cells (3 nested/overlapping, HLA‐A*02:01‐resticted epitopes) [300] or *M. bovis*‐derived strain BCG‐infect THP‐1 cells (12 A*02:01‐resticted peptides) [301]. The large differences in the range of epitopes presented perhaps lie in the fact that viruses translate their ORFs and some of their ARFs on host ribosomes, DRiPs generated from which are substantial sources of antigenic peptides [255, 257]. By contrast, mycobacteria translate their genomes on their own ribosomes, wherein DRiPs may be lost to rapid degradation by microbial proteasomes [302, 303] and, hence, unavailable for presentation.

In striking contrast to the relatively small number of HLA‐A*02:01‐resticted mycobacterial peptides identified, a tedious and thorough study identified a large number of *T. gondii*—the agency of toxoplasmosis—encoded peptides (195) presented by A*02:01 on infected cells [47]. These peptides had several uncommon features: such peptides (a) were largely derived from the parasite's cytosol perhaps through direct delivery of the ligands from the parasitophorous vacuole to the host ER; (b) were longer than the conventional 8–10 mer peptides typically presented by MHC‐I molecules; (c) maintained the N‐terminal core binding motif as observed in canonical A*02:01‐bound ligands; and (d) were extended at the C‐terminus by 1–30 amino acid residues as gleaned from the pHLA‐I crystallographic structure [47]. If indeed the *T. gondii*‐encoded cytosolic peptides were delivered directly via a conduit between the parasitophorous vacuole and the ER, such peptides should be TAP‐independent—a notion that is easily tested. Whether features observed in *T. gondii*‐encoded ligands are unique to this pathogen or is common to microbes and parasites contained within parasitophorous vacuoles awaits further study. In this context, the features of HLA‐I restricted *T. gondii*‐derived peptides and other epitopes described earlier [304, 305, 306] bring perspective two orphan studies reported over 25 years ago describing the association of peptides longer than the conventional 8–10 mer to a mouse and a human MHC‐I molecule [307, 308, 309].

Naturally processed epitopes presented by several HLA‐I molecules have been characterised with the aid of proteomics approaches [45, 46, 47, 96, 100, 101, 164, 179, 191, 192, 193, 301]. A theme that emerged from one of these studies is that VACV‐infected cells generate many, many more epitopes than are antigenic—those recognised by CD8^+^ T cells that are elicited by virus infection . These antigenic epitopes were also immunogenic—that is, they elicited a CD8^+^ T cell response in the appropriate mouse strain. Whether the non‐antigenic virus‐derived peptides were immunogenic (for a definition of antigen and immunogen, see BOX 1) was not determined. Another study of VACV‐derived epitopes by mass spectrometry revealed that the mouse MHC‐I presents peptides derived from almost all 200 or so virus ORFs. Further a large majority of these peptides were immunogenic, suggesting that the mouse has a large T cell repertoire directed against VACV peptides. Whether all of these peptides are antigenic was not determined [46]. It is less likely that all of the naturally processed VACV peptides identified by mass spectrometry are antigenic because previous reports by this group showed that 49 VACV peptides accounted for all of the antigenic epitopes. Further, CD8^+^ T cell responses to five peptides accounted for up to 40% and to all 49 peptides accounted for up to ∼95% of the total response to VACV in mice [310, 311].

At first pass, it might seem that the infected cell wastes immense resources to generate and present so many different epitopes. But consider the following: if all of the readers of this manuscript are HLA‐B*07:02 positive, but express different HLA‐I molecules from the remainder five loci, our T cell repertoire would be as distinct and diverse as the number of individuals in the reader population. Hence, each repertoire will recognize a distinct, and potentially an overlapping set of epitopes. This is exactly what was observed in multiple studies [45, 312, 313], which we called variegated T cell antigen recognition [45, 314]. This variegated recognition coupled with heterotypic immunity perchance explains the success of vaccination with VACV against smallpox with the eventual eradication of the disease from the globe [101, 310].

### 8.2 Proteogenomics for cancer antigen discovery

Preceding the advent of the proteogenomic approach, tumour‐specific antigens were discovered with mass spectrometry of T cell active fractions [36, 37, 38]. When coupled with the idea that tumorigenesis involves tumour‐specific signalling events guided by phosphorylation‐dephosphorylation cascades, that approach led to the discovery of phosphopeptide epitopes uniquely expressed by cancer cells [39, 41, 42, 43]. This discovery has led to phosphoepitope‐based immunotherapy against cancers [40, 44]. More recently, several groups concurrently reported a proteogenomic approach that allows T cell epitope discovery from species with large proteomes such as ours and mice. This approach has led to the discovery of several cancer‐specific as well as minor histocompatibility alloantigen‐derived CD8^+^ T cell epitopes [57, 102, 103, 104, 105].

Proteogenomic approaches generally entail the refinement of the protein database used to interrogate obtained MS fragment spectra, which can be achieved by integrating whole genome, exome, or RNA sequencing information; the latter can be further refined by ibo‐seq approaches. Definition of non‐synonymous single nucleotide polymorphisms (nsSNP) is most frequently defined in relationship to the same individual's non‐cancerous genome (whole exome sequencing data) or (less frequently) transcriptome (RNA sequencing data). Novel de novo assembly tools further allow the reconstruction of the transcriptomic landscapes of cells from RNA sequencing or ribosome sequence profiling data, including transcripts from unconventional sources, in order to assist their discovery in the immunopeptidome: that is, retained intronic sequences, lncRNAs, antisense transcripts, human endogenous retroviral‐derived reterotransposable elements, and unannotated gene products [242, 243, 275, 276, 315, 316, 317, 318].

There are two strategies to integrate these data with immunopeptidomic analyses. Firstly, the translated mutant proteome is subjected to T cell epitope prediction using HLA‐binding predictors: for example, NetMHCpan4.1 [319]. This information then allows the specific targeted acquisition of the predicted variant peptide sequence within the material eluted from a given MHC‐I molecule using MRM experiment. From the resulting naturally processed tumor epitopes, immunogenicity was predicted in silico with both immunogenicity and protection validated in vivo [102, 103, 105]. Alternatively, the genomics information can be included in the protein databases used for interrogation of the LC‐MS spectra from purified MHC‐associated peptidomes. Here, dependent on the quality and extent of the genomically refined protein sequence data, this approach allows for the discovery of non‐canonical antigens in the context of disease as exemplified above.

The potential variation within each peptide that is caused by snSNPs is ascertained from the genomes or transcriptomes of allogeneic or cancer cells and validated in immunologic assays [57, 104], and immune reactivity has been confirmed for endogenous retroviral and lncRNA peptide sequences. Using high‐resolution mass spectrometry, such epitopes relevant to several cancers have been discovered making possible therapies based on harnessing antigen presentation by means of vaccination, as well as T cell expansion and cell therapy [320, 321, 322, 323, 324, 325, 326, 327]. Some of the neoepitopes were generated from oncogenic driver mutations, not only lending to highly personalised anti‐cancer vaccination but to ‘off the shelf’ vaccines for individuals expressing HLA alleles of a supertype [327, 328, 329, 330]. Neoepitopes are not only generated by snSNPs, but can emerge from frameshift mutations via dysregulated alternative splicing and exitron splicing events, and microsatellite instability, all of which are hallmarks of tumorigenesis [331, 332, 333, 334]. We are just beginning to understand how a single amino acid alteration in a neoepitope beats immune tolerance to elicit an anti‐cancer response [335]. Until well‐learnt, we are at the mercy of combinatorial therapy such as checkpoint blockade, chemotherapy, or radiotherapy but at the cost of collateral damage (reviewed in refs. [331, 336]). In this regard, such therapies can benefit from oncolytic virus infections that cause immunopeptidome shifts as alluded to above, [45, 46, 47, 96, 100, 101, 164, 179, 191, 192, 193, 301]. This finding raises the intriguing possibility that oncolytic viruses, such as adenovirus or vaccinia virus [164], recombinant viruses that ferry innate immune adjuvants [337] or chemical agents that target specific cellular processes [338, 339], can aid to coax the expression and generation of neoepitopes to promote tumor immunity.

Finally, current insights into the nature, and the depth and breadth of immune self and non‐self [50, 51] screams silently how T cells view our health and disease states, tissue by tissue. An astute reader will have noted that nothing was said about MHC‐II immunopeptidomes. Reports of cancer MHC‐II restricted targetable neoepitopes and spliced autoantigens [42, 203, 340, 341, 342, 343, 344, 345, 346] have broken ground to lead to a finer definition of MHC‐II immunopeptidome. A deeper understanding of immune self and non‐self shall set us free from maladies of T cell malfunction (tumor immunosurveillance) and overactivity (autoimmunity and alloreactivity). Toward this end, the current state of the field foretells the excitement the new molecular cell biology and advances in translatome technologies will have on precisely defining immunologic self and non‐self.

## CONFLICT OF INTEREST

The authors declare no conflict of interest.

## Data Availability

This is a review article and no experimental data are included in the MS.

## References

[1] Davis, D. (2014). The compatibility gene: How our bodies fight disease, attract others, and define ourselves, Oxford University Press.

[2] Snell, G. D. , Dausset, J. , & Nathenson, S. G. (1976). Histocompatibility, Academic Press.

[3] Zinkernagel, R. M. , & Doherty, P. C. (1974). Immunological surveillance against altered self components by sensitised T lymphocytes in lymphocytic choriomeningitis. Nature, 251(5475), 547–548. 4547543 10.1038/251547a0

[4] Zinkernagel, R. M. , & Doherty, P. C. (1974). Restriction of in vitro T cell‐mediated cytotoxicity in lymphocytic choriomeningitis within a syngeneic or semiallogeneic system. Nature, 248(5450), 701–702. 4133807 10.1038/248701a0

[5] Klein, J. (1994). Alone on the heart of the earth: An immunogeneticist's journey into the past. Advances in Cancer Research, 63, 1–39. 8036986 10.1016/s0065-230x(08)60397-8

[6] Nathenson, S. G. , Uehara, H. , Ewenstein, B. M. , Kindt, T. J. , & Coligan, J. E. (1981). Primary structural: Analysis of the transplantation antigens of the murine H‐2 major histocompatibility complex. Annual Review of Biochemistry, 50, 1025–1052. 10.1146/annurev.bi.50.070181.005113 7023355

[7] Unanue, E. R. (2006). From antigen processing to peptide‐MHC binding. Nature Immunology, 7(12), 1277–1279. 10.1038/ni1206-1277 17110945

[8] Ziegler, H. K. , & Unanue, E. R. (1982). Decrease in macrophage antigen catabolism caused by ammonia and chloroquine is associated with inhibition of antigen presentation to T cells. Proceedings of the National Academy of Sciences of the United States of America, 79(1), 175–178. 6798568 10.1073/pnas.79.1.175 PMC345685

[9] Ziegler, H. K. , & Unanue, E. R. (1981). Identification of a macrophage antigen‐processing event required for I‐region‐restricted antigen presentation to T lymphocytes. Journal of Immunology, 127(5), 1869–1875. 6795263

[10] Chesnut, R. W. , Colon, S. M. , & Grey, H. M. (1982). Requirements for the processing of antigens by antigen‐presenting B cells. I. Functional comparison of B cell tumors and macrophages. Journal of Immunology, 129(6), 2382–2388. 6982920

[11] Grey, H. M. , Colon, S. M. , & Chesnut, R. W. (1982). Requirements for the processing of antigen by antigen‐presenting B cells. II. Biochemical comparison of the fate of antigen in B cell tumors and macrophages. Journal of Immunology, 129(6), 2389–2395. 6754811

[12] Chesnut, R. W. , Colon, S. M. , & Grey, H. M. (1982). Antigen presentation by normal B cells, B cell tumors, and macrophages: Functional and biochemical comparison. Journal of Immunology, 128(4), 1764–1768. 6977567

[13] Campbell, A. E. , Foley, F. L. , & Tevethia, S. S. (1983). Demonstration of multiple antigenic sites of the SV40 transplantation rejection antigen by using cytotoxic T lymphocyte clones. Journal of Immunology, 130(1), 490–492. 6183360

[14] Townsend, A. R. , McMichael, A. J. , Carter, N. P. , Huddleston, J. A. , & Brownlee, G. G. (1984). Cytotoxic T cell recognition of the influenza nucleoprotein and hemagglutinin expressed in transfected mouse L cells. Cell, 39(1), 13–25. 6091906 10.1016/0092-8674(84)90187-9

[15] Moore, M. W. , Carbone, F. R. , & Bevan, M. J. (1988). Introduction of soluble protein into the class I pathway of antigen processing and presentation. Cell, 54(6), 777–785. 3261634 10.1016/s0092-8674(88)91043-4

[16] Townsend, A. R. , Bastin, J. , Gould, K. , & Brownlee, G. G. (1986). Cytotoxic T lymphocytes recognize influenza haemagglutinin that lacks a signal sequence. Nature, 324(6097), 575–577. 10.1038/324575a0 3491325

[17] Yewdell, J. W. , Bennink, J. R. , & Hosaka, Y. (1988). Cells process exogenous proteins for recognition by cytotoxic T lymphocytes. Science, 239(4840), 637–640. 3257585 10.1126/science.3257585

[18] Babbitt, B. P. , Allen, P. M. , Matsueda, G. , Haber, E. , & Unanue, E. R. (1985). Binding of immunogenic peptides to Ia histocompatibility molecules. Nature, 317(6035), 359–361. 3876513 10.1038/317359a0

[19] Buus, S. , Colon, S. , Smith, C. , Freed, J. H. , Miles, C. , & Grey, H. M. (1986). Interaction between a “processed” ovalbumin peptide and Ia molecules. Proceedings of the National Academy of Sciences of the United States of America, 83(11), 3968–3971. 3487084 10.1073/pnas.83.11.3968 PMC323646

[20] Buus, S. , Sette, A. , Colon, S. M. , Jenis, D. M. , & Grey, H. M. (1986). Isolation and characterization of antigen‐Ia complexes involved in T cell recognition. Cell, 47(6), 1071–1077. 10.1016/0092-8674(86)90822-6 3490919

[21] Babbitt, B. P. , Matsueda, G. , Haber, E. , Unanue, E. R. , & Allen, P. M. (1986). Antigenic competition at the level of peptide‐Ia binding. Proceedings of the National Academy of Sciences of the United States of America, 83(12), 4509–4513. 10.1073/pnas.83.12.4509 3459185 PMC323763

[22] Bjorkman, P. J. , Saper, M. A. , Samraoui, B. , Bennett, W. S. , Strominger, J. L. , & Wiley, D. C. (1987). Structure of the human class I histocompatibility antigen, HLA‐A2. Nature, 329(6139), 506–512. 10.1038/329506a0 3309677

[23] Bjorkman, P. J. , Saper, M. A. , Samraoui, B. , Bennett, W. S. , Strominger, J. L. , & Wiley, D. C. (1987). The foreign antigen binding site and T cell recognition regions of class I histocompatibility antigens. Nature, 329(6139), 512–518. 10.1038/329512a0 2443855

[24] Matsumura, M. , Fremont, D. H. , Peterson, P. A. , & Wilson, I. A. (1992). Emerging principles for the recognition of peptide antigens by MHC class I molecules. Science, 257(5072), 927–934. 10.1126/science.1323878 1323878

[25] Fremont, D. H. , Matsumura, M. , Stura, E. A. , Peterson, P. A. , & Wilson, I. A. (1992). Crystal structures of two viral peptides in complex with murine MHC class I H‐2Kb. Science, 257(5072), 919–927. 10.1126/science.1323877 1323877

[26] Zhang, W. , Young, A. C. , Imarai, M. , Nathenson, S. G. , & Sacchettini, J. C. (1992). Crystal structure of the major histocompatibility complex class I H‐2Kb molecule containing a single viral peptide: implications for peptide binding and T‐cell receptor recognition. Proceedings of the National Academy of Sciences of the United States of America, 89(17), 8403–8407. 10.1073/pnas.89.17.8403 1325657 PMC49927

[27] Young, A. C. , Zhang, W. , Sacchettini, J. C. , & Nathenson, S. G. (1994). The three‐dimensional structure of H‐2Db at 2.4 A resolution: Implications for antigen‐determinant selection. Cell, 76(1), 39–50. 10.1016/0092-8674(94)90171-6 7506996

[28] Van Bleek, G. M. , & Nathenson, S. G. (1990). Isolation of an endogenously processed immunodominant viral peptide from the class I H‐2Kb molecule. Nature, 348(6298), 213–216. 10.1038/348213a0 1700303

[29] Falk, K. , Rotzschke, O. , & Rammensee, H. G. (1990). Cellular peptide composition governed by major histocompatibility complex class I molecules. Nature, 348(6298), 248–251. 10.1038/348248a0 2234092

[30] Rotzschke, O. , Falk, K. , Deres, K. , Schild, H. , Norda, M. , Metzger, J. , & Rammensee, H. G. (1990). Isolation and analysis of naturally processed viral peptides as recognized by cytotoxic T cells. Nature, 348(6298), 252–254. 10.1038/348252a0 1700304

[31] Wallny, H. J. , & Rammensee, H. G. (1990). Identification of classical minor histocompatibility antigen as cell‐derived peptide. Nature, 343(6255), 275–278. 10.1038/343275a0 1689009

[32] Falk, K. , Rotzschke, O. , Stevanovic, S. , Jung, G. , & Rammensee, H. G. (1991). Allele‐specific motifs revealed by sequencing of self‐peptides Eluted from MHC molecules. Nature, 351(6324), 290–296. 10.1038/351290a0 1709722

[33] Rudensky, A. , Rath, S. , Preston‐Hurlburt, P. , Murphy, D. B. , & Janeway, C. A., Jr. (1991). On the complexity of self. Nature, 353(6345), 660–662. 10.1038/353660a0 1656278

[34] Rudensky, Y. , Preston‐Hurlburt, P. , Hong, S. C. , Barlow, A. , & Janeway, C. A., Jr. (1991). Sequence analysis of peptides bound to MHC class II molecules. Nature, 353(6345), 622–627. 10.1038/353622a0 1656276

[35] Chicz, R. M. , Urban, R. G. , Lane, W. S. , Gorga, J. C. , Stern, L. J. , Vignali, D. A. , & Strominger, J. L. (1992). Predominant naturally processed peptides bound to HLA‐DR1 are derived from MHC‐related molecules and are heterogeneous in size. Nature, 358(6389), 764–768. 10.1038/358764a0 1380674

[36] Sette, A. , Ceman, S. , Kubo, R. T. , Sakaguchi, K. , Appella, E. , Hunt, D. F. , et al. (1992). Invariant chain peptides in most HLA‐DR molecules of an antigen‐processing mutant. Science, 258(5089), 1801–1804. 10.1126/science.1465617 1465617

[37] Hunt, D. F. , Michel, H. , Dickinson, T. A. , Shabanowitz, J. , Cox, A. L. , Sakaguchi, K. , Appella, E. , Grey, H. M. , & Sette, A. (1992). Peptides presented to the immune system by the murine class II major histocompatibility complex molecule I‐Ad. Science, 256(5065), 1817–1820. 10.1126/science.1319610 1319610

[38] Hunt, D. F. , Henderson, R. A. , Shabanowitz, J. , Sakaguchi, K. , Michel, H. , Sevilir, N. , Cox, A. L. , Appella, E. , & Engelhard, V. H. (1992). Characterization of peptides bound to the class I MHC molecule HLA‐A2.1 by mass spectrometry. Science, 255(5049), 1261–1263. 1546328 10.1126/science.1546328

[39] Zarling, A. L. , Ficarro, S. B. , White, F. M. , Shabanowitz, J. , Hunt, D. F. , & Engelhard, V. H. (2000). Phosphorylated peptides are naturally processed and presented by major histocompatibility complex class I molecules in vivo. Journal of Experimental Medicine, 192(12), 1755–1762. 10.1084/jem.192.12.1755 PMC2213507 11120772

[40] Zarling, A. L. , Polefrone, J. M. , Evans, A. M. , Mikesh, L. M. , Shabanowitz, J. , Lewis, S. T. , Engelhard, V. H. , & Hunt, D. F. (2006). Identification of class I MHC‐associated phosphopeptides as targets for cancer immunotherapy. Proceedings of the National Academy of Sciences of the United States of America, 103(40), 14889–14894. 10.1073/pnas.0604045103 17001009 PMC1595446

[41] Mohammed, F. , Cobbold, M. , Zarling, A. L. , Salim, M. , Barrett‐Wilt, G. A. , Shabanowitz, J. , Hunt, D. F. , Engelhard, V. H. , & Willcox, B. E. (2008). Phosphorylation‐dependent interaction between antigenic peptides and MHC class I: A molecular basis for the presentation of transformed self. Nature Immunology, 9(11), 1236–1243. 10.1038/ni.1660 18836451 PMC2596764

[42] Depontieu, F. R. , Qian, J. , Zarling, A. L. , McMiller, T. L. , Salay, T. M. , Norris, A. , English, A. M. , Shabanowitz, J. , Engelhard, V. H. , Hunt, D. F. , & Topalian, S. L. (2009). Identification of tumor‐associated, MHC class II‐restricted phosphopeptides as targets for immunotherapy. Proceedings of the National Academy of Sciences of the United States of America, 106(29), 12073–12078. 10.1073/pnas.0903852106 19581576 PMC2715484

[43] Cobbold, M. , De La Pena, H. , Norris, A. , Polefrone, J. M. , Qian, J. , English, A. M. , Cummings, K. L. , Penny, S. , Turner, J. E. , Cottine, J. , Abelin, J. G. , Malaker, S. A. , Zarling, A. L. , Huang, H. ‐. W. , Goodyear, O. , Freeman, S. D. , Shabanowitz, J. , Pratt, G. , Craddock, C. … Engelhard, V. H. (2013). MHC class I‐associated phosphopeptides are the targets of memory‐like immunity in leukemia. Science Translational Medicine, 5(203), 203ra125. 10.1126/scitranslmed.3006061 PMC4071620 24048523

[44] Engelhard, V. H. , Obeng, R. C. , Cummings, K. L. , Petroni, G. R. , Ambakhutwala, A. L. , Chianese‐Bullock, K. A. , Smith, K. T. , Lulu, A. , Varhegyi, N. , Smolkin, M. E. , Myers, P. , Mahoney, K. E. , Shabanowitz, J. , Buettner, N. , Hall, E. H. , Haden, K. , Cobbold, M. , Hunt, D. F. , Weiss, G. … Slingluff, C. L., Jr. (2020). MHC‐restricted phosphopeptide antigens: Preclinical validation and first‐in‐humans clinical trial in participants with high‐risk melanoma. Journal for ImmunoTherapy of Cancer, 8(1). 10.1136/jitc-2019-000262 PMC7228659 32385144

[45] Gilchuk, P. , Spencer, C. T. , Conant, S. B. , Hill, T. , Gray, J. J. , Niu, X. , Zheng, M. , Erickson, J. J. , Boyd, K. L. , McAfee, K. J. , Oseroff, C. , Hadrup, S. R. , Bennink, J. R. , Hildebrand, W. , Edwards, K. M. , Crowe, J. E. Jr , Williams, J. V. , Buus, S. , Sette, A. , Schumacher, T. N. M. , … Joyce, S. (2013). Discovering naturally processed antigenic determinants that confer protective T cell immunity. Journal of Clinical Investigation, 123(5), 1976–1987. 10.1172/JCI67388 PMC3635741 23543059

[46] Croft, N. P. , Smith, S. A. , Pickering, J. , Sidney, J. , Peters, B. , Faridi, P. , Witney, M. J. , Sebastian, P. , Flesch, I. E. A. , Heading, S. L. , Sette, A. , Gruta, N. L. L. , Purcell, A. W. , & Tscharke, D. C. (2019). Most viral peptides displayed by class I MHC on infected cells are immunogenic. Proceedings of the National Academy of Sciences of the United States of America, 116(8), 3112–3117. 10.1073/pnas.1815239116 30718433 PMC6386720

[47] McMurtrey, C. , Trolle, T. , Sansom, T. , Remesh, S. G. , Kaever, T. , Bardet, W. , Jackson, K. , McLeod, R. , Sette, A. , Nielsen, M. , Zajonc, D. M. , Blader, I. J. , Peters, B. , & Hildebrand, W. (2016). Toxoplasma gondii peptide ligands open the gate of the HLA class I binding groove. Elife, 5. 10.7554/eLife.12556 PMC4775218 26824387

[48] Parker, R. , Partridge, T. , Wormald, C. , Kawahara, R. , Stalls, V. , Aggelakopoulou, M. , Parker, J. , Doherty, R. P. , Morejon, Y. A. , Lee, E. , Saunders, K. , Haynes, B. F. , Acharya, P. , Thaysen‐Andersen, M. , Borrow, P. , & Ternette, N. (2020). Mapping the SARS‐CoV‐2 spike glycoprotein‐derived peptidome presented by HLA class II on dendritic cells. bioRxiv, 35, 109179. 10.1101/2020.08.19.255901 PMC8116342 34004174

[49] Sarkizova, S. , Klaeger, S. , Le, P. M. , Li, L. W. , Oliveira, G. , Keshishian, H. , Hartigan, C. R. , Zhang, W. , Braun, D. A. , Ligon, K. L. , Bachireddy, P. , Zervantonakis, I. K. , Rosenbluth, J. M. , Ouspenskaia, T. , Law, T. , Justesen, S. , Stevens, J. , Lane, W. J. , Eisenhaure, T. , Zhang, G. L. , … Keskin, D. B. (2020). A large peptidome dataset improves HLA class I epitope prediction across most of the human population. Nature Biotechnology, 38(2), 199–209. 10.1038/s41587-019-0322-9 PMC7008090 31844290

[50] Marcu, A. , Bichmann, L. , Kuchenbecker, L. , Kowalewski, D. , Freudenmann, L. , Backert, L. , Mühlenbruch, L. , Szolek, A. , Lübke, M. , Wagner, P. , Engler, T. , Matovina, S. , Wang, J. , Hauri‐Hohl, M. , Martin, R. , Kapolou, K. , Walz, J. S. , Velz, J. , Moch, H. , … Neidert, M. C. (2020). The HLA Ligand Atlas—A resource of natural HLA ligands presented on benign tissues. bioRxiv, 9, e002071. 10.1101/778944. PMC8054196 33858848

[51] Kubiniok, P. , Marcu, A. , Bichmann, L. , Kuchenbecker, L. , Schuster, H. , Hamelin, D. , Despault, J. , Kovalchik, K. , Wessling, L. , Kohlbacher, O. , Stevanovic, S. , Rammensee, H. ‐. G. , Neidert, M. C. , Sirois, I. , & Caron, E. (2020). The global architecture shaping the heterogeneity and tissue‐dependency of the MHC class I immunopeptidome is evolutionarily conserved. bioRxiv, 10.1101/2020.1109.1128.317750.

[52] Vizcaino, J. A. , Kubiniok, P. , Kovalchik, K. A. , Ma, Q. , Duquette, J. D. , Mongrain, I. , Deutsch, E. W. , Peters, B. , Sette, A. , Sirois, I. , & Caron, E. (2020). The human immunopeptidome project: A roadmap to predict and treat immune diseases. Molecular and Cellular Proteomics, 19(1), 31–49. 10.1074/mcp.R119.001743 31744855 PMC6944237

[53] Saper, M. , Bjorkman, P. J. , & Wiley, D. C. (1991). Refined structure of the human histocompatibility antigen at 2.6 ^o^A resolution. Journal of Molecular Biology, 219, 277–319. 2038058 10.1016/0022-2836(91)90567-p

[54] Kaufman, J. (2018). Generalists and specialists: A new view of how MHC class I molecules fight infectious pathogens. Trends in Immunology, 39(5), 367–379. 10.1016/j.it.2018.01.001 29396014 PMC5929564

[55] Spurgin, L. G. , & Richardson, D. S. (2010). How pathogens drive genetic diversity: MHC, mechanisms and misunderstandings. Proceedings: Biological Sciences, 277(1684), 979–988. 10.1098/rspb.2009.2084 20071384 PMC2842774

[56] Manczinger, M. , Boross, G. , Kemeny, L. , Muller, V. , Lenz, T. L. , Papp, B. , & Pal, C. (2019). Pathogen diversity drives the evolution of generalist MHC‐II alleles in human populations. Plos Biology, 17(1), e3000131. 10.1371/journal.pbio.3000131 30703088 PMC6372212

[57] Granados, D. P. , Sriranganadane, D. , Daouda, T. , Zieger, A. , Laumont, C. M. , Caron‐Lizotte, O. , Boucher, G. , Hardy, M. ‐. P. , Gendron, P. , Côté, C. , Lemieux, S. , Thibault, P. , & Perreault, C. (2014). Impact of genomic polymorphisms on the repertoire of human MHC class I‐associated peptides. Nature Communications, 5, 3600. 10.1038/ncomms4600 PMC3996541 24714562

[58] Jappe, E. C. , Kringelum, J. , Trolle, T. , & Nielsen, M. (2018). Predicted MHC peptide binding promiscuity explains MHC class I ‘hotspots’ of antigen presentation defined by mass spectrometry eluted ligand data. Immunology, 154(3), 407–417. 10.1111/imm.12905 29446062 PMC6002231

[59] Muller, M. , Gfeller, D. , Coukos, G. , & Bassani‐Sternberg, M. (2017). ‘Hotspots’ of antigen presentation revealed by human leukocyte antigen ligandomics for neoantigen prioritization. Frontiers in Immunology, 8, 1367. 10.3389/fimmu.2017.01367 29104575 PMC5654951

[60] Schumacher, T. N. , & Schreiber, R. D. (2015). Neoantigens in cancer immunotherapy. Science, 348(6230), 69–74. 10.1126/science.aaa4971 25838375

[61] Chowell, D. , Krishna, C. , Pierini, F. , Makarov, V. , Rizvi, N. A. , Kuo, F. , Morris, L. G. T. , Riaz, N. , Lenz, T. L. , & Chan, T. A. (2019). Evolutionary divergence of HLA class I genotype impacts efficacy of cancer immunotherapy. Nature Medicine, 25(11), 1715–1720. 10.1038/s41591-019-0639-4 PMC7938381 31700181

[62] McGranahan, N. , Rosenthal, R. , Hiley, C. T. , Rowan, A. J. , Watkins, T. B. K. , Wilson, G. A. , Birkbak, N. J. , Veeriah, S. , Van Loo, P. , Herrero, J. , Swanton, C. , & TRACERx Consortium (2017). Allele‐specific hla loss and immune escape in lung cancer evolution. Cell, 171(6), 1259–1271 e1211. 10.1016/j.cell.2017.10.001 29107330 PMC5720478

[63] Chowell, D. , Morris, L. G. T. , Grigg, C. M. , Weber, J. K. , Samstein, R. M. , Makarov, V. , Kuo, F. , Kendall, S. M. , Requena, D. , Riaz, N. , Greenbaum, B. , Carroll, J. , Garon, E. , Hyman, D. M. , Zehir, A. , Solit, D. , Berger, M. , Zhou, R. , Rizvi, N. A. , & Chan, T. A. (2018). Patient HLA class I genotype influences cancer response to checkpoint blockade immunotherapy. Science, 359(6375), 582–587. 10.1126/science.aao4572 29217585 PMC6057471

[64] Komov, L. , Kadosh, D. M. , Barnea, E. , Milner, E. , Hendler, A. , & Admon, A. (2018). Cell surface MHC class I expression is limited by the availability of peptide‐receptive “empty” molecules rather than by the supply of peptide ligands. Proteomics, 18(12), e1700248. 10.1002/pmic.201700248 29707912

[65] Joyce, S. (1997). Traffic control of completely assembled MHC class I molecules beyond the endoplasmic reticulum. Journal of Molecular Biology, 266(5), 993–1001. 10.1006/jmbi.1996.0822 9086276

[66] Chappell, P. , Meziane el, K. , Harrison, M. , Magiera, L. , Hermann, C. , Mears, L. , Wrobel, A. G. , Durant, C. , Nielsen, L. L. , Buus, S. , Ternette, N. , Mwangi, W. , Butter, C. , Nair, V. , Ahyee, T. , Duggleby, R. , Madrigal, A. , Roversi, P. , Lea, S. M. , & Kaufman, J. (2015). Expression levels of MHC class I molecules are inversely correlated with promiscuity of peptide binding. Elife, 4, e05345. 10.7554/eLife.05345 25860507 PMC4420994

[67] http://www.ebi.ac.uk/imgt/hla/allele.html . (2002, 11/11/2002). from http://www.ebi.ac.uk/imgt/hla/allele.html

[68] Lund, O. , Nielsen, M. , Kesmir, C. , Petersen, A. G. , Lundegaard, C. , Worning, P. , Sylvester‐Hvid, C. , Lamberth, K. , Røder, G. , Justesen, S. , Buus, S. , & Brunak, S. (2004). Definition of supertypes for HLA molecules using clustering of specificity matrices. Immunogenetics, 55(12), 797–810. 14963618 10.1007/s00251-004-0647-4

[69] Sette, A. , & Sidney, J. (1998). HLA supertypes and supermotifs: A functional perspective on HLA polymorphism. Current Opinion in Immunology, 10(4), 478–482. 10.1016/S0952-7915(98)80124-6 9722926

[70] Sette, A. , & Sidney, J. (1999). Nine major HLA class I supertypes account for the vast preponderance of HLA‐A and ‐B polymorphism. Immunogenetics, 50(3‐4), 201–212. 10.1016/90500201.251 10602880

[71] Robinson, J. , Barker, D. J. , Georgiou, X. , Cooper, M. A. , Flicek, P. , & Marsh, S. G. E. (2020). IPD‐IMGT/HLA database. Nucleic Acids Research, 48(D1), D948‐D955. 10.1093/nar/gkz950 31667505 PMC7145640

[72] Rammensee, H. G. , Bachmann, J. , & Stevanovic, S. (1998). MHC ligands and peptide motifs. Austin, R.G: Landes Bioscience.

[73] Bertoni, R. , Sette, A. , Sidney, J. , Guidotti, L. G. , Shapiro, M. , Purcell, R. , & Chisari, F. V. (1998). Human class I supertypes and CTL repertoires extend to chimpanzees. Journal of Immunology, 161(8), 4447–4455. 9780224

[74] del Guercio, M. F. , Sidney, J. , Hermanson, G. , Perez, C. , Grey, H. M. , Kubo, R. T. , & Sette, A. (1995). Binding of a peptide antigen to multiple HLA alleles allows definition of an A2‐like supertype. Journal of Immunology, 154(2), 685–693. 7529283

[75] Sidney, J. , Assarsson, E. , Moore, C. , Ngo, S. , Pinilla, C. , Sette, A. , & Peters, B. (2008). Quantitative peptide binding motifs for 19 human and mouse MHC class I molecules derived using positional scanning combinatorial peptide libraries. Immunome Research, 4, 2. 10.1186/1745-7580-4-2 18221540 PMC2248166

[76] Altfeld, M. A. , Livingston, B. , Reshamwala, N. , Nguyen, P. T. , Addo, M. M. , Shea, A. , Newman, M. , Fikes, J. , Sidney, J. , Wentworth, P. , Chesnut, R. , Eldridge, R. L. , Rosenberg, E. S. , Robbins, G. K. , Brander, C. , Sax, P. E. , Boswell, S. , Flynn, T. , Buchbinder, S. , … Kalams, S. A. (2001). Identification of novel HLA‐A2‐restricted human immunodeficiency virus type 1‐specific cytotoxic T‐lymphocyte epitopes predicted by the HLA‐A2 supertype peptide‐binding motif. Journal of Virology, 75(3), 1301–1311. 10.1128/JVI.75.3.1301-1311.2001 11152503 PMC114036

[77] Doolan, D. L. , Hoffman, S. L. , Southwood, S. , Wentworth, P. A. , Sidney, J. , Chesnut, R. W. , Keogh, E. , Appella, E. , Nutman, T. B. , Lal, A. A. , Gordon, D. M. , & Sette, A. (1997). Degenerate cytotoxic T cell epitopes from P. falciparum restricted by multiple HLA‐A and HLA‐B supertype alleles. Immunity, 7(1), 97–112. 9252123 10.1016/s1074-7613(00)80513-0

[78] Kawashima, I. , Tsai, V. , Southwood, S. , Takesako, K. , Celis, E. , & Sette, A. (1998). Identification of gp100‐derived, melanoma‐specific cytotoxic T‐lymphocyte epitopes restricted by HLA‐A3 supertype molecules by primary in vitro immunization with peptide‐pulsed dendritic cells. International Journal of Cancer, 78(4), 518–524. 9797143 10.1002/(sici)1097-0215(19981109)78:4<518::aid-ijc20>3.0.co;2-0

[79] Livingston, B. D. , Crimi, C. , Fikes, J. , Chesnut, R. W. , Sidney, J. , & Sette, A. (1999). Immunization with the HBV core 18–27 epitope elicits CTL responses in humans expressing different HLA‐A2 supertype molecules. Human Immunology, 60(11), 1013–1017. 10.1016/S0198-8859(99)00103-2 10599997

[80] Sidney, J. , Southwood, S. , Mann, D. L. , Fernandez‐Vina, M. A. , Newman, M. J. , & Sette, A. (2001). Majority of peptides binding HLA‐A*0201 with high affinity crossreact with other A2‐supertype molecules. Human Immunology, 62(11), 1200–1216. 11704282 10.1016/s0198-8859(01)00319-6

[81] Christinck, E. R. , Luscher, M. A. , Barber, B. H. , & Williams, D. B. (1991). Peptide binding to class I MHC on living cells and quantitation of complexes required for CTL lysis. Nature, 352(6330), 67–70. 10.1038/352067a0 2062379

[82] Harding, C. V. , & Unanue, E. R. (1990). Quantitation of antigen‐presenting cell MHC class II/peptide complexes necessary for T‐cell stimulation. Nature, 346(6284), 574–576. 10.1038/346574a0 2115981

[83] Yoshimura, Y. , Yadav, R. , Christianson, G. J. , Ajayi, W. U. , Roopenian, D. C. , & Joyce, S. (2004). Duration of alloantigen presentation and avidity of T cell antigen recognition correlate with immunodominance of CTL response to minor histocompatibility antigens. Journal of Immunology, 172(11), 6666–6674. 10.4049/jimmunol.172.11.6666 15153482

[84] Purbhoo, M. A. , Irvine, D. J. , Huppa, J. B. , & Davis, M. M. (2004). T cell killing does not require the formation of a stable mature immunological synapse. Nature Immunology, 5(5), 524–530. 10.1038/ni1058 15048111

[85] Jardetzky, T. S. , Lane, W. S. , Robinson, R. A. , Madden, D. R. , & Wiley, D. C. (1991). Identification of self peptides bound to purified HLA‐B27. Nature, 353(6342), 326–329. 10.1038/353326a0 1922338

[86] Yadav, R. , Yoshimura, Y. , Boesteanu, A. , Christianson, G. J. , Ajayi, W. U. , Shashidharamurthy, R. , Stanic, A. K. , Roopenian, D. C. , & Joyce, S. (2003). The H4b minor histocompatibility antigen is caused by a combination of genetically determined and posttranslational modifications. Journal of Immunology, 170(10), 5133–5142. 10.4049/jimmunol.170.10.5133 12734360

[87] Chen, W. , Anton, L. C. , Bennink, J. R. , & Yewdell, J. W. (2000). Dissecting the multifactorial causes of immunodominance in class I‐restricted T cell responses to viruses. Immunity, 12(1), 83–93. 10.1016/s1074-7613(00)80161-2 10661408

[88] Sturm, T. , Sautter, B. , Worner, T. P. , Stevanovic, S. , Rammensee, H. G. , Planz, O. , Heck, A. J. R. , & Aebersold, R. (2021). Mild acid elution and mhc immunoaffinity chromatography reveal similar albeit not identical profiles of the HLA Class I immunopeptidome. Journal of Proteome Research, 20(1), 289–304. 10.1021/acs.jproteome.0c00386 33141586 PMC7786382

[89] Antwi, K. , Hanavan, P. D. , Myers, C. E. , Ruiz, Y. W. , Thompson, E. J. , & Lake, D. F. (2009). Proteomic identification of an MHC‐binding peptidome from pancreas and breast cancer cell lines. Molecular Immunology, 46(15), 2931–2937. 10.1016/j.molimm.2009.06.021 19615748

[90] Lanoix, J. , Durette, C. , Courcelles, M. , Cossette, E. , Comtois‐Marotte, S. , Hardy, M. P. , Côté,, Perreault, C. , & Thibault, P. (2018). Comparison of the MHC I immunopeptidome repertoire of B‐Cell lymphoblasts using two isolation methods. Proteomics, 18(12), e1700251. 10.1002/pmic.201700251 29508533

[91] Corr, M. , Boyd, L. F. , Padlan, E. A. , & Margulies, D. H. (1993). H‐2Dd exploits a four residue peptide binding motif. Journal of Experimental Medicine, 178(6), 1877–1892. 10.1084/jem.178.6.1877 PMC2191296 8245770

[92] Corr, M. , Boyd, L. F. , Frankel, S. R. , Kozlowski, S. , Padlan, E. A. , & Margulies, D. H. (1992). Endogenous peptides of a soluble major histocompatibility complex class I molecule, H‐2Lds: Sequence motif, quantitative binding, and molecular modeling of the complex. Journal of Experimental Medicine, 176(6), 1681–1692. 10.1084/jem.176.6.1681 PMC2119472 1281216

[93] DiBrino, M. , Parker, K. C. , Shiloach, J. , Knierman, M. , Lukszo, J. , Turner, R. V. , Biddison, W. E. , & Coligan, J. E. (1993). Endogenous peptides bound to HLA‐A3 possess a specific combination of anchor residues that permit identification of potential antigenic peptides. Proceedings of the National Academy of Sciences of the United States of America, 90(4), 1508–1512. 10.1073/pnas.90.4.1508 7679507 PMC45903

[94] Joyce, S. , Tabaczewski, P. , Angeletti, R. H. , Nathenson, S. G. , & Stroynowski, I. (1994). A nonpolymorphic major histocompatibility complex class Ib molecule binds a large array of diverse self‐peptides. Journal of Experimental Medicine, 179(2), 579–588. 10.1084/jem.179.2.579 PMC2191392 8294869

[95] Hickman, H. D. , Luis, A. D. , Buchli, R. , Few, S. R. , Sathiamurthy, M. , VanGundy, R. S. , Giberson, C. F. , & Hildebrand, W. H. (2004). Toward a definition of self: Proteomic evaluation of the class I peptide repertoire. Journal of Immunology, 172(5), 2944–2952. 10.4049/jimmunol.172.5.2944 14978097

[96] Hickman, H. D. , Luis, A. D. , Bardet, W. , Buchli, R. , Battson, C. L. , Shearer, M. H. , Jackson, K. W. , Kennedy, R. C. , & Hildebrand, W. H. (2003). Cutting edge: Class I presentation of host peptides following HIV infection. Journal of Immunology, 171(1), 22–26. 10.4049/jimmunol.171.1.22 12816978

[97] Ljunggren, H. G. , Stam, N. J. , Ohlen, C. , Neefjes, J. J. , Hoglund, P. , Heemels, M. T. , … et al. (1990). Empty MHC class I molecules come out in the cold. Nature, 346(6283), 476–480. 10.1038/346476a0 2198471

[98] De Silva, A. D. , Boesteanu, A. , Song, R. , Nagy, N. , Harhaj, E. , Harding, C. V. , & Joyce, S. (1999). Thermolabile H‐2Kb molecules expressed by transporter associated with antigen processing‐deficient RMA‐S cells are occupied by low‐affinity peptides. Journal of Immunology, 163(8), 4413–4420. 10510382

[99] Wearsch, P. A. , Peaper, D. R. , & Cresswell, P. (2011). Essential glycan‐dependent interactions optimize MHC class I peptide loading. Proceedings of the National Academy of Sciences of the United States of America, 108(12), 4950–4955. 10.1073/pnas.1102524108 21383180 PMC3064348

[100] Yaciuk, J. C. , Skaley, M. , Bardet, W. , Schafer, F. , Mojsilovic, D. , Cate, S. , Stewart, C. J. , McMurtrey, C. , Jackson, K. W. , Buchli, R. , Olvera, A. , Cedeño, S. , Plana, M. , Mothe, B. , Brander, C. , & Hildebrand, W. H. (2014). Direct interrogation of viral peptides presented by the class I HLA of HIV‐infected T cells. Journal of Virology, 88(22), 12992–13004. 10.1128/JVI.01914-14 25165114 PMC4249081

[101] Kumar, A. , Suryadevara, N. C. , Wolf, K. J. , Wilson, J. T. , Di Paolo, R. J. , Brien, J. D. , & Joyce, S. (2020). Heterotypic immunity against vaccinia virus in an HLA‐B*07:02 transgenic mousepox infection model. Scientific Reports, 10(1), 13167. 10.1038/s41598-020-69897-w 32759969 PMC7406653

[102] Duan, F. , Duitama, J. , Al Seesi, S. , Ayres, C. M. , Corcelli, S. A. , Pawashe, A. P. , Blanchard, T. , Mcmahon, D. , Sidney, J. , Sette, A. , Baker, B. M. , Mandoiu, I. I. , & Srivastava, P. K. (2014). Genomic and bioinformatic profiling of mutational neoepitopes reveals new rules to predict anticancer immunogenicity. Journal of Experimental Medicine, 211(11), 2231–2248. 10.1084/jem.20141308 PMC4203949 25245761

[103] Gubin, M. M. , Zhang, X. , Schuster, H. , Caron, E. , Ward, J. P. , Noguchi, T. , Ivanova, Y. , Hundal, J. , Arthur, C. D. , Krebber, W. ‐. J. , Mulder, G. E. , Toebes, M. , Vesely, M. D. , Lam, S. S. K. , Korman, A. J. , Allison, J. P. , Freeman, G. J. , Sharpe, A. H. , Pearce, E. L. , Schumacher, T. N. … Schreiber, R. D. (2014). Checkpoint blockade cancer immunotherapy targets tumour‐specific mutant antigens. Nature, 515(7528), 577–581. 10.1038/nature13988 25428507 PMC4279952

[104] Kowalewski, D. J. , Schuster, H. , Backerta, L. , Berlina, C. , Kahn, S. , Kanz, L. , Salih, H. R. , Rammensee, H. ‐ G. , Stevanovic, S. , & Stickel, J. S. (2014). HLA ligandome analysis identifies the underlying specificities of spontaneous antileukemia immune responses in chronic lymphocytic leukemia (CLL). Proceedings of the National Academy of Sciences of the United States of America, in press. 10.1073/pnas.1416389112 PMC4299203 25548167

[105] Yadav, M. , Jhunjhunwala, S. , Phung, Q. T. , Lupardus, P. , Tanguay, J. , Bumbaca, S. , Franci, C. , Cheung, T. K. , Fritsche, J. , Weinschenk, T. , Modrusan, Z. , Mellman, I. , Lill, J. R. , & Delamarre, L. (2014). Predicting immunogenic tumour mutations by combining mass spectrometry and exome sequencing. Nature, 515(7528), 572–576. 10.1038/nature14001 25428506

[106] Bassani‐Sternberg, M. , Bräunlein, E. , Klar, R. , Engleitner, T. , Sinitcyn, P. , Audehm, S. , Straub, M. , Weber, J. , Slotta‐Huspenina, J. , Specht, K. , Martignoni, M. E. , Werner, A. , Hein, R. H. , Busch, D. , Peschel, C. , Rad, R. , Cox, J. , Mann, M. , & Krackhardt, A. M. (2016). Direct identification of clinically relevant neoepitopes presented on native human melanoma tissue by mass spectrometry. Nature Communications, 7, 13404. 10.1038/ncomms13404 PMC5121339 27869121

[107] Schuster, H. , Peper, J. K. , Bösmüller, H.‐C. , Röhle, K. , Backert, L. , Bilich, T. , Ney, B. , Löffler, M. W. , Kowalewski, D. J. , Trautwein, N. , Rabsteyn, A. , Engler, T. , Braun, S. , Haen, S. P. , Walz, J. S. , Schmid‐Horch, B. , Brucker, S. Y. , Wallwiener, D. , Kohlbacher, O. … Wagner, P. (2017). The immunopeptidomic landscape of ovarian carcinomas. Proceedings of the National Academy of Sciences of the United States of America, 114(46), E9942‐E9951. 10.1073/pnas.1707658114 29093164 PMC5699044

[108] Ternette, N. , Olde Nordkamp, M. J. M. , Muller, J. , Anderson, A. P. , Nicastri, A. , Hill, A. V. S. , Kessler, B. M. , & Li, D. (2018). Immunopeptidomic profiling of HLA‐A2‐positive triple negative breast cancer identifies potential immunotherapy target antigens. Proteomics, 18(12), e1700465. 10.1002/pmic.201700465 29786170 PMC6032843

[109] Newey, A. , Griffiths, B. , Michaux, J. , Pak, H. S. , Stevenson, B. J. , Woolston, A. , Semiannikova, M. , Spain, G. , Barber, L. J. , Matthews, N. , Rao, S. , Watkins, D. , Chau, I. , Coukos, G. , Racle, J. , Gfeller, D. , Starling, N. , Cunningham, D. , Bassani‐Sternberg, M. , & Gerlinger, M. (2019). Immunopeptidomics of colorectal cancer organoids reveals a sparse HLA class I neoantigen landscape and no increase in neoantigens with interferon or MEK‐inhibitor treatment. Journal for ImmunoTherapy of Cancer, 7(1), 309. 10.1186/s40425-019-0769-8 31735170 PMC6859637

[110] Nicastri, A. , Liao, H. , Muller, J. , Purcell, A. W. , & Ternette, N. (2020). The choice of HLA‐associated peptide enrichment and purification strategy affects peptide yields and creates a bias in detected sequence repertoire. Proteomics, 20(12), e1900401. 10.1002/pmic.201900401 32359108

[111] Purcell, A. W. , Ramarathinam, S. H. , & Ternette, N. (2019). Mass spectrometry‐based identification of MHC‐bound peptides for immunopeptidomics. Nature Protocols, 14(6), 1687–1707. 10.1038/s41596-019-0133-y 31092913

[112] Pandey, K. , Mifsud, N. A. , Lim Kam Sian, T. C. C. , Ayala, R. , Ternette, N. , Ramarathinam, S. H. , & Purcell, A. W. (2020). In‐depth mining of the immunopeptidome of an acute myeloid leukemia cell line using complementary ligand enrichment and data acquisition strategies. Molecular Immunology, 123, 7–17. 10.1016/j.molimm.2020.04.008 32387766

[113] Abelin, J. G. , Keskin, D. B. , Sarkizova, S. , Hartigan, C. R. , Zhang, W. , Sidney, J. , Stevens, J. , Lane, W. , Zhang, G. L. , Eisenhaure, T. M. , Clauser, K. R. , Hacohen, N. , Rooney, M. S. , Carr, S. A. , & Wu, C. J. (2017). Mass spectrometry profiling of HLA‐Associated peptidomes in mono‐allelic cells enables more accurate epitope prediction. Immunity, 46(2), 315–326. 10.1016/j.immuni.2017.02.007 28228285 PMC5405381

[114] Krangel, M. S. , Orr, H. T. , & Strominger, J. L. (1979). Assembly and maturation of HLA‐A and HLA‐B antigens in vivo. Cell, 18(4), 979–991. 10.1016/0092-8674(79)90210-1 93026

[115] Ploegh, H. L. , Orr, H. T. , & Strominger, J. L. (1981). Major histocompatibility antigens: The human (HLA‐A, ‐B, ‐C) and murine (H‐2K, H‐2D) class I molecules. Cell, 24(2), 287–299. 10.1016/0092-8674(81)90318-4 7016338

[116] Shepherd, J. C. , Schumacher, T. N. , Ashton‐Rickardt, P. G. , Imaeda, S. , Ploegh, H. L. , Janeway, C. A., Jr. , & Tonegawa, S. (1993). TAP1‐dependent peptide translocation in vitro is ATP dependent and peptide selective. Cell, 74(3), 577–584. 10.1016/0092-8674(93)80058-m 8348620

[117] Schumacher, T. N. , Kantesaria, D. V. , Heemels, M. T. , Ashton‐Rickardt, P. G. , Shepherd, J. C. , Fruh, K. , Yang, Y. , Peterson, P. A. , Tonegawa, S. , & Ploegh, H. L. (1994). Peptide length and sequence specificity of the mouse TAP1/TAP2 translocator. Journal of Experimental Medicine, 179(2), 533–540. 10.1084/jem.179.2.533 PMC2191358 8294864

[118] Heemels, M. T. , Schumacher, T. N. , Wonigeit, K. , & Ploegh, H. L. (1993). Peptide translocation by variants of the transporter associated with antigen processing. Science, 262(5142), 2059–2063. 10.1126/science.8266106 8266106

[119] Androlewicz, M. J. , Anderson, K. S. , & Cresswell, P. (1993). Evidence that transporters associated with antigen processing translocate a major histocompatibility complex class I‐binding peptide into the endoplasmic reticulum in an ATP‐dependent manner. Proceedings of the National Academy of Sciences of the United States of America, 90(19), 9130–9134. 10.1073/pnas.90.19.9130 8415666 PMC47515

[120] Androlewicz, M. J. , Ortmann, B. , van Endert, P. M. , Spies, T. , & Cresswell, P. (1994). Characteristics of peptide and major histocompatibility complex class I/beta 2‐microglobulin binding to the transporters associated with antigen processing (TAP1 and TAP2). Proceedings of the National Academy of Sciences of the United States of America, 91(26), 12716–12720. 10.1073/pnas.91.26.12716 7809108 PMC45510

[121] Blum, J. S. , Wearsch, P. A. , & Cresswell, P. (2013). Pathways of antigen processing. Annual Review of Immunology, 31, 443–473. 10.1146/annurev-immunol-032712-095910 PMC4026165 23298205

[122] Degen, E. , & Williams, D. B. (1991). Participation of a novel 88‐kD protein in the biogenesis of murine class I histocompatibility molecules. Journal of Cell Biology, 112(6), 1099–1115. 10.1083/jcb.112.6.1099 PMC2288894 1999467

[123] Jackson, M. R. , Cohen‐Doyle, M. F. , Peterson, P. A. , & Williams, D. B. (1994). Regulation of MHC class I transport by the molecular chaperone, calnexin (p88, IP90). Science, 263(5145), 384–387. 10.1126/science.8278813 8278813

[124] Rajagopalan, S. , & Brenner, M. B. (1994). Calnexin retains unassembled major histocompatibility complex class I free heavy chains in the endoplasmic reticulum. Journal of Experimental Medicine, 180(1), 407–412. 10.1084/jem.180.1.407 PMC2191536 8006598

[125] Vassilakos, A. , Cohen‐Doyle, M. F. , Peterson, P. A. , Jackson, M. R. , & Williams, D. B. (1996). The molecular chaperone calnexin facilitates folding and assembly of class I histocompatibility molecules. EMBO Journal, 15(7), 1495–1506. PMC450057 8612572

[126] Ou, W. J. , Cameron, P. H. , Thomas, D. Y. , & Bergeron, J. J. (1993). Association of folding intermediates of glycoproteins with calnexin during protein maturation. Nature, 364(6440), 771–776. 10.1038/364771a0 8102790

[127] Rajagopalan, S. , Xu, Y. , & Brenner, M. B. (1994). Retention of unassembled components of integral membrane proteins by calnexin. Science, 263(5145), 387–390. 10.1126/science.8278814 8278814

[128] Hebert, D. N. , Foellmer, B. , & Helenius, A. (1996). Calnexin and calreticulin promote folding, delay oligomerization and suppress degradation of influenza hemagglutinin in microsomes. EMBO Journal, 15(12), 2961–2968. PMC450237 8670797

[129] Wearsch, P. A. , & Cresswell, P. (2007). Selective loading of high‐affinity peptides onto major histocompatibility complex class I molecules by the tapasin‐ERp57 heterodimer. Nature Immunology, 8(8), 873–881. 10.1038/ni1485 17603487

[130] Dong, G. , Wearsch, P. A. , Peaper, D. R. , Cresswell, P. , & Reinisch, K. M. (2009). Insights into MHC class I peptide loading from the structure of the tapasin‐ERp57 thiol oxidoreductase heterodimer. Immunity, 30(1), 21–32. 10.1016/j.immuni.2008.10.018 19119025 PMC2650231

[131] Thomas, C. , & Tampe, R. (2019). MHC I chaperone complexes shaping immunity. Current Opinion in Immunology, 58, 9–15. 10.1016/j.coi.2019.01.001 30771631

[132] Koopmann, J. O. , Post, M. , Neefjes, J. J. , Hammerling, G. J. , & Momburg, F. (1996). Translocation of long peptides by transporters associated with antigen processing (TAP). European Journal of Immunology, 26(8), 1720–1728. 10.1002/eji.1830260809 8765012

[133] Burgevin, A. , Saveanu, L. , Kim, Y. , Barilleau, E. , Kotturi, M. , Sette, A. , van Endert, P. , & Peters, B. (2008). A detailed analysis of the murine TAP transporter substrate specificity. Plos One, 3(6), e2402. 10.1371/journal.pone.0002402 18545702 PMC2408963

[134] Hammer, G. E. , Gonzalez, F. , James, E. , Nolla, H. , & Shastri, N. (2007). In the absence of aminopeptidase ERAAP, MHC class I molecules present many unstable and highly immunogenic peptides. Nature Immunology, 8(1), 101–108. 10.1038/ni1409 17128277

[135] Kanaseki, T. , Blanchard, N. , Hammer, G. E. , Gonzalez, F. , & Shastri, N. (2006). ERAAP synergizes with MHC class I molecules to make the final cut in the antigenic peptide precursors in the endoplasmic reticulum. Immunity, 25(5), 795–806. 10.1016/j.immuni.2006.09.012 17088086 PMC2746443

[136] Hammer, G. E. , Gonzalez, F. , Champsaur, M. , Cado, D. , & Shastri, N. (2006). The aminopeptidase ERAAP shapes the peptide repertoire displayed by major histocompatibility complex class I molecules. Nature Immunology, 7(1), 103–112. 10.1038/ni1286 16299505

[137] Serwold, T. , Gonzalez, F. , Kim, J. , Jacob, R. , & Shastri, N. (2002). ERAAP customizes peptides for MHC class I molecules in the endoplasmic reticulum. Nature, 419(6906), 480–483. 10.1038/nature01074 12368856

[138] York, I. A. , Chang, S. C. , Saric, T. , Keys, J. A. , Favreau, J. M. , Goldberg, A. L. , & Rock, K. L. (2002). The ER aminopeptidase ERAP1 enhances or limits antigen presentation by trimming epitopes to 8–9 residues. Nature Immunology, 3(12), 1177–1184. 10.1038/ni860 12436110

[139] Saric, T. , Chang, S. C. , Hattori, A. , York, I. A. , Markant, S. , Rock, K. L. , Tsujimoto, M. , & Goldberg, A. L. (2002). An IFN‐gamma‐induced aminopeptidase in the ER, ERAP1, trims precursors to MHC class I‐presented peptides. Nature Immunology, 3(12), 1169–1176. 10.1038/ni859 12436109

[140] Yan, J. , Parekh, V. V. , Mendez‐Fernandez, Y. , Olivares‐Villagomez, D. , Dragovic, S. , Hill, T. , Roopenian, D. C. , Joyce, S. , & Van Kaer, L. (2006). In vivo role of ER‐associated peptidase activity in tailoring peptides for presentation by MHC class Ia and class Ib molecules. Journal of Experimental Medicine, 203(3), 647–659. 10.1084/jem.20052271 PMC2118255 16505142

[141] Saveanu, L. , Carroll, O. , Lindo, V. , Del Val, M. , Lopez, D. , Lepelletier, Y. , Greer, F. , Schomburg, L. , Fruci, D. , Niedermann, G. , & Van Endert, P. M. (2005). Concerted peptide trimming by human ERAP1 and ERAP2 aminopeptidase complexes in the endoplasmic reticulum. Nature Immunology, 6(7), 689–697. 10.1038/ni1208 15908954

[142] Blanchard, N. , Kanaseki, T. , Escobar, H. , Delebecque, F. , Nagarajan, N. A. , Reyes‐Vargas, E. , Crockett, D. K. , Raulet, D. H. , Delgado, J. C. , & Shastri, N. (2010). Endoplasmic reticulum aminopeptidase associated with antigen processing defines the composition and structure of MHC class I peptide repertoire in normal and virus‐infected cells. Journal of Immunology, 184(6), 3033–3042. 10.4049/jimmunol.0903712 PMC3087292 20173027

[143] Kirino, Y. , Bertsias, G. , Ishigatsubo, Y. , Mizuki, N. , Tugal‐Tutkun, I. , Seyahi, E. , Ozyazgan, Y. , Sacli, F. S. , Erer, B. , Inoko, H. , Emrence, Z. , Cakar, A. , Abaci, N. , Ustek, D. , Satorius, C. , Ueda, A. , Takeno, M. , Kim, Y. , Wood, G. M. , … Kastner, D. L. (2013). Genome‐wide association analysis identifies new susceptibility loci for Behcet's disease and epistasis between HLA‐B*51 and ERAP1. Nature Genetics, 45(2), 202–207. 10.1038/ng.2520 23291587 PMC3810947

[144] Evans, D. M. , Spencer, C. C. A. , Pointon, J. J. , Su, Z. , Harvey, D. , Kochan, G. , Oppermann, U. , Dilthey, A. , Pirinen, M. , Stone, M. A. , Appleton, L. , Moutsianas, L. , Leslie, S. , Wordsworth, T. , Kenna, T. J. , Karaderi, T. , Thomas, G. P. , Ward, M. M. , Weisman, M. H. … Wellcome Trust Case Control, C. (2011). Interaction between ERAP1 and HLA‐B27 in ankylosing spondylitis implicates peptide handling in the mechanism for HLA‐B27 in disease susceptibility. Nature Genetics, 43(8), 761–767. 10.1038/ng.873 21743469 PMC3640413

[145] Strange, A. , Capon, F. , Spencer, C. C. , Knight, J. , Weale, M. E. , Allen, M. H. , Barton, A. , Band, G. , Bellenguez, C. , Bergboer, J. G. M. , Blackwell, J. M. , Bramon, E. , Bumpstead, S. J. , Casas, J. P. , Cork, M. J. , Corvin, A. , Deloukas, P. , Dilthey, A. , Duncanson, A. , Edkins, S. … Genetic Analysis of Psoriasis Consortium & the Wellcome Trust Case Control Consortium 2. (2010). A genome‐wide association study identifies new psoriasis susceptibility loci and an interaction between HLA‐C and ERAP1. Nature Genetics, 42(11), 985–990. 10.1038/ng.694 20953190 PMC3749730

[146] Lopez de Castro, J. A. (2018). How ERAP1 and ERAP2 shape the peptidomes of disease‐associated MHC‐I proteins. Frontiers in Immunology, 9, 2463. 10.3389/fimmu.2018.02463 30425713 PMC6219399

[147] Yao, Y. , Liu, N. , Zhou, Z. , & Shi, L. (2019). Influence of ERAP1 and ERAP2 gene polymorphisms on disease susceptibility in different populations. Human Immunology, 80(5), 325–334. 10.1016/j.humimm.2019.02.011 30797823

[148] Tedeschi, V. , Paldino, G. , Paladini, F. , Mattorre, B. , Tuosto, L. , Sorrentino, R. , & Fiorillo, M. T. (2020). The Impact of the ‘Mis‐Peptidome’ on HLA Class I‐Mediated Diseases: Contribution of ERAP1 and ERAP2 and Effects on the Immune Response. International Journal of Molecular Sciences, 21(24). 10.3390/ijms21249608 PMC7765998 33348540

[149] Reeves, E. , Edwards, C. J. , Elliott, T. , & James, E. (2013). Naturally occurring ERAP1 haplotypes encode functionally distinct alleles with fine substrate specificity. Journal of Immunology, 191(1), 35–43. 10.4049/jimmunol.1300598 PMC3785127 23733883

[150] Martín‐Esteban, A. , Gómez‐Molina, P. , Sanz‐Bravo, A. , & López De Castro, J. A. (2014). Combined effects of ankylosing spondylitis‐associated ERAP1 polymorphisms outside the catalytic and peptide‐binding sites on the processing of natural HLA‐B27 ligands. Journal of Biological Chemistry, 289(7), 3978–3990. 10.1074/jbc.M113.529610 PMC3924265 24352655

[151] Goto, Y. , Ogawa, K. , Nakamura, T. J. , Hattori, A. , & Tsujimoto, M. (2015). Substrate‐dependent nitric oxide synthesis by secreted endoplasmic reticulum aminopeptidase 1 in macrophages. Journal of Biochemistry, 157(6), 439–449. 10.1093/jb/mvv001 25577645

[152] Evnouchidou, I. , Kamal, R. P. , Seregin, S. S. , Goto, Y. , Tsujimoto, M. , Hattori, A. , Voulgari, P. V. , Drosos, A. A. , Amalfitano, A. , York, I. A. , & Stratikos, E. (2011). Cutting edge: Coding single nucleotide polymorphisms of endoplasmic reticulum aminopeptidase 1 can affect antigenic peptide generation in vitro by influencing basic enzymatic properties of the enzyme. Journal of Immunology, 186(4), 1909–1913. 10.4049/jimmunol.1003337 PMC4039038 21242517

[153] Stamogiannos, A. , Koumantou, D. , Papakyriakou, A. , & Stratikos, E. (2015). Effects of polymorphic variation on the mechanism of endoplasmic reticulum aminopeptidase 1. Molecular Immunology, 67(2 Pt) 426–435. 10.1016/j.molimm.2015.07.010 26224046

[154] Martín‐Esteban, A. , Sanz‐Bravo, A. , Guasp, P. , Barnea, E. , Admon, A. , & López De Castro, J. A. (2017). Separate effects of the ankylosing spondylitis associated ERAP1 and ERAP2 aminopeptidases determine the influence of their combined phenotype on the HLA‐B*27 peptidome. Journal of Autoimmunity, 79, 28–38. 10.1016/j.jaut.2016.12.008 28063628

[155] Martín‐Esteban, A. , Guasp, P. , Barnea, E. , Admon, A. , & López De Castro, J. A. (2016). Functional interaction of the ankylosing spondylitis‐associated endoplasmic reticulum aminopeptidase 2 with the HLA‐B*27 peptidome in human cells. Arthritis Rheumatology, 68(10), 2466–2475. 10.1002/art.39734 27110896

[156] Chen, L. , Shi, H. , Koftori, D. , Sekine, T. , Nicastri, A. , Ternette, N. , & Bowness, P. (2020). Identification of an unconventional subpeptidome bound to the Behcet's disease‐associated HLA‐B*51:01 that is regulated by endoplasmic reticulum aminopeptidase 1 (ERAP1). Molecular and Cellular Proteomics, 19(5), 871–883. 10.1074/mcp.RA119.001617 32161166 PMC7196583

[157] James, E. , Bailey, I. , Sugiyarto, G. , & Elliott, T. (2013). Induction of protective antitumor immunity through attenuation of ERAAP function. Journal of Immunology, 190(11), 5839–5846. 10.4049/jimmunol.1300220 23610143

[158] Vance, R. E. , Kraft, J. R. , Altman, J. D. , Jensen, P. E. , & Raulet, D. H. (1998). Mouse CD94/NKG2A is a natural killer cell receptor for the nonclassical major histocompatibility complex (MHC) class I molecule Qa‐1(b). Journal of Experimental Medicine, 188(10), 1841–1848. 10.1084/jem.188.10.1841 PMC2212405 9815261

[159] Braud, V. M. , Allan, D. S. J. , O'callaghan, C. A. , Söderström, K. , D'andrea, A. , Ogg, G. S. , Lazetic, S. , Young, N. T. , Bell, J. I. , Phillips, J. H. , Lanier, L. L. , & Mcmichael, A. J. (1998). HLA‐E binds to natural killer cell receptors CD94/NKG2A, B and C. Nature, 391(6669), 795–799. 10.1038/35869 9486650

[160] Borrego, F. , Ulbrecht, M. , Weiss, E. H. , Coligan, J. E. , & Brooks, A. G. (1998). Recognition of human histocompatibility leukocyte antigen (HLA)‐E complexed with HLA class I signal sequence‐derived peptides by CD94/NKG2 confers protection from natural killer cell‐mediated lysis. Journal of Experimental Medicine, 187(5), 813–818. 10.1084/jem.187.5.813 PMC2212178 9480992

[161] Guan, J. , Yang, S. J. , Gonzalez, F. , Yin, Y. , & Shastri, N. (2017). Antigen processing in the endoplasmic reticulum is monitored by semi‐invariant alphabeta TCRs specific for a conserved peptide‐qa‐1(b) MHC class Ib ligand. Journal of Immunology, 198(5), 2017–2027. 10.4049/jimmunol.1600764 PMC5321846 28108559

[162] Nagarajan, N. A. , De Verteuil, D. A. , Sriranganadane, D. , Yahyaoui, W. , Thibault, P. , Perreault, C. , & Shastri, N. (2016). ERAAP Shapes the Peptidome Associated with Classical and Nonclassical MHC Class I Molecules. Journal of Immunology, 197(4), 1035–1043. 10.4049/jimmunol.1500654 PMC4976029 27371725

[163] Dragovic, S. M. , Hill, T. , Christianson, G. J. , Kim, S. , Elliott, T. , Scott, D. , Roopenian, D. C. , Van Kaer, L. , & Joyce, S. (2011). Proteasomes, TAP, and endoplasmic reticulum‐associated aminopeptidase associated with antigen processing control CD4+ Th cell responses by regulating indirect presentation of MHC class II‐restricted cytoplasmic antigens. Journal of Immunology, 186(12), 6683–6692. 10.4049/jimmunol.1100525 PMC3537507 21572029

[164] Spencer, C. T. , Bezbradica, J. S. , Ramos, M. G. , Arico, C. D. , Conant, S. B. , Gilchuk, P. , Gray, J. J. , Zheng, Mu , Niu, X. , Hildebrand, W. , Link, A. J. , & Joyce, S. (2015). Viral infection causes a shift in the self peptide repertoire presented by human MHC class I molecules. PROTEOMICS – Clinical Applications, 9(11‐12), 1035–1052. 10.1002/prca.201500106 26768311 PMC4920078

[165] Rizvi, S. M. , & Raghavan, M. (2006). Direct peptide‐regulatable interactions between MHC class I molecules and tapasin. Proceedings of the National Academy of Sciences of the United States of America, 103(48), 18220–18225. 10.1073/pnas.0605131103 17116884 PMC1838733

[166] Chen, M. , & Bouvier, M. (2007). Analysis of interactions in a tapasin/class I complex provides a mechanism for peptide selection. EMBO Journal, 26(6), 1681–1690. 10.1038/sj.emboj.7601624 PMC1829385 17332746

[167] Williams, A. P. , Peh, CAu , Purcell, A. W. , Mccluskey, J. , & Elliott, T. (2002). Optimization of the MHC class I peptide cargo is dependent on tapasin. Immunity, 16(4), 509–520. 10.1016/s1074-7613(02)00304-7 11970875

[168] Grandea, A. G. , Golovina, T. N. , Hamilton, S. E. , Sriram, V. , Spies, T. , Brutkiewicz, R. R. , Harty, J. T. , Eisenlohr, L. C. , & Van Kaer, L. (2000). Impaired assembly yet normal trafficking of MHC class I molecules in Tapasin mutant mice. Immunity, 13(2), 213–222. 10.1016/s1074-7613(00)00021-2 10981964

[169] Thomas, C. , & Tampe, R. (2017). Structure of the TAPBPR‐MHC I complex defines the mechanism of peptide loading and editing. Science, 358(6366), 1060–1064. 10.1126/science.aao6001 29025996

[170] Jiang, J. , Natarajan, K. , Boyd, L. F. , Morozov, G. I. , Mage, M. G. , & Margulies, D. H. (2017). Crystal structure of a TAPBPR‐MHC I complex reveals the mechanism of peptide editing in antigen presentation. Science, 358(6366), 1064–1068. 10.1126/science.aao5154 29025991 PMC6320693

[171] Overall, S. A. , Toor, J. S. , Hao, S. , Yarmarkovich, M. , Sara, M. O'rourke , Morozov, G. I. , Nguyen, S. , Japp, A. S. , Gonzalez, N. , Moschidi, D. , Betts, M. R. , Maris, J. M. , Smibert, P. , & Sgourakis, N. G. (2020). High throughput pMHC‐I tetramer library production using chaperone‐mediated peptide exchange. Nature Communications, 11(1), 1909. 10.1038/s41467-020-15710-1 PMC7170893 32312993

[172] Peh, C. A. , Burrows, S. R. , Barnden, M. , Khanna, R. , Cresswell, P. , Moss, D. J. , & McCluskey, J. (1998). HLA‐B27‐restricted antigen presentation in the absence of tapasin reveals polymorphism in mechanisms of HLA class I peptide loading. Immunity, 8(5), 531–542. 10.1016/s1074-7613(00)80558-0 9620674

[173] Rizvi, S. M. , Salam, N. , Geng, J. , Qi, Y. , Bream, J. H. , Duggal, P. , Hussain, S. K. , Martinson, J. , Wolinsky, S. M. , Carrington, M. , & Raghavan, M. (2014). Distinct assembly profiles of HLA‐B molecules. Journal of Immunology, 192(11), 4967–4976. 10.4049/jimmunol.1301670 PMC4117407 24790147

[174] Bashirova, A. A. , Viard, M. , Naranbhai, V. , Grifoni, A. , Garcia‐Beltran, W. , Akdag, M. , Yuki, Y. , Gao, X. , O'huigin, C. , Raghavan, M. , Wolinsky, S. , Bream, J. H. , Duggal, P. , Martinson, J. , Michael, N. L. , Kirk, G. D. , Buchbinder, S. P. , Haas, D. , Goedert, J. J. … Carrington, M. (2020). HLA tapasin independence: Broader peptide repertoire and HIV control. Proceedings of the National Academy of Sciences of the United States of America, 117(45), 28232–28238. 10.1073/pnas.2013554117 33097667 PMC7668082

[175] Princiotta, M. F. , Finzi, D. , Qian, S.‐B. , Gibbs, J. , Schuchmann, S. , Buttgereit, F. , Bennink, J. R. , & Yewdell, J. W. (2003). Quantitating protein synthesis, degradation, and endogenous antigen processing. Immunity, 18(3), 343–354. 10.1016/s1074-7613(03)00051-7 12648452

[176] Schwanhäusser, B. , Busse, D. , Li, Na , Dittmar, G. , Schuchhardt, J. , Wolf, J. , Chen, W. , & Selbach, M. (2011). Global quantification of mammalian gene expression control. Nature, 473(7347), 337–342. 10.1038/nature10098 21593866

[177] Esquivel, F. , Yewdell, J. , & Bennink, J. (1992). RMA/S cells present endogenously synthesized cytosolic proteins to class I‐restricted cytotoxic T lymphocytes. Journal of Experimental Medicine, 175(1), 163–168. 10.1084/jem.175.1.163 PMC2119061 1309852

[178] Dolan, B. P. , Li, L. , Takeda, K. , Bennink, J. R. , & Yewdell, J. W. (2010). Defective ribosomal products are the major source of antigenic peptides endogenously generated from influenza A virus neuraminidase. Journal of Immunology, 184(3), 1419–1424. 10.4049/jimmunol.0901907 PMC2940057 20038640

[179] Croft, N. P. , Purcell, A. W. , & Tscharke, D. C. (2015). Quantifying epitope presentation using mass spectrometry. Molecular Immunology, 68(2 Pt A), 77–80. 10.1016/j.molimm.2015.06.010 26118903

[180] Yang, N. , Gibbs, J. S. , Hickman, H. D. , Reynoso, G. V. , Ghosh, A. K. , Bennink, J. R. , & Yewdell, J. W. (2016). Defining viral defective ribosomal products: Standard and alternative translation initiation events generate a common peptide from influenza A virus M2 and M1 mRNAs. Journal of Immunology, 196(9), 3608–3617. 10.4049/jimmunol.1502303 PMC4868770 27016602

[181] Wei, J. , Zanker, D. , Di Carluccio, A. R. , Smelkinson, M. G. , Takeda, K. , Seedhom, M. O. , Dersh, D. , Gibbs, J. S. , Yang, N. , Jadhav, A. , Chen, W. , & Yewdell, J. W. (2017). Varied role of ubiquitylation in generating MHC class I peptide ligands. Journal of Immunology, 198(10), 3835–3845. 10.4049/jimmunol.1602122 PMC5426817 28363906

[182] Milner, E. , Barnea, E. , Beer, I. , & Admon, A. (2006). The turnover kinetics of major histocompatibility complex peptides of human cancer cells. Molecular and Cellular Proteomics, 5(2), 357–365. 10.1074/mcp.M500241-MCP200 16272561

[183] Lev, A. , Princiotta, M. F. , Zanker, D. , Takeda, K. , Gibbs, J. S. , Kumagai, C. , Waffarn, E. , Dolan, B. P. , Burgevin, A. , Van Endert, P. , Chen, W. , Bennink, J. R. , & Yewdell, J. W. (2010). Compartmentalized MHC class I antigen processing enhances immunosurveillance by circumventing the law of mass action. Proceedings of the National Academy of Sciences of the United States of America, 107(15), 6964–6969. 10.1073/pnas.0910997107 20351281 PMC2872426

[184] Bourdetsky, D. , Schmelzer, C. E. , & Admon, A. (2014). The nature and extent of contributions by defective ribosome products to the HLA peptidome. Proceedings of the National Academy of Sciences of the United States of America, 111(16), E1591‐1599. 10.1073/pnas.1321902111 24715725 PMC4000780

[185] Tomko, R. J., Jr. , & Hochstrasser, M. (2013). Molecular architecture and assembly of the eukaryotic proteasome. Annual Review of Biochemistry, 82, 415–445. 10.1146/annurev-biochem-060410-150257 PMC3827779 23495936

[186] Inobe, T. , & Matouschek, A. (2014). Paradigms of protein degradation by the proteasome. Current Opinion in Structural Biology, 24, 156–164. 10.1016/j.sbi.2014.02.002 24632559 PMC4010099

[187] Sakata, E. , Eisele, M. R. , & Baumeister, W. (2021). Molecular and cellular dynamics of the 26S proteasome. Biochim Biophys Acta Proteins Proteom, 1869(3), 140583. 10.1016/j.bbapap.2020.140583 33321258

[188] Griffin, T. A. , Nandi, D. , Cruz, M. , Fehling, H. J. , Kaer, L. V. , Monaco, J. J. , & Colbert, R. A. (1998). Immunoproteasome assembly: Cooperative incorporation of interferon gamma (IFN‐gamma)‐inducible subunits. Journal of Experimental Medicine, 187(1), 97–104. 10.1084/jem.187.1.97 PMC2199179 9419215

[189] Kingsbury, D. J. , Griffin, T. A. , & Colbert, R. A. (2000). Novel propeptide function in 20 S proteasome assembly influences beta subunit composition. Journal of Biological Chemistry, 275(31), 24156–24162. 10.1074/jbc.M001742200 10816564

[190] De, M. , Jayarapu, K. , Elenich, L. , Monaco, J. J. , Colbert, R. A. , & Griffin, T. A. (2003). Beta 2 subunit propeptides influence cooperative proteasome assembly. Journal of Biological Chemistry, 278(8), 6153–6159. 10.1074/jbc.M209292200 12456675

[191] Wahl, A. , Schafer, F. , Bardet, W. , & Hildebrand, W. H. (2010). HLA class I molecules reflect an altered host proteome after influenza virus infection. Human Immunology, 71(1), 14–22. 10.1016/j.humimm.2009.08.012 19748539 PMC2795087

[192] Herberts, C. A. , Van Gaans‐Van Den Brink, J. , Van Der Heeft, E. D. , Van Wijk, M. , Hoekman, J. , Jaye, A. , Poelen, M. C. M. , Boog, C. J. P. , Roholl, P. J. M. , Whittle, H. , De Jong, A. , & Van Els, C. (2003). Autoreactivity against induced or upregulated abundant self‐peptides in HLA‐A*0201 following measles virus infection. Human Immunology, 64(1), 44–55. 10.1016/S0198-8859(02)00707-3 12507814

[193] Ternette, N. , Block, P. D. , Sánchez‐Bernabéu, Á. , Borthwick, N. , Pappalardo, E. , Abdul‐Jawad, S. , Ondondo, B. , Charles, P. D. , Dorrell, L. , Kessler, B. M. , & Hanke, T. (2015). Early kinetics of the HLA Class I‐associated peptidome of MVA.HIVconsv‐infected cells. Journal of Virology, 89(11), 5760–5771. 10.1128/JVI.03627-14 25810538 PMC4442425

[194] Javitt, A. , Barnea, E. , Kramer, M. P. , Wolf‐Levy, H. , Levin, Y. , Admon, A. , & Merbl, Y. (2019). Pro‐inflammatory Cytokines Alter the Immunopeptidome Landscape by Modulation of HLA‐B Expression. Frontiers in Immunology, 10, 141. 10.3389/fimmu.2019.00141 30833945 PMC6387973

[195] Kalaora, S. , Lee, J. S. , Barnea, E. , Levy, R. , Greenberg, P. , Alon, M. , Yagel, G. , Bar Eli, G. , Oren, R. , Peri, A. , Patkar, S. , Bitton, L. , Rosenberg, S. A. , Lotem, M. , Levin, Y. , Admon, A. , Ruppin, E. , & Samuels, Y. (2020). Immunoproteasome expression is associated with better prognosis and response to checkpoint therapies in melanoma. Nature Communications, 11(1), 896. 10.1038/s41467-020-14639-9 PMC7021791 32060274

[196] Chong, C. , Marino, F. , Pak, H. , Racle, J. , Daniel, R. T. , Muller, M. , Gfeller, D. , Coukos, G. , & Bassani‐Sternberg, M. (2018). High‐throughput and Sensitive Immunopeptidomics Platform Reveals Profound Interferongamma‐Mediated Remodeling of the Human Leukocyte Antigen (HLA) Ligandome. Molecular and Cellular Proteomics, 17(3), 533–548. 10.1074/mcp.TIR117.000383 29242379 PMC5836376

[197] De Verteuil, D. , Muratore‐Schroeder, T. L. , Granados, D. P. , Fortier, M.‐H. , Hardy, M.‐P. , Bramoullé, A. , Caron, É. , Vincent, K. , Mader, S. , Lemieux, S. , Thibault, P. , & Perreault, C. (2010). Deletion of immunoproteasome subunits imprints on the transcriptome and has a broad impact on peptides presented by major histocompatibility complex I molecules. Molecular and Cellular Proteomics, 9(9), 2034–2047. 10.1074/mcp.M900566-MCP200 20484733 PMC2938112

[198] Morel, S. , Lévy, F. , Burlet‐Schiltz, O. , Brasseur, F. , Probst‐Kepper, M. , Peitrequin, A.‐L. , Monsarrat, B. , Van Velthoven, R. , Cerottini, J.‐C. , Boon, T. , Gairin, J. E. , & Van Den Eynde, B. J. (2000). Processing of some antigens by the standard proteasome but not by the immunoproteasome results in poor presentation by dendritic cells. Immunity, 12(1), 107–117. 10661410 10.1016/s1074-7613(00)80163-6

[199] Guillaume, B. , Stroobant, V. , Bousquet‐Dubouch, M.‐P. , Colau, D. , Chapiro, J. , Parmentier, N. , Dalet, A. , & Van Den Eynde, B. J. (2012). Analysis of the processing of seven human tumor antigens by intermediate proteasomes. Journal of Immunology, 189(7), 3538–3547. 10.4049/jimmunol.1103213 22925930

[200] Chapatte, L. , Ayyoub, M. , Morel, S. , Peitrequin, A.‐L. , Lévy, N. , Servis, C. , Van Den Eynde, B. J. , Valmori, D. , & Lévy, F. (2006). Processing of tumor‐associated antigen by the proteasomes of dendritic cells controls in vivo T‐cell responses. Cancer Research, 66(10), 5461–5468. 10.1158/0008-5472.CAN-05-4310 16707475

[201] Cardinaud, S. , Consiglieri, G. , Bouziat, R. , Urrutia, A. , Graff‐Dubois, S. , Fourati, S. , Malet, I. , Guergnon, J. , Guihot, A. , Katlama, C. , Autran, B. , van Endert, P. , Lemonnier, F. A. , Appay, V. , Schwartz, O. , Kloetzel, P. M. , & Moris, A. (2011). CTL escape mediated by proteasomal destruction of an HIV‐1 cryptic epitope. Plos Pathogens, 7(5), e1002049. 10.1371/journal.ppat.1002049 21589903 PMC3093368

[202] Sun, Y. , Sijts, A. J. , Song, M. , Janek, K. , Nussbaum, A. K. , Kral, S. , Schirle, M. , Stevanovic, S. , Paschen, A. , Schild, H. , Kloetzel, P.‐M. , & Schadendorf, D. (2002). Expression of the proteasome activator PA28 rescues the presentation of a cytotoxic T lymphocyte epitope on melanoma cells. Cancer Research, 62(10), 2875–2882. 12019167

[203] Dudek, N. L. , Tan, C. T. , Gorasia, D. G. , Croft, N. P. , Illing, P. T. , & Purcell, A. W. (2012). Constitutive and inflammatory immunopeptidome of pancreatic beta‐cells. Diabetes, 61(11), 3018–3025. 10.2337/db11-1333 22872234 PMC3478525

[204] Marcilla, M. , Cragnolini, J. J. , & Lopez de Castro, J. A. (2007). Proteasome‐independent HLA‐B27 ligands arise mainly from small basic proteins. Molecular and Cellular Proteomics, 6(5), 923–938. 10.1074/mcp.M600302-MCP200 17308301

[205] Garcia‐Medel, N. , Sanz‐Bravo, A. , Barnea, E. , Admon, A. , & Lopez de Castro, J. A. (2012). The origin of proteasome‐inhibitor resistant HLA class I peptidomes: A study with HLA‐A*68:01. Molecular and Cellular Proteomics, 11(1), M111 011486. 10.1074/mcp.M111.011486 PMC3270105 21969608

[206] Vinitsky, A. , Anton, L. C. , Snyder, H. L. , Orlowski, M. , Bennink, J. R. , & Yewdell, J. W. (1997). The generation of MHC class I‐associated peptides is only partially inhibited by proteasome inhibitors: involvement of nonproteasomal cytosolic proteases in antigen processing? Journal of Immunology, 159(2), 554–564. 9218569

[207] Benham, A. M. , Gromme, M. , & Neefjes, J. (1998). Allelic differences in the relationship between proteasome activity and MHC class I peptide loading. Journal of Immunology, 161(1), 83–89. 9647210

[208] Schwarz, K. , De Giuli, R. , Schmidtke, G. , Kostka, S. , Van Den Broek, M. , Kim, KBo , Crews, C. M. , Kraft, R. , & Groettrup, M. (2000). The selective proteasome inhibitors lactacystin and epoxomicin can be used to either up‐ or down‐regulate antigen presentation at nontoxic doses. Journal of Immunology, 164(12), 6147–6157. 10.4049/jimmunol.164.12.6147 PMC2507740 10843664

[209] Luckey, C. J. , Marto, J. A. , Partridge, M. , Hall, Ed , White, F. M. , Lippolis, J. D. , Shabanowitz, J. , Hunt, D. F. , & Engelhard, V. H. (2001). Differences in the expression of human class I MHC alleles and their associated peptides in the presence of proteasome inhibitors. Journal of Immunology, 167(3), 1212–1221. 10.4049/jimmunol.167.3.1212 11466336

[210] Milner, E. , Gutter‐Kapon, L. , Bassani‐Strenberg, M. , Barnea, E. , Beer, I. , & Admon, A. (2013). The effect of proteasome inhibition on the generation of the human leukocyte antigen (HLA) peptidome. Molecular and Cellular Proteomics, 12(7), 1853–1864. 10.1074/mcp.M112.026013 23538226 PMC3708171

[211] Hanada, K.‐I. , Yewdell, J. W. , & Yang, J. C. (2004). Immune recognition of a human renal cancer antigen through post‐translational protein splicing. Nature, 427(6971), 252–256. 10.1038/nature02240 14724640

[212] Vigneron, N. (2004). An antigenic peptide produced by peptide splicing in the proteasome. Science, 304(5670), 587–590. 15001714 10.1126/science.1095522

[213] Paes, W. , Leonov, G. , Partridge, T. , Chikata, T. , Murakoshi, H. , Frangou, A. , Brackenridge, S. , Nicastri, A. , Smith, A. G. , Learn, G. H. , Li, Y. , Parker, R. , Oka, S. , Pellegrino, P. , Williams, I. , Haynes, B. F. , McMichael, A. J. , Shaw, G. M. , Hahn, B. H. , Takiguchi, M. , … Borrow, P. (2019). Contribution of proteasome‐catalyzed peptide cis‐splicing to viral targeting by CD8(+) T cells in HIV‐1 infection. Proceedings of the National Academy of Sciences of the United States of America, 116(49), 24748–24759. 10.1073/pnas.1911622116 31748275 PMC6900506

[214] Faridi, P. , Woods, K. , Ostrouska, S. , Deceneux, C. , Aranha, R. , Duscharla, D. , Wong, S. Q. , Chen, W. , Ramarathinam, S. H. , Lim Kam Sian, T. C. C. , Croft, N. P. , Li, C. , Ayala, R. , Cebon, J. S. , Purcell, A. W. , Schittenhelm, R. B. , & Behren, A. (2020). Spliced peptides and cytokine‐driven changes in the immunopeptidome of melanoma. Cancer Immunology Research, 8(10), 1322–1334. 10.1158/2326-6066.CIR-19-0894 32938616

[215] Warren, E. H. , Vigneron, N. J. , Gavin, M. A. , Coulie, P. G. , Stroobant, V. , Dalet, A. , Tykodi, S. S. , Xuereb, S. M. , Mito, J. K. , Riddell, S. R. , & Van Den Eynde, B. J. (2006). An antigen produced by splicing of noncontiguous peptides in the reverse order. Science, 313(5792), 1444–1447. 16960008 10.1126/science.1130660

[216] Platteel, A. C. , Mishto, M. , Textoris‐Taube, K. , Keller, C. , Liepe, J. , Busch, D. H. , Kloetzel, P. M. , & Sijts, A. J. (2016). CD8(+) T cells of Listeria monocytogenes‐infected mice recognize both linear and spliced proteasome products. European Journal of Immunology, 46(5), 1109–1118. 10.1002/eji.201545989 26909514

[217] Platteel, A. C. M. , Liepe, J. , Textoris‐Taube, K. , Keller, C. , Henklein, P. , Schalkwijk, H. H. , Cardoso, R. , Kloetzel, P. M. , Mishto, M. , & Sijts, A. (2017). Multi‐level strategy for identifying proteasome‐catalyzed spliced epitopes targeted by CD8(+) T cells during bacterial infection. Cell Reports, 20(5), 1242–1253. 10.1016/j.celrep.2017.07.026 28768206

[218] Vigneron, N. , Stroobant, V. , Ferrari, V. , Abi Habib, J. , & Van den Eynde, B. J. (2019). Production of spliced peptides by the proteasome. Molecular Immunology, 113, 93–102. 10.1016/j.molimm.2018.03.030 29650230

[219] Faridi, P. , Dorvash, M. , & Purcell, A. W. (2021). Spliced HLA‐bound peptides: A Black Swan event in immunology. Clinical and Experimental Immunology, 204(2), 179–188. 10.1111/cei.13589 33644851 PMC8062993

[220] Rolfs, Z. , Muller, M. , Shortreed, M. R. , Smith, L. M. , & Bassani‐Sternberg, M. (2019). Comment on “A subset of HLA‐I peptides are not genomically templated: Evidence for cis‐ and trans‐spliced peptide ligands”. Science Immunology, 4(38). 10.1126/sciimmunol.aaw1622 PMC7039048 31420320

[221] Faridi, P. , Li, C. , Ramarathinam, S. H. , Illing, P. T. , Mifsud, N. A. , Ayala, R. , Song, J. , Gearing, L. J. , Croft, N. P. , & Purcell, A. W. (2019). Response to comment on “A subset of HLA‐I peptides are not genomically templated: Evidence for cis‐ and trans‐spliced peptide ligands”. Science Immunology, 4(38). 10.1126/sciimmunol.aaw8457 31420321

[222] Liepe, J. , Marino, F. , Sidney, J. , Jeko, A. , Bunting, D. E. , Sette, A. , Kloetzel, P. M. , Stumpf, M. P. H. , Heck, A. J. R. , & Mishto, M. (2016). A large fraction of HLA class I ligands are proteasome‐generated spliced peptides. Science, 354(6310), 354–358. 10.1126/science.aaf4384 27846572

[223] Faridi, P. , Li, C. , Ramarathinam, S. H. , Vivian, J. P. , Illing, P. T. , Mifsud, N. A. , Ayala, R. , Song, J. , Gearing, L. J. , Hertzog, P. J. , Ternette, N. , Rossjohn, J. , Croft, N. P. , & Purcell, A. W. (2018). A subset of HLA‐I peptides are not genomically templated: Evidence for cis‐ and trans‐spliced peptide ligands. Science Immunology, 3(28)eaar3947. 10.1126/sciimmunol.aar3947 30315122

[224] Paes, W. , Leonov, G. , Partridge, T. , Nicastri, A. , Ternette, N. , & Borrow, P. (2020). Elucidation of the signatures of proteasome‐catalyzed peptide splicing. Frontiers in immunology, 11, 563800. 10.3389/fimmu.2020.563800 33072102 PMC7541919

[225] Faridi, P. , Dorvash, M. , & Purcell, A. W. (2021). Spliced HLA bound peptides:A Black‐Swan event in immunology. Clinical and Experimental Immunology, 204 179–188 10.1111/cei.13589 33644851 PMC8062993

[226] Mylonas, R. , Beer, I. , Iseli, C. , Chong, C. , Pak, H. S. , Gfeller, D. , Coukos, G. , Xenarios, I. , Müller, M. , & Bassani‐Sternberg, M. (2018). Estimating the contribution of proteasomal spliced peptides to the HLA‐I Ligandome. Molecular and Cellular Proteomics, 17(12), 2347–2357. 10.1074/mcp.RA118.000877 30171158 PMC6283289

[227] Admon, A. (2021). Are there indeed spliced peptides in the Immunopeptidome? Molecular and Cellular Proteomics, 20, 100099. 10.1016/j.mcpro.2021.100099 34022431 PMC8724635

[228] Lichti, C. F. (2021). Identification of spliced peptides in pancreatic islets uncovers errors leading to false assignments. Proteomics, 21(7‐8), e2000176. 10.1002/pmic.202000176 33548107

[229] Ebstein, F. , Textoris‐Taube, K. , Keller, C. , Golnik, R. , Vigneron, N. , Van Den Eynde, B. J. , Schuler‐Thurner, B. , Schadendorf, D. , Lorenz, F. K. M. , Uckert, W. , Urban, S. , Lehmann, A. , Albrecht‐Koepke, N. , Janek, K. , Henklein, P. , Niewienda, A. , Kloetzel, P. M. , & Mishto, M. (2016). Proteasomes generate spliced epitopes by two different mechanisms and as efficiently as non‐spliced epitopes. Scientific Reports, 6, 24032. 10.1038/srep24032 27049119 PMC4822137

[230] Murata, S. , Sasaki, K. , Kishimoto, T. , Niwa, S.‐I. , Hayashi, H. , Takahama, Y. , & Tanaka, K. (2007). Regulation of CD8+ T cell development by thymus‐specific proteasomes. Science, 316(5829), 1349–1353. 10.1126/science.1141915 17540904

[231] Nitta, T. , Murata, S. , Sasaki, K. , Fujii, H. , Ripen, A. M. , Ishimaru, N. , Koyasu, S. , Tanaka, K. , & Takahama, Y. (2010). Thymoproteasome shapes immunocompetent repertoire of CD8+ T cells. Immunity, 32(1), 29–40. 10.1016/j.immuni.2009.10.009 20045355

[232] Nitta, T. , Kochi, Y. , Muro, R. , Tomofuji, Y. , Okamura, T. , Murata, S. , Suzuki, H. , Sumida, T. , Yamamoto, K. , & Takayanagi, H. (2017). Human thymoproteasome variations influence CD8 T cell selection. Science Immunology,eaan5165, 2(12). 10.1126/sciimmunol.aan5165 28783658

[233] Murata, S. , Takahama, Y. , Kasahara, M. , & Tanaka, K. (2018). The immunoproteasome and thymoproteasome: functions, evolution and human disease. Nature Immunology, 19(9), 923–931. 10.1038/s41590-018-0186-z 30104634

[234] Ohigashi, I. , Frantzeskakis, M. , Jacques, A. , Fujimori, S. , Ushio, A. , Yamashita, F. , Ishimaru, N. , Yin, Da , Cam, M. , Kelly, M. C. , Awasthi, P. , Takada, K. , & Takahama, Y. (2021). The thymoproteasome hardwires the TCR repertoire of CD8+ T cells in the cortex independent of negative selection. Journal of Experimental Medicine, 218(4), e20201904. 10.1084/jem.20201904 PMC7873839 33555295

[235] Kasahara, M. , Hayashi, M. , Tanaka, K. , Inoko, H. , Sugaya, K. , Ikemura, T. , & Ishibashi, T. (1996). Chromosomal localization of the proteasome Z subunit gene reveals an ancient chromosomal duplication involving the major histocompatibility complex. Proceedings of the National Academy of Sciences of the United States of America, 93(17), 9096–9101. 10.1073/pnas.93.17.9096 8799160 PMC38601

[236] Sutoh, Y. , Kondo, M. , Ohta, Y. , Ota, T. , Tomaru, U. , Flajnik, M. F. , & Kasahara, M. (2012). Comparative genomic analysis of the proteasome beta5t subunit gene: Implications for the origin and evolution of thymoproteasomes. Immunogenetics, 64(1), 49–58. 10.1007/s00251-011-0558-0 21748441 PMC3805029

[237] Schubert, U. , Antón, L. C. , Gibbs, J. , Norbury, C. C. , Yewdell, J. W. , & Bennink, J. R. (2000). Rapid degradation of a large fraction of newly synthesized proteins by proteasomes. Nature, 404(6779), 770–774. 10.1038/35008096 10783891

[238] Reits, E. A. J. , Vos, J. C. , Grommé, M. , & Neefjes, J. (2000). The major substrates for TAP in vivo are derived from newly synthesized proteins. Nature, 404(6779), 774–778. 10.1038/35008103 10783892

[239] Yewdell, J. W. , & Hollý, J. (2020). DRiPs get molecular. Current Opinion in Immunology, 64, 130–136. 10.1016/j.coi.2020.05.009 32615334

[240] Cruz, F. M. , Colbert, J. D. , Merino, E. , Kriegsman, B. A. , & Rock, K. L. (2017). The biology and underlying mechanisms of cross‐presentation of exogenous antigens on MHC‐I molecules. Annual Review of Immunology, 35, 149–176. 10.1146/annurev-immunol-041015-055254 PMC5508990 28125356

[241] Schwab, S. R. , Li, K. C. , Kang, C. , & Shastri, N. (2003). Constitutive display of cryptic translation products by MHC class I molecules. Science, 301(5638), 1367–1371. 10.1126/science.1085650 12958358

[242] Erhard, F. , Dölken, L. , Schilling, B. , & Schlosser, A. (2020). Identification of the cryptic HLA‐I immunopeptidome. Cancer Immunology Research, 8(8), 1018–1026. 10.1158/2326-6066.CIR-19-0886 32561536

[243] Laumont, C. M. , Daouda, T. , Laverdure, J.‐P. , Bonneil, É. , Caron‐Lizotte, O. , Hardy, M.‐P. , Granados, D. P. , Durette, C. , Lemieux, S. , Thibault, P. , & Perreault, C. (2016). Global proteogenomic analysis of human MHC class I‐associated peptides derived from non‐canonical reading frames. Nature Communications, 7, 10238. 10.1038/ncomms10238 PMC4728431 26728094

[244] Shastri, N. , Schwab, S. , & Serwold, T. (2002). Producing nature's gene‐chips: The generation of peptides for display by MHC class I molecules. Annual Review of Immunology, 20, 463–493. 10.1146/annurev.immunol.20.100301.064819 11861610

[245] Erhard, F. , Halenius, A. , Zimmermann, C. , L'hernault, A. , Kowalewski, D. J. , Weekes, M. P. , Stevanovic, S. , Zimmer, R. , & Dölken, L. (2018). Improved Ribo‐seq enables identification of cryptic translation events. Nature Methods, 15(5), 363–366. 10.1038/nmeth.4631 29529017 PMC6152898

[246] Shastri, N. , Nguyen, Vu , & Gonzalez, F. (1995). Major histocompatibility class I molecules can present cryptic translation products to T‐cells. Journal of Biological Chemistry, 270(3), 1088–1091. 10.1074/jbc.270.3.1088 7836364

[247] Malarkannan, S. , Horng, T. , Shih, P. P. , Schwab, S. , & Shastri, N. (1999). Presentation of out‐of‐frame peptide/MHC class I complexes by a novel translation initiation mechanism. Immunity, 10(6), 681–690. 10.1016/s1074-7613(00)80067-9 10403643

[248] Schwab, S. R. , Shugart, J. A. , Horng, T. , Malarkannan, S. , & Shastri, N. (2004). Unanticipated antigens: Translation initiation at CUG with leucine. Plos Biology, 2(11), e366. 10.1371/journal.pbio.0020366 15510226 PMC524250

[249] Starck, S. R. , Jiang, V. , Pavon‐Eternod, M. , Prasad, S. , Mccarthy, B. , Pan, T. , & Shastri, N. (2012). Leucine‐tRNA initiates at CUG start codons for protein synthesis and presentation by MHC class I. Science, 336(6089), 1719–1723. 10.1126/science.1220270 22745432

[250] Starck, S. R. , Tsai, J. C. , Chen, K. , Shodiya, M. , Wang, L. , Yahiro, K. , Martins‐Green, M. , Shastri, N. , & Walter, P. (2016). Translation from the 5' untranslated region shapes the integrated stress response. Science, 351(6272), aad3867. 10.1126/science.aad3867 26823435 PMC4882168

[251] Prasad, S. , Starck, S. R. , & Shastri, N. (2016). Presentation of cryptic peptides by MHC Class I is enhanced by inflammatory stimuli. Journal of Immunology, 197(8), 2981–2991. 10.4049/jimmunol.1502045 PMC5101130 27647836

[252] Sendoel, A. , Dunn, J. G. , Rodriguez, E. H. , Naik, S. , Gomez, N. C. , Hurwitz, B. , Levorse, J. , Dill, B. D. , Schramek, D. , Molina, H. , Weissman, J. S. , & Fuchs, E. (2017). Translation from unconventional 5' start sites drives tumour initiation. Nature, 541(7638), 494–499. 10.1038/nature21036 28077873 PMC5287289

[253] Wei, J. , Kishton, R. J. , Angel, M. , Conn, C. S. , Dalla‐Venezia, N. , Marcel, V. , Vincent, A. , Catez, F. , Ferré, S. , Ayadi, L. , Marchand, V. , Dersh, D. , Gibbs, J. S. , Ivanov, I. P. , Fridlyand, N. , Couté, Y. , Diaz, J.‐J. , Qian, S.‐B. , Staudt, L. M. , … Yewdell, J. W. (2019). Ribosomal proteins regulate MHC class I peptide generation for immunosurveillance. Molecular Cell, 73(6), 1162–1173 e1165. 10.1016/j.molcel.2018.12.020 30712990 PMC6697054

[254] Watson, J. , Baker, T. , Bell, S. , Gann, A. , Levine, M. & Losick, R. (2013). Chapter 15: Translation. In Molecular Biology of the Gene. Cold Spring Harbor Laboratory Press, pp. 509–571.

[255] Cardinaud, S. , Moris, A. , Fevrier, M. , Rohrlich, P. S. , Weiss, L. , Langlade‐Demoyen, P. , Lemonnier, F. A. , Schwartz, O. , & Habel, A. (2004). Identification of cryptic MHC I‐restricted epitopes encoded by HIV‐1 alternative reading frames. Journal of Experimental Medicine, 199(8), 1053–1063. 10.1084/jem.20031869 PMC2211898 15078897

[256] Jagger, B. W. , Wise, H. M. , Kash, J. C. , Walters, K.‐A. , Wills, N. M. , Xiao, Y.‐L. , Dunfee, R. L. , Schwartzman, L. M. , Ozinsky, A. , Bell, G. L. , Dalton, R. M. , Lo, A. , Efstathiou, S. , Atkins, J. F. , Firth, A. E. , Taubenberger, J. K. , & Digard, P. (2012). An overlapping protein‐coding region in influenza A virus segment 3 modulates the host response. Science, 337(6091), 199–204. 10.1126/science.1222213 22745253 PMC3552242

[257] Anton, L. C. , & Yewdell, J. W. (2014). Translating DRiPs: MHC class I immunosurveillance of pathogens and tumors. Journal of Leukocyte Biology, 95(4), 551–562. 10.1189/jlb.1113599 24532645 PMC3958739

[258] Netzer, N. , Goodenbour, J. M. , David, A. , Dittmar, K. A. , Jones, R. B. , Schneider, J. R. , Boone, D. , Eves, E. M. , Rosner, M. R. , Gibbs, J. S. , Embry, A. , Dolan, B. , Das, S. , Hickman, H. D. , Berglund, P. , Bennink, J. R. , Yewdell, J. W. , & Pan, T. (2009). Innate immune and chemically triggered oxidative stress modifies translational fidelity. Nature, 462(7272), 522–526. 10.1038/nature08576 19940929 PMC2785853

[259] Amobi, A. , Qian, F. , Lugade, A. A. , & Odunsi, K. (2017). Tryptophan catabolism and cancer immunotherapy targeting IDO mediated immune suppression. Advances in Experimental Medicine and Biology, 1036, 129–144. 10.1007/978-3-319-67577-0_9 29275469

[260] Labadie, B. W. , Bao, R. , & Luke, J. J. (2019). Reimagining IDO pathway inhibition in cancer immunotherapy via downstream focus on the tryptophan‐kynurenine‐aryl hydrocarbon axis. Clinical Cancer Research, 25(5), 1462–1471. 10.1158/1078-0432.CCR-18-2882 30377198 PMC6397695

[261] Bartok, O. , Pataskar, A. , Nagel, R. , Laos, M. , Goldfarb, E. , Hayoun, D. , Levy, R. , Körner, P.‐R. , Kreuger, I. Z. M. , Champagne, J. , Zaal, E. A. , Bleijerveld, O. B. , Huang, X. , Kenski, J. , Wargo, J. , Brandis, A. , Levin, Y. , Mizrahi, O. , Alon, M. … Agami, R. (2021). Anti‐tumour immunity induces aberrant peptide presentation in melanoma. Nature, 590(7845), 332–337. 10.1038/s41586-020-03054-1 33328638

[262] Coulie, P. G. , Lehmann, F. , Lethe, B. , Herman, J. , Lurquin, C. , Andrawiss, M. , & Boon, T. (1995). A mutated intron sequence codes for an antigenic peptide recognized by cytolytic T lymphocytes on a human melanoma. Proceedings of the National Academy of Sciences of the United States of America, 92(17), 7976–7980. 10.1073/pnas.92.17.7976 7644523 PMC41269

[263] Guilloux, Y. , Lucas, S. , Brichard, V. G. , Van Pel, A. , Viret, C. , De Plaen, E. , Brasseur, F. , Lethé, B. , Jotereau, F. , & Boon, T. (1996). A peptide recognized by human cytolytic T lymphocytes on HLA‐A2 melanomas is encoded by an intron sequence of the N‐acetylglucosaminyltransferase V gene. Journal of Experimental Medicine, 183(3), 1173–1183. 10.1084/jem.183.3.1173 PMC2192325 8642259

[264] Apcher, S. , Millot, G. , Daskalogianni, C. , Scherl, A. , Manoury, B. , & Fahraeus, R. (2013). Translation of pre‐spliced RNAs in the nuclear compartment generates peptides for the MHC class I pathway. Proceedings of the National Academy of Sciences of the United States of America, 110(44), 17951–17956. 10.1073/pnas.1309956110 24082107 PMC3816435

[265] Apcher, S. , Daskalogianni, C. , Lejeune, F. , Manoury, B. , Imhoos, G. , Heslop, L. , & Fahraeus, R. (2011). Major source of antigenic peptides for the MHC class I pathway is produced during the pioneer round of mRNA translation. Proceedings of the National Academy of Sciences of the United States of America, 108(28), 11572–11577. 10.1073/pnas.1104104108 21709220 PMC3136330

[266] Apcher, S. , Prado Martins, R. , & Fåhraeus, R. (2016). The source of MHC class I presented peptides and its implications. Current Opinion in Immunology, 40, 117–122. 10.1016/j.coi.2016.04.002 27105144

[267] Dolan, B. P. , Knowlton, J. J. , David, A. , Bennink, J. R. , & Yewdell, J. W. (2010). RNA polymerase II inhibitors dissociate antigenic peptide generation from normal viral protein synthesis: A role for nuclear translation in defective ribosomal product synthesis? Journal of Immunology, 185(11), 6728–6733. 10.4049/jimmunol.1002543 PMC3398797 21048111

[268] Lu, X. , Gibbs, J. S. , Hickman, H. D. , David, A. , Dolan, B. P. , Jin, Y. , Kranz, D. M. , Bennink, J. R. , Yewdell, J. W. , & Varma, R. (2012). Endogenous viral antigen processing generates peptide‐specific MHC class I cell‐surface clusters. Proceedings of the National Academy of Sciences of the United States of America, 109(38), 15407–15412. 10.1073/pnas.1208696109 22949678 PMC3458372

[269] Yewdell, J. W. , & Nicchitta, C. V. (2006). The DRiP hypothesis decennial: Support, controversy, refinement and extension. Trends in Immunology, 27(8), 368–373. 10.1016/j.it.2006.06.008 16815756

[270] Yewdell, J. W. , Dersh, D. , & Fåhraeus, R. (2019). Peptide Channeling: The Key to MHC Class I Immunosurveillance? Trends in Cell Biology, 29(12), 929–939. 10.1016/j.tcb.2019.09.004 31662235

[271] Wei, J. , & Yewdell, J. W. (2019). Immunoribosomes: Where's there's fire, there's fire. Molecular Immunology, 113, 38–42. 10.1016/j.molimm.2017.12.026 29361306

[272] Vlachos, A. (2017). Acquired ribosomopathies in leukemia and solid tumors. Hematology Am Soc Hematol Educ Program, 2017(1), 716–719. 10.1182/asheducation-2017.1.716 29222326 PMC6142526

[273] Ingolia, N. T. , Lareau, L. F. , & Weissman, J. S. (2011). Ribosome profiling of mouse embryonic stem cells reveals the complexity and dynamics of mammalian proteomes. Cell, 147(4), 789–802. 10.1016/j.cell.2011.10.002 22056041 PMC3225288

[274] Mcglincy, N. J. , & Ingolia, N. T. (2017). Transcriptome‐wide measurement of translation by ribosome profiling. Methods (San Diego, Calif.), 126, 112–129. 10.1016/j.ymeth.2017.05.028 PMC5582988 28579404

[275] Chong, C. , Müller, M. , Pak, H. , Harnett, D. , Huber, F. , Grun, D. , Leleu, M. , Auger, A. , Arnaud, M. , Stevenson, B. J. , Michaux, J. , Bilic, I. , Hirsekorn, A. , Calviello, L. , Simó‐Riudalbas, L. , Planet, E. , Lubiński, J. , Bryśkiewicz, M. , Wiznerowicz, M. … Bassani‐Sternberg, M. (2020). Integrated proteogenomic deep sequencing and analytics accurately identify non‐canonical peptides in tumor immunopeptidomes. Nature Communications, 11(1), 1293. 10.1038/s41467-020-14968-9 PMC7064602 32157095

[276] Ouspenskaia, T. , Law, T. , Clauser, K. , Klaeger, S. , Sarkizova, S. , Aguet, F. , … (2021). Thousands of novel unannotated proteins expand the MHC I immunopeptidome in cancer. bioRxiv, 10.1101/2020.1102.1112.945840. PMC10198624 34663921

[277] Grubaugh, D. , Flechtner, J. B. , & Higgins, D. E. (2013). Proteins as T cell antigens: Methods for high‐throughput identification. Vaccine, 31(37), 3805–3810. 10.1016/j.vaccine.2013.06.046 23806245

[278] Peters, B. , Nielsen, M. , & Sette, A. (2020). T Cell epitope predictions. Annual Review of Immunology, 38, 123–145. 10.1146/annurev-immunol-082119-124838 PMC10878398 32045313

[279] Paul, S. , Croft, N. P. , Purcell, A. W. , Tscharke, D. C. , Sette, A. , Nielsen, M. , & Peters, B. (2020). Benchmarking predictions of MHC class I restricted T cell epitopes in a comprehensively studied model system. Plos Computational Biology, 16(5), e1007757. 10.1371/journal.pcbi.1007757 32453790 PMC7274474

[280] Haj, A. K. , Breitbach, M. E. , Baker, D. A. , Mohns, M. S. , Moreno, G. K. , Wilson, N. A. , Lyamichev, V. , Patel, J. , Weisgrau, K. L. , Dudley, D. M. , & Connor, D. H. (2020). High‐throughput Identification of MHC class I binding peptides using an ultradense peptide array. Journal of Immunology, 204(6), 1689–1696. 10.4049/jimmunol.1900889 PMC7065948 32060132

[281] Osterbye, T. , Nielsen, M. , Dudek, N. L. , Ramarathinam, S. H. , Purcell, A. W. , Schafer‐Nielsen, C. , & Buus, S. (2020). HLA class II specificity assessed by high‐density peptide microarray interactions. Journal of Immunology, 205(1), 290–299. 10.4049/jimmunol.2000224 PMC7313418 32482711

[282] Birnbaum, M. E. , Mendoza, J. L. , Sethi, D. K. , Dong, S. , Glanville, J. , Dobbins, J. , Özkan, E. , Davis, M. M. , Wucherpfennig, K. W. , & Garcia, K. C. (2014). Deconstructing the peptide‐MHC specificity of T cell recognition. Cell, 157(5), 1073–1087. 10.1016/j.cell.2014.03.047 24855945 PMC4071348

[283] Schmidt, J. , Smith, A. R. , Magnin, M. , Racle, J. , Devlin, J. R. , Bobisse, S. , Cesbron, J. , Bonnet, V. , Carmona, S. J. , Huber, F. , Ciriello, G. , Speiser, D. E. , Bassani‐Sternberg, M. , Coukos, G. , Baker, B. M. , Harari, A. , & Gfeller, D. (2021). Prediction of neo‐epitope immunogenicity reveals TCR recognition determinants and provides insight into immunoediting. Cell Reports Medicine, 2(2), 100194. 10.1016/j.xcrm.2021.100194 33665637 PMC7897774

[284] Huang, J. , Brameshuber, M. , Zeng, X. , Xie, J. , Li, Q. J. , Chien, Y. H. , Valitutti, S. , & Davis, M. M. (2013). A single peptide‐major histocompatibility complex ligand triggers digital cytokine secretion in CD4(+) T cells. Immunity, 39(5), 846–857. 10.1016/j.immuni.2013.08.036 24120362 PMC3846396

[285] Pooler, L. M. , Choi, E. Y. , Tranchita, A. M. , Reinbold, C. J. A. , Malarkannan, S. , Brown, A. C. , Shaffer, D. J. , Roopenian, D. C. , & Luedtke, B. (2003). A single nucleotide polymorphism in the Emp3 gene defines the H4 minor histocompatibility antigen. Immunogenetics, 55(5), 284–295. 10.1007/s00251-003-0581-x 12845499

[286] Pettmann, J. , Huhn, A. , Abu Shah, E. , Kutuzov, M. A. , Wilson, D. B. , Dustin, M. L. , Davis, S. J. , Van Der Merwe, P. A. , & Dushek, O. (2021). The discriminatory power of the T cell receptor. Elife, 10. 10.7554/eLife.67092 PMC8219380 34030769

[287] Feng, D. , Bond, C. J. , Ely, L. K. , Maynard, J. , & Garcia, K. C. (2007). Structural evidence for a germline‐encoded T cell receptor‐major histocompatibility complex interaction ‘codon’. Nature Immunology, 8(9), 975–983. 10.1038/ni1502 17694060

[288] Christopher Garcia, K. , Adams, J. J. , Feng, D. , & Ely, L. K. (2009). The molecular basis of TCR germline bias for MHC is surprisingly simple. Nature Immunology, 10(2), 143–147. 10.1038/ni.f.219 19148199 PMC3982143

[289] Marrack, P. , Scott‐Browne, J. P. , Dai, S. , Gapin, L. , & Kappler, J. W. (2008). Evolutionarily conserved amino acids that control TCR‐MHC interaction. Annual Review of Immunology, 26, 171–203. 10.1146/annurev.immunol.26.021607.090421 PMC3164820 18304006

[290] Mazza, C. , Auphan‐Anezin, N. , Gregoire, C. , Guimezanes, A. , Kellenberger, C. , Roussel, A. , Kearney, A. , Van Der Merwe, P. A. , Schmitt‐Verhulst, A.‐M. , & Malissen, B. (2007). How much can a T‐cell antigen receptor adapt to structurally distinct antigenic peptides? EMBO Journal, 26(7), 1972–1983. 10.1038/sj.emboj.7601605 PMC1847653 17363906

[291] Boesteanu, A. , Brehm, M. , Mylin, L. M. , Christianson, G. J. , Tevethia, S. S. , Roopenian, D. C. , & Joyce, S. (1998). A molecular basis for how a single TCR interfaces multiple ligands. Journal of Immunology, 161(9), 4719–4727. 9794402

[292] Wooldridge, L. , Ekeruche‐Makinde, J. , Van Den Berg, H. A. , Skowera, A. , Miles, J. J. , Tan, M. P. , Dolton, G. , Clement, M. , Llewellyn‐Lacey, S. , Price, D. A. , Peakman, M. , & Sewell, A. K. (2012). A single autoimmune T cell receptor recognizes more than a million different peptides. Journal of Biological Chemistry, 287(2), 1168–1177. 10.1074/jbc.M111.289488 PMC3256900 22102287

[293] Morris, G. P. , & Allen, P. M. (2012). How the TCR balances sensitivity and specificity for the recognition of self and pathogens. Nature Immunology, 13(2), 121–128. 10.1038/ni.2190 22261968 PMC13052442

[294] Theodossis, A. , Guillonneau, C. , Welland, A. , Ely, L. K. , Clements, C. S. , Williamson, N. A. , Webb, A. I. , Wilce, J. A. , Mulder, R. J. , Dunstone, M. A. , Doherty, P. C. , Mccluskey, J. , Purcell, A. W. , Turner, S. J. , & Rossjohn, J. (2010). Constraints within major histocompatibility complex class I restricted peptides: presentation and consequences for T‐cell recognition. Proceedings of the National Academy of Sciences of the United States of America, 107(12), 5534–5539. 10.1073/pnas.1000032107 20212169 PMC2851776

[295] Gee, M. H. , Han, A. , Lofgren, S. M. , Beausang, J. F. , Mendoza, J. L. , Birnbaum, M. E. , Bethune, M. T. , Fischer, S. , Yang, X. , Gomez‐Eerland, R. , Bingham, D. B. , Sibener, L. V. , Fernandes, R. A. , Velasco, A. , Baltimore, D. , Schumacher, T. N. , Khatri P., Quake, S. R. , Davis, M. M. , & Garcia, K. C. (2018). Antigen identification for orphan T cell receptors expressed on tumor‐infiltrating lymphocytes. Cell, 172(3), 549–563 e516. 10.1016/j.cell.2017.11.043 29275860 PMC5786495

[296] Glanville, J. , Huang, H. , Nau, A. , Hatton, O. , Wagar, L. E. , Rubelt, F. , Ji, X. , Han, A. , Krams, S. M. , Pettus, C. , Haas, N. , Arlehamn, C. S. L. , Sette, A. , Boyd, S. D. , Scriba, T. J. , Martinez, O. M. , & Davis, M. M. (2017). Identifying specificity groups in the T cell receptor repertoire. Nature, 547(7661), 94–98. 10.1038/nature22976 28636589 PMC5794212

[297] Dash, P. , Fiore‐Gartland, A. J. , Hertz, T. , Wang, G. C. , Sharma, S. , Souquette, A. , Crawford, J. C. , Clemens, E. B. , Nguyen, T. H. O. , Kedzierska, K. , La Gruta, N. L. , Bradley, P. , & Thomas, P. G. (2017). Quantifiable predictive features define epitope‐specific T cell receptor repertoires. Nature, 547(7661), 89–93. 10.1038/nature22383 28636592 PMC5616171

[298] Bousbaine, D. , & Ploegh, H. L. (2020). Antigen discovery tools for adaptive immune receptor repertoire research. Current Opinion in Systems Biology, 24, 64–70. 10.1016/j.coisb.2020.10.002 33195881 PMC7665270

[299] Dhanda, S. K. , Mahajan, S. , Paul, S. , Yan, Z. , Kim, H. , Jespersen, M. C. , Jurtz, V. , Andreatta, M. , Greenbaum, J. A. , Marcatili, P. , Sette, A. , Nielsen, M. , & Peters, B. (2019). IEDB‐AR: Immune epitope database‐analysis resource in 2019. Nucleic Acids Research, 47(W1), W502‐W506. 10.1093/nar/gkz452 31114900 PMC6602498

[300] Flyer, D. C. , Ramakrishna, V. , Miller, C. , Myers, H. , McDaniel, M. , Root, K. , Flournoy, C. , Engelhard, V. H. , Canaday, D. H. , Marto, J. A. , Ross, M. M. , Hunt, D. F. , Shabanowitz, J. , & White, F. M. (2002). Identification by mass spectrometry of CD8(+)‐T‐cell Mycobacterium tuberculosis epitopes within the Rv0341 gene product. Infection and Immunity, 70(6), 2926–2932. 12010981 10.1128/IAI.70.6.2926-2932.2002 PMC127998

[301] Bettencourt, P. , Müller, J. , Nicastri, A. , Cantillon, D. , Madhavan, M. , Charles, P. D. , Fotso, C. B. , Wittenberg, R. , Bull, N. , Pinpathomrat, N. , Waddell, S. J. , Stylianou, E. , Hill, A. V. S. , Ternette, N. , & Mcshane, H. (2020). Identification of antigens presented by MHC for vaccines against tuberculosis. NPJ Vaccines, 5(1), 2. 10.1038/s41541-019-0148-y 31908851 PMC6941960

[302] Darwin, K. H. (2003). The proteasome of Mycobacterium tuberculosis is required for resistance to nitric oxide. Science, 302(5652), 1963–1966. 10.1126/science.1091176 14671303

[303] Samanovic, M. I. , Li, H. , & Darwin, K. H. (2013). The pup‐proteasome system of Mycobacterium tuberculosis. Sub‐Cellular Biochemistry, 66, 267–295. 10.1007/978-94-007-5940-4_10 23479444 PMC4212895

[304] Collins, E. J. , Garboczi, D. N. , & Wiley, D. C. (1994). Three‐dimensional structure of a peptide extending from one end of a class I MHC binding site. Nature, 371(6498), 626–629. 10.1038/371626a0 7935798

[305] Tenzer, S. , Wee, E. , Burgevin, A. , Stewart‐Jones, G. , Friis, L. , Lamberth, K. , Chang, C.‐H. , Harndahl, M. , Weimershaus, M. , Gerstoft, J. , Akkad, N. , Klenerman, P. , Fugger, L. , Jones, E. Y. , Mcmichael, A. J. , Buus, S. , Schild, H. , Van Endert, P. , & Iversen, A. K. N. (2009). Antigen processing influences HIV‐specific cytotoxic T lymphocyte immunodominance. Nature Immunology, 10(6), 636–646. 10.1038/ni.1728 19412183

[306] Guillaume, P. , Picaud, S. , Baumgaertner, P. , Montandon, N. , Schmidt, J. , Speiser, D. E. , Coukos, G. , Bassani‐Sternberg, M. , Filippakopoulos, P. , & Gfeller, D. (2018). The C‐terminal extension landscape of naturally presented HLA‐I ligands. Proceedings of the National Academy of Sciences of the United States of America, 115(20), 5083–5088. 10.1073/pnas.1717277115 29712860 PMC5960288

[307] Urban, R. G. , Chicz, R. M. , Lane, W. S. , Strominger, J. L. , Rehm, A. , Kenter, M. J. , Uytdehaag, F. G. , Ploegh, H. , Uchanska‐Ziegler, B. , & Ziegler, A. (1994). A subset of HLA‐B27 molecules contains peptides much longer than nonamers. Proceedings of the National Academy of Sciences of the United States of America, 91(4), 1534–1538. 10.1073/pnas.91.4.1534 8108441 PMC43194

[308] Joyce, S. , Kuzushima, K. , Kepecs, G. , Angeletti, R. H. , & Nathenson, S. G. (1994). Characterization of an incompletely assembled major histocompatibility class I molecule (H‐2Kb) associated with unusually long peptides: implications for antigen processing and presentation. Proceedings of the National Academy of Sciences of the United States of America, 91(10), 4145–4149. 10.1073/pnas.91.10.4145 8183884 PMC43741

[309] Joyce, S. , & Nathenson, S. G. (1996). Alloreactivity, antigen recognition and T‐cell selection: Three diverse T‐cell recognition problems with a common solution. Immunological Reviews, 154, 59–103. 10.1111/j.1600-065x.1996.tb00930.x 9034864

[310] Tscharke, D. C. , Karupiah, G. , Zhou, J. , Palmore, T. , Irvine, K. R. , Haeryfar, S. M. M. , Williams, S. , Sidney, J. , Sette, A. , Bennink, J. R. , & Yewdell, J. W. (2005). Identification of poxvirus CD8+ T cell determinants to enable rational design and characterization of smallpox vaccines. Journal of Experimental Medicine, 201(1), 95–104. 10.1084/jem.20041912 PMC2212779 15623576

[311] Moutaftsi, M. , Peters, B. , Pasquetto, V. , Tscharke, D. C. , Sidney, J. , Bui, H. H. , Grey, H. , & Sette, A. (2006). A consensus epitope prediction approach identifies the breadth of murine T(CD8+)‐cell responses to vaccinia virus. Nature Biotechnology, 24(7), 817–819. 10.1038/nbt1215 16767078

[312] Oseroff, C. , Kos, F. , Bui, H.‐H. , Peters, B. , Pasquetto, V. , Glenn, J. , Palmore, T. , Sidney, J. , Tscharke, D. C. , Bennink, J. R. , Southwood, S. , Grey, H. M. , Yewdell, J. W. , & Sette, A. (2005). HLA class I‐restricted responses to vaccinia recognize a broad array of proteins mainly involved in virulence and viral gene regulation. Proceedings of the National Academy of Sciences of the United States of America, 102(39), 13980–13985. 10.1073/pnas.0506768102 16172378 PMC1236582

[313] Terajima, M. , Orphin, L. , Leporati, A. M. , Pazoles, P. , Cruz, J. , Rothman, A. L. , & Ennis, F. A. (2008). Vaccinia virus‐specific CD8(+) T‐cell responses target a group of epitopes without a strong immunodominance hierarchy in humans. Human Immunology, 69(12), 815–825. 10.1016/j.humimm.2008.09.009 18955096 PMC2638498

[314] Gilchuk, P. , Hill, T. M. , Wilson, J. T. , & Joyce, S. (2015). Discovering protective CD8 T cell epitopes—No single immunologic property predicts it! Current Opinion in Immunology, 34, 43–51. 10.1016/j.coi.2015.01.013 25660347 PMC5023008

[315] Attig, J. , Young, G. R. , Hosie, L. , Perkins, D. , Encheva‐Yokoya, V. , Stoye, J. P. , Snijders, A. P. , Ternette, N. , & Kassiotis, G. (2019). LTR retroelement expansion of the human cancer transcriptome and immunopeptidome revealed by de novo transcript assembly. Genome Research, 29(10), 1578–1590. 10.1101/gr.248922.119 31537638 PMC6771403

[316] Saini, S. K. , Ørskov, A. D. , Bjerregaard, A.‐M. , Unnikrishnan, A. , Holmberg‐Thydén, S. , Borch, A. , Jensen, K. V. , Anande, G. , Bentzen, A. K. , Marquard, A. M. , Tamhane, T. , Treppendahl, M. B. , Gang, A. O. , Dufva, I. H. , Szallasi, Z. , Ternette, N. , Pedersen, A. G. , Eklund, A. C. , Pimanda, J. …, Hadrup, S. R. (2020). Human endogenous retroviruses form a reservoir of T cell targets in hematological cancers. Nature Communications, 11(1), 5660. 10.1038/s41467-020-19464-8 PMC7653045 33168830

[317] Laumont, C. M. , Vincent, K. , Hesnard, L. , Audemard, É. , Bonneil, É. , Laverdure, J.‐P. , Gendron, P. , Courcelles, M. , Hardy, M.‐P. , Côté, C. , Durette, C. , St‐Pierre, C. , Benhammadi, M. , Lanoix, J. , Vobecky, S. , Haddad, E. , Lemieux, S. , Thibault, P. , & Perreault, C. (2018). Noncoding regions are the main source of targetable tumor‐specific antigens. Science Translational Medicine, 10(470), eaau5516. 10.1126/scitranslmed.aau5516 30518613

[318] Kong, Yu , Rose, C. M. , Cass, A. A. , Williams, A. G. , Darwish, M. , Lianoglou, S. , Haverty, P. M. , Tong, A.‐J. , Blanchette, C. , Albert, M. L. , Mellman, I. , Bourgon, R. , Greally, J. , Jhunjhunwala, S. , & Chen‐Harris, H. (2019). Transposable element expression in tumors is associated with immune infiltration and increased antigenicity. Nature Communications, 10(1), 5228. 10.1038/s41467-019-13035-2 PMC6864081 31745090

[319] Reynisson, B. , Alvarez, B. , Paul, S. , Peters, B. , & Nielsen, M. (2020). NetMHCpan‐4.1 and NetMHCIIpan‐4.0: improved predictions of MHC antigen presentation by concurrent motif deconvolution and integration of MS MHC eluted ligand data. Nucleic Acids Research, 48(W1), W449‐W454. 10.1093/nar/gkaa379 32406916 PMC7319546

[320] Carreno, B. M. , Magrini, V. , Becker‐Hapak, M. , Kaabinejadian, S. , Hundal, J. , Petti, A. A. , Ly, A. , Lie, W.‐R. , Hildebrand, W. H. , Mardis, E. R. , & Linette, G. P. (2015). Cancer immunotherapy. A dendritic cell vaccine increases the breadth and diversity of melanoma neoantigen‐specific T cells. Science, 348(6236), 803–808. 10.1126/science.aaa3828 25837513 PMC4549796

[321] Ott, P. A. , Hu, Z. , Keskin, D. B. , Shukla, S. A. , Sun, J. , Bozym, D. J. , Zhang, W. , Luoma, A. , Giobbie‐Hurder, A. , Peter, L. , Chen, C. , Olive, O. , Carter, T. A. , Li, S. , Lieb, D. J. , Eisenhaure, T. , Gjini, E. , Stevens, J. , Lane, W. J. , …, Wu, C. J. (2017). An immunogenic personal neoantigen vaccine for patients with melanoma. Nature, 547(7662), 217–221. 10.1038/nature22991 28678778 PMC5577644

[322] Sahin, U. , Derhovanessian, E. , Miller, M. , Kloke, B.‐P. , Simon, P. , Löwer, M. , Bukur, V. , Tadmor, A. D. , Luxemburger, U. , Schrörs, B. , Omokoko, T. , Vormehr, M. , Albrecht, C. , Paruzynski, A. , Kuhn, A. N. , Buck, J. , Heesch, S. , Schreeb, K. H. , Müller, F. … Türeci, Ö. (2017). Personalized RNA mutanome vaccines mobilize poly‐specific therapeutic immunity against cancer. Nature, 547(7662), 222–226. 10.1038/nature23003 28678784

[323] Hilf, N. , Kuttruff‐Coqui, S. , Frenzel, K. , Bukur, V. , Stevanović, S. , Gouttefangeas, C. , Platten, M. , Tabatabai, G. , Dutoit, V. , Van Der Burg, S. H. , Thor Straten, P. , Martínez‐Ricarte, F. , Ponsati, B. , Okada, H. , Lassen, U. , Admon, A. , Ottensmeier, C. H. , Ulges, A. , Kreiter, S. … Wick, W. (2019). Actively personalized vaccination trial for newly diagnosed glioblastoma. Nature, 565(7738), 240–245. 10.1038/s41586-018-0810-y 30568303

[324] Keskin, D. B. , Anandappa, A. J. , Sun, J. , Tirosh, I. , Mathewson, N. D. , Li, S. , Oliveira, G. , Giobbie‐Hurder, A. , Felt, K. , Gjini, E. , Shukla, S. A. , Hu, Z. , Li, L. , Le, P. M. , Allesøe, R. L. , Richman, A. R. , Kowalczyk, M. S. , Abdelrahman, S. , Geduldig, J. E. … Reardon, D. A. (2019). Neoantigen vaccine generates intratumoral T cell responses in phase Ib glioblastoma trial. Nature, 565(7738), 234–239. 10.1038/s41586-018-0792-9 30568305 PMC6546179

[325] Fang, Y. , Mo, F. , Shou, J. , Wang, H. , Luo, K. , Zhang, S. , Han, N. , Li, H. , Ye, S. , Zhou, Z. , Chen, R. , Chen, L. , Liu, L. , Wang, H. , Pan, H. , & Chen, S. (2020). A pan‐cancer clinical study of personalized neoantigen vaccine monotherapy in treating patients with various types of advanced solid tumors. Clinical Cancer Research, 26(17), 4511–4520. 10.1158/1078-0432.CCR-19-2881 32439700

[326] Robbins, P. F. , Lu, Y. C. , El‐Gamil, M. , Li, Y. F. , Gross, C. , Gartner, J. , Lin, J. C. , Teer, J. K. , Cliften, P. , Tycksen, E. , Samuels, Y. , & Rosenberg, S. A. (2013). Mining exomic sequencing data to identify mutated antigens recognized by adoptively transferred tumor‐reactive T cells. Nature Medicine, 19(6), 747–752. 10.1038/nm.3161 PMC3757932 23644516

[327] Tran, E. , Ahmadzadeh, M. , Lu, Y. C. , Gros, A. , Turcotte, S. , Robbins, P. F. , Gartner, J. J. , Zheng, Z. , Li, Y. F. , Ray, S. , Wunderlich, J. R. , Somerville, R. P. , & Rosenberg, S. A. (2015). Immunogenicity of somatic mutations in human gastrointestinal cancers. Science, 350(6266), 1387–1390. 10.1126/science.aad1253 26516200 PMC7445892

[328] Bai, P. , Zhou, Q. , Wei, P. , Bai, H. , Chan, S. K. , Kappler, J. W. , Marrac, P. , & Yin, L. (2021). Rational discovery of a cancer neoepitope harboring the KRAS G12D driver mutation. Science China Life Sciences. 10.1007/s11427-020-1888-1 33740187

[329] Ivanova, M. , Tsvetkova, G. , Lukanov, T. , Stoimenov, A. , Hadjiev, E. , & Shivarov, V. (2020). Probable HLA‐mediated immunoediting of JAK2 V617F‐driven oncogenesis. Experimental Hematology, 92, 75–88 e10. 10.1016/j.exphem.2020.09.200 33017633

[330] Cafri, G. , Gartner, J. J. , Zaks, T. , Hopson, K. , Levin, N. , Paria, B. C. , Parkhurst, M. R. , Yossef, R. , Lowery, F. J. , Jafferji, M. S. , Prickett, T. D. , Goff, S. L. , McGowan, C. T. , Seitter, S. , Shindorf, M. L. , Parikh, A. , Chatani, P. D. , Robbins, P. F. … Rosenberg, S. A. (2020). mRNA vaccine‐induced neoantigen‐specific T cell immunity in patients with gastrointestinal cancer. Journal of Clinical Investigation, 130(11), 5976–5988. 10.1172/JCI134915 PMC7598064 33016924

[331] Frankiw, L. , Baltimore, D. , & Li, G. (2019). Alternative mRNA splicing in cancer immunotherapy. Nature Reviews Immunology, 19(11), 675–687. 10.1038/s41577-019-0195-7 31363190

[332] Wang, T. Y. , Liu, Q. , Ren, Y. , Alam, S. K. , Wang, L. , Zhu, Z. , Hoeppner, L. H. , Dehm, S. M. , Cao, Q. , & Yang, R. (2021). A pan‐cancer transcriptome analysis of exitron splicing identifies novel cancer driver genes and neoepitopes. Molecular Cell, 81(10), 2246–2260 e2212. 10.1016/j.molcel.2021.03.028 33861991 PMC8141048

[333] Maby, P. , Bindea, G. , Mlecnik, B. , & Galon, J. (2021). License to kill: Microsatellite instability and immune contexture. Oncoimmunology, 10(1), 1905935. 10.1080/2162402X.2021.1905935 33868790 PMC8023238

[334] Roudko, V. , Bozkus, C. C. , Orfanelli, T. , McClain, C. B. , Carr, C. , O'Donnell, T. , Chakraborty, L. , Samstein, R. , Huang, K.‐L. , Blank, S. V. , Greenbaum, B. , & Bhardwaj, N. (2020). Shared immunogenic poly‐epitope frameshift mutations in microsatellite unstable tumors. Cell, 183(6), 1634–1649 e1617. 10.1016/j.cell.2020.11.004 33259803 PMC8025604

[335] Devlin, J. R. , Alonso, J. A. , Ayres, C. M. , Keller, G. L. J. , Bobisse, S. , Vander Kooi, C. W. , Coukos, G. , Gfeller, D. , Harari, A. , & Baker, B. M. (2020). Structural dissimilarity from self drives neoepitope escape from immune tolerance. Nature Chemical Biology, 16(11), 1269–1276. 10.1038/s41589-020-0610-1 32807968 PMC8210748

[336] Yamamoto, T. N. , Kishton, R. J. , & Restifo, N. P. (2019). Developing neoantigen‐targeted T cell‐based treatments for solid tumors. Nature Medicine, 25(10), 1488–1499. 10.1038/s41591-019-0596-y 31591590

[337] Yamauchi, T. , Hoki, T. , Oba, T. , Kajihara, R. , Attwood, K. , Cao, X. , & Ito, F. (2021). CD40 and CD80/86 signaling in cDC1s mediate effective neoantigen vaccination and generation of antigen‐specific CX3CR1(+) CD8(+) T cells. Cancer Immunology, Immunotherapy. 10.1007/s00262-021-02969-6 PMC8715856 34037810

[338] Becker, J. P. , Helm, D. , Rettel, M. , Stein, F. , Hernandez‐Sanchez, A. , Urban, K. , Gebert, J. , Kloor, M. , Neu‐Yilik, G. , von Knebel Doeberitz, M. , Hentze, M. W. , & Kulozik, A. E. (2021). NMD inhibition by 5‐azacytidine augments presentation of immunogenic frameshift‐derived neoepitopes. iScience, 24(4), 102389. 10.1016/j.isci.2021.102389 33981976 PMC8082087

[339] Lu, S. X. , De Neef, E. , Thomas, J. D. , Sabio, E. , Rousseau, B. , Gigoux, M. , Knorr, D. A. , Greenbaum, B. , Elhanati, Y. , Hogg, S. J. , Chow, A. , Ghosh, A. , Xie, A. , Zamarin, D. , Cui, D. , Erickson, C. , Singer, M. , Cho, H. , Wang, E. , Lu, B. … Bradley, R. K. (2021). Pharmacologic modulation of RNA splicing enhances anti‐tumor immunity. Cell, 184, 4032‐4047.e31 10.1016/j.cell.2021.05.038 34171309 PMC8684350

[340] Alspach, E. , Lussier, D. M. , Miceli, A. P. , Kizhvatov, I. , DuPage, M. , Luoma, A. M. , Meng, W. , Lichti, C. F. , Esaulova, E. , Vomund, A. N. , Runci, D. , Ward, J. P. , Gubin, M. M. , Medrano, R. F. V. , Arthur, C. D. , White, J. M. , Sheehan, K. C. F. , Chen, A. , Wucherpfennig, K. W. … Schreiber, R. D. (2019). MHC‐II neoantigens shape tumour immunity and response to immunotherapy. Nature, 574(7780), 696–701. 10.1038/s41586-019-1671-8 31645760 PMC6858572

[341] Wan, X. , Vomund, A. N. , Peterson, O. J. , Chervonsky, A. V. , Lichti, C. F. , & Unanue, E. R. (2020). The MHC‐II peptidome of pancreatic islets identifies key features of autoimmune peptides. Nature Immunology, 21(4), 455–463. 10.1038/s41590-020-0623-7 32152506 PMC7117798

[342] Jin, N. , Wang, Y. , Crawford, F. , White, J. , Marrack, P. , Dai, S. , & Kappler, J. W. (2015). N‐terminal additions to the WE14 peptide of chromogranin A create strong autoantigen agonists in type 1 diabetes. Proceedings of the National Academy of Sciences of the United States of America, 112(43), 13318–13323. 10.1073/pnas.1517862112 26453556 PMC4629350

[343] Wang, Y. , Sosinowski, T. , Novikov, A. , Crawford, F. , Neau, D. B. , Yang, J. , Kwok, W. W. , Marrack, P. , Kappler, J. W. , & Dai, S. (2018). C‐terminal modification of the insulin B:11‐23 peptide creates superagonists in mouse and human type 1 diabetes. Proceedings of the National Academy of Sciences of the United States of America, 115(1), 162–167. 10.1073/pnas.1716527115 29255035 PMC5776820

[344] Wang, Y. , Sosinowski, T. , Novikov, A. , Crawford, F. , White, J. , Jin, N. , Liu, Z. , Zou, J. , Neau, D. , Davidson, H. W. , Nakayama, M. , Kwok, W. W. , Gapin, L. , Marrack, P. , Kappler, J. W. , & Dai, S. (2019). How C‐terminal additions to insulin B‐chain fragments create superagonists for T cells in mouse and human type 1 diabetes. Science Immunology, 4(34). 10.1126/sciimmunol.aav7517 PMC6929690 30952805

[345] Reed, B. , Crawford, F. , Hill, R. C. , Jin, N. , White, J. , Krovi, S. H. , Marrack, P. , Hansen, K. , & Kappler, J. W. (2021). Lysosomal cathepsin creates chimeric epitopes for diabetogenic CD4 T cells via transpeptidation. Journal of Experimental Medicine, 218(2). 10.1084/jem.20192135 PMC7590512 33095259

[346] Khodadoust, M. S. , Olsson, N. , Wagar, L. E. , Haabeth, O. A. , Chen, B. , Swaminathan, K. , Rawson, K. , Long Liu, C. , Steiner, D. , Lund, P. , Rao, S. , Zhang, L. , Marceau, C. , Stehr, H. , Newman, A. M. , Czerwinski, D. K. , Carlton, V. E. H. , Moorhead, M. , Faham, M. … Alizadeh, A. A. (2017). Antigen presentation profiling reveals recognition of lymphoma immunoglobulin neoantigens. Nature, 543(7647), 723–727. 10.1038/nature21433 28329770 PMC5808925

[347] Gilchuk, P. , Knight, F. C. , Wilson, J. T. , & Joyce, S. (2017). Eliciting epitope‐specific CD8+ T cell response by immunization with microbial protein antigens formulated with alpha‐galactosylceramide: theory, practice, and protocols. Methods in Molecular Biology, 1494, 321–352. 10.1007/978-1-4939-6445-1_25 27718206 PMC5086083

[348] Gilchuk, P. , Hill, T. M. , Guy, C. , McMaster, S. R. , Boyd, K. L. , Rabacal, W. A. , Lu, P. , Shyr, Y. , Kohlmeier, J. E. , Sebzda, E. , Green, D. R. , & Joyce, S. (2016). A distinct lung‐interstitium‐resident memory CD8(+) T cell subset confers enhanced protection to lower respiratory tract infection. Cell Reports, 16(7), 1800–1809. 10.1016/j.celrep.2016.07.037 27498869 PMC5021515

